# Insights into the effects of gut microbiota and circulating metabolites on oral cancer: Mendelian randomization analysis and clinical validation

**DOI:** 10.3389/fnut.2026.1827331

**Published:** 2026-05-21

**Authors:** Junfeng Guo, Junfeng Guo, Sui Yang, Yuhao Guo, Junjie Huang, Pengfei Li, Yufang Tang, Yang Yang, Weiwei Zheng, Haitao He

**Affiliations:** 1 Department of Stomatology, Xinqiao Hospital, Army Medical University, Chongqing, China; 2 Department of Stomatology, The 970th Hospital of the Joint Logistics Support Force, Yantai, China; 3 Department of Gastroenterology, Xinqiao Hospital, Army Medical University, Chongqing, China; 4 Department of Stomatology, The 900th Hospital of the Joint Logistics Support Force, Fuzhou, China

**Keywords:** circulating metabolites, gut microbiota, insight, Mendelian randomization, oral cancer

## Abstract

**Background:**

Prior studies have indicated that alterations in the gut microbiota could potentially influence the occurrence of oral cancer. However, the causal relationship between gut microbiota and oral cancer, as well as whether circulating metabolites serve as mediators, remains unclear.

**Methods:**

We employed bidirectional two-sample Mendelian randomization (MR) to explore the causal relationships between gut microbiota, circulating metabolites, and oral cancer. The inverse-variance weighting MR (IVW-MR) and generalized summary data-based MR (GSMR) were used as the primary methods, with various other MR methods used for supplementary analyses. Additionally, we explored the potential mediating effect of circulating metabolites in this relationship. The data on 471 gut microbial taxa, 233 circulating metabolites, and oral cancer used in this study were derived from their respective genome-wide association studies (GWAS). Additional clinical samples were used to validate the Mendelian randomization findings.

**Results:**

The IVW-MR and GSMR analyses identified 15 gut microbial taxa positively associated with oral cancer (OR > 1, *P* < 0.05) and 20 gut microbial taxa negatively associated with oral cancer (OR < 1, *P* < 0.05), with no evidence of horizontal pleiotropy (*P* > 0.05), heterogeneity (*P* > 0.05), or reverse causality. Additionally, five positive (OR > 1, *P* < 0.05) and fifteen negative (OR < 1, *P* < 0.05) causal relationships were found between circulating metabolites and oral cancer, also showing no evidence of horizontal pleiotropy (*P* > 0.05), heterogeneity (*P* > 0.05), or reverse causality. Our study also revealed that the positive effect of *Akkermansia muciniphila* on oral cancer is mediated by citrate, while the negative effect of *Veillonella* on oral cancer is mediated through omega-3 fatty acids. These findings were supported by our clinical multi-omics profiling.

**Conclusions:**

Oral cancer was found to be causally linked to the gut microbiota, with circulating metabolites acting as mediating factors in this association.

## 1 Introduction

Considered a major public health issue, oral cancer ranks among the most prevalent malignant neoplasms worldwide (1). With high morbidity, mortality, and a poor prognosis, the five-year survival rate for patients with oral cancer is approximately 50% and can drop as low as 15%, depending on the stage at diagnosis (2). Furthermore, there is a growing body of epidemiological evidence indicating an increase in the incidence of oral cancer among younger populations worldwide (3). Alcohol consumption, cigarette smoking, genetics, and environmental factors are widely recognized as major risk factors for the development of oral cancer (4–6). Among environmental factors, the significance of microorganisms in cancer biology is gaining recognition (7).

Microbial communities have inhabited the Earth's surface for three-quarters of its history (8). They have evolved into various specialized lineages to adapt to diverse habitats, playing crucial roles in shaping the evolution of modern life (9). The appearance of eukaryotes and multicellular organisms over a billion years ago necessitated their coevolution with microbes, resulting in enduring relationships marked by mutualism, commensalism, parasitism, and mutual dependency for survival and the maintenance of homeostasis (10, 11). This historical record is distinctly inscribed within the genomes of eukaryotic hosts and their respective microorganisms (11). Commensal microorganisms, including protists, archaea, eubacteria, fungi, and viruses, populate all epithelial barrier surfaces of the human body, with bacteria numbering approximately as many as human cells (12, 13). These microorganisms collectively form the human microbiota, and participate in the regulation of various physiological processes in the human body, including those related to cancer (14). The gut microbiota serves as the largest reservoir of microbes within the human body, playing a pivotal role in regulating host metabolism (15). It has emerged as a pivotal player in sustaining human health, exerting its influence not only on the gastrointestinal tract but also on distant organs, including the brain, liver, and oral cavity (16–18). The occurrence of dysbiosis, characterized by disturbances in the composition and function of the gut microbiome, contributes to the development of various pathological conditions, such as obesity (19, 20), diabetes (21, 22), and cancer (23, 24). Recent studies have highlighted the role of the oral-gut axis in systemic diseases (25–27). However, the relationship between gut microbiota and oral cancer remains poorly defined. Further comprehensive research is imperative to explore the causal relationship between gut microbiota and oral cancer, as well as to unveil the potential mechanisms underlying this association.

Cancer induces a variety of metabolic alterations, leading to the reshaping of cellular metabolic pathways (28). Changes in circulating metabolites that reflect specific metabolic phenotypes and tumor behavior hold potential as biomarkers for early cancer diagnosis (29). Studies have confirmed the existence of associations between the gut microbiota and various circulating metabolites (30–32). However, the potential for alterations in gut microbiota to impact the development of oral cancer via modulation of circulating metabolite levels remains unreported. Our study aims to uncover these relationships and identify potential circulating metabolites that could function as crucial tools for early diagnosis and clinical treatment strategies.

Mendelian randomization (MR) serves as a research methodology within epidemiological studies for evaluating causal inferences concerning disease etiology (33). It employs genetic variations to interrogate the potential causal effects of modifiable exposures on developmental or health outcomes (34, 35). MR is also often referred to as a “natural experiment,” leveraging inherent genetic variations to investigate the impact of specific conditions on a particular trait (36). Conventional MR, including inverse-variance weighted (IVW), MR Egger, weighted median, simple mode, weighted mode, and sensitivity analyses, was applied to ensure the robustness of the results. Compared with conventional MR, a novel MR approach, generalized summary data-based MR (GSMR), offers distinct advantages by improving power through the explicit modeling of linkage disequilibrium (LD) among genetic variants (37). Moreover, it employs the Heterogeneity in Dependent Instruments (HEIDI) test to remove pleiotropic single-nucleotide polymorphisms (SNPs) and identify instrumental outliers (38). Genetic variants act as instruments or proxies for exposures that are either impossible or highly difficult to manipulate within the target population under investigation. And then, mediation analysis can be used to investigate whether the exposure mediates the relationship between the instrumental variables (IVs) and the trait of interest (34, 39). In light of previous epidemiological studies that have not conclusively established a causal relationship between gut microbiota and oral cancer, we utilized genotypic and phenotypic data from large-scale genome-wide association studies (GWAS) to conduct bidirectional two-sample MR and mediation analysis. Our study aimed to unravel the complex causal association between them.

## 2 Materials and methods

### 2.1 MR study design

**Figure 1:**
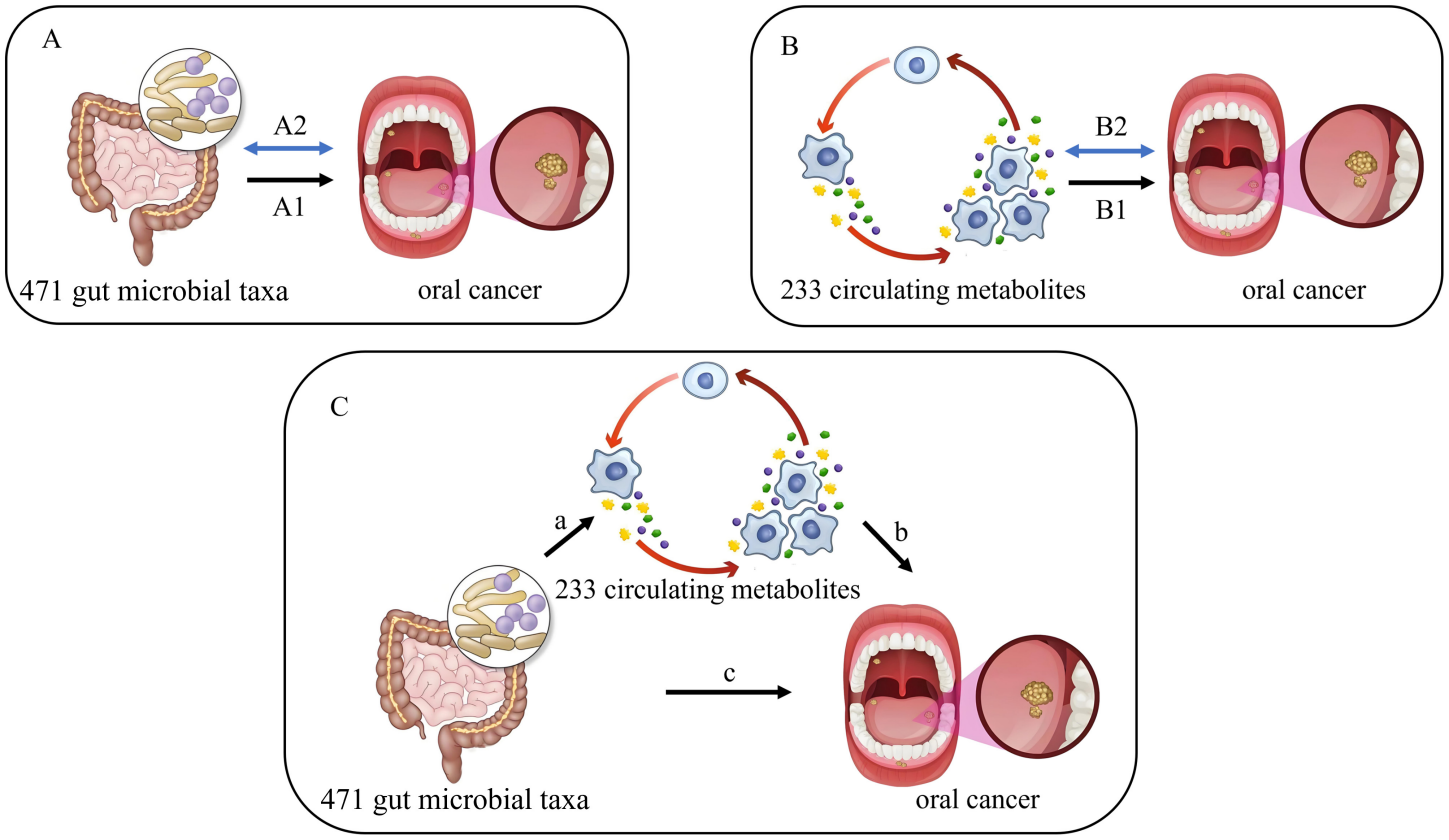
Overview of our study. **(A)** The causal relationship between 471 gut microbial taxa and oral cancer. **(B)** The causal relationship between 233 circulating metabolites and oral cancer. A1 and B1 represent the causal effects of gut microbiota and circulating metabolites on oral cancer, respectively. A2 and B2 represent the bidirectional causality effects of gut microbiota and circulating metabolites on oral cancer, respectively. **(C)** The mediating analysis of 233 circulating metabolites in the pathway from 471 gut microbial taxa to oral cancer: path a was the causal effect of gut microbiota on circulating metabolites. path b was the causal effect of circulating metabolites on oral cancer. path c was the total effect of gut microbiota on oral cancer.

This study comprises three main components. Firstly, a bidirectional two-sample MR was conducted to analyze the causal relationship between 471 gut microbial taxa and oral cancer (Figure 1A). Secondly, the bidirectional two-sample MR was employed to investigate the causal relationship between 233 circulating metabolites and oral cancer (Figure 1B). Lastly, mediation analysis was utilized to explore the potential role of circulating metabolites in the relationship between gut microbiota and oral cancer (Figure 1C). The effectiveness of MR analysis relies on three core assumptions: (1) the IVs are independent of any confounding variables; (2) the IVs are strongly associated with the exposure; (3) the IVs affect the outcome only through the exposure (40).

### 2.2 Data sources for exposure and outcomes

The genetic data related to the gut microbiome were derived from the most recent aggregated GWAS summary-level data. In this study, genome-wide association tests were performed on 7,967,866 human genetic variants and 2,801 microbial taxa across 5,959 individuals. They found 471 distinct Genome Taxonomy Database (GTDB) taxa, spanning 209 species, 146 genera, 62 families, 24 orders, 19 classes, and 11 phyla (41). The genetic data for circulating metabolites were obtained from the most recent GWAS summary-level data, comprising 233 metabolic traits from 136,016 participants, incorporating up to 13,389,637 estimated autosomal single nucleotide polymorphisms (SNPs) (42).

The GWAS Catalog (https://www.ebi.ac.uk/gwas/) provides summary-level data for oral cancer, with a total of 1,832,935 participants from the United Kingdom, United States, Canada, Netherlands, Italy, Greece, Germany, France, Czech Republic, Croatia, Sweden, Spain, Slovakia, Russian Federation, Romania, Republic of Ireland, Poland, and Norway. In order to minimize collider bias and facilitate comparisons at the population level, individuals without oral cancer were included as controls for all outcomes. Detailed sources for all GWAS summary data are provided in Supplementary Table S1.

### 2.3 Selection of instrumental variables

The IVs were selected based on the following criteria: (1) SNPs that met the locus-wide significance threshold (*P* < 1 × 10^−5^) for each genus were identified as potential IVs (33, 36). (2) SNPs associated with linkage disequilibrium (LD) were removed. Among the SNPs with an r^2^ value of less than 0.001 within a clumping window size of 10,000 kb, only those with the most significant *P*-values were retained. (3) SNPs with a minor allele frequency greater than 0.01 were retained, while those with F-statistics less than 10 were excluded. (4) Palindromic SNPs were also excluded.

### 2.4 Clinical validation

#### 2.4.1 Study population and sample collection

We recruited a total of 12 patients diagnosed with oral cancer and 12 matched healthy controls who had not received any treatment before sampling. All participants were recruited from our hospital between July and October 2025. To ensure comparability between groups, we maintained consistency in all factors other than oral cancer, including gender, age, lifestyle, and dietary habits. Healthy controls were recruited from the physical examination center of our hospital. This study was approved by the Ethics Committee of the Second Affiliated Hospital of the Army Medical University (2025-yan-177-01) and was registered with the Chinese Clinical Trial Registry (ChiCTR2500105398).

For all participants, the fecal samples were obtained from the remaining sample after routine stool testing, and the serum samples were obtained from the remaining sample after routine blood testing.

#### 2.4.2 Metagenomic sequencing

Using the QIAamp PowerFecal Pro DNA kit, DNA was extracted from fecal samples. The extracted DNA was then sequenced on the Illumina NovaSeq platform with a read length of 150 bp. Quality control and host DNA depletion were performed before using Kraken2 for taxonomic classification and HUMAnN3 for functional annotation, enabling further analysis of the microbial community.

#### 2.4.3 Targeted metabolomics

Using targeted metabolomics methods, the collected serum samples were processed and analyzed. Metabolites were extracted with methanol and quantified using liquid chromatography-tandem mass spectrometry (LC-MS/MS) in multiple reaction monitoring (MRM) mode. Peak integration and quantification were performed using authentic chemical standards, and data normalization was conducted to ensure analytical accuracy across batches.

### 2.5 Statistical analysis

#### 2.5.1 Primary analysis

We conducted a two-sample MR analysis to estimate the causal effects of gut microbiota and circulating metabolites on oral cancer, respectively (Step A1 in Figure 1A and Step B1 in Figure 1B). The primary analysis method was inverse variance weighted (IVW), alongside other analyses such as MR Egger, weighted median, simple mode, and weighted mode (43). Furthermore, we used GSMR, a flexible method that conducts MR analysis with various quasi-independent instruments to assess the causal relationship between a disease and a risk factor using GWAS summary-level data from independent studies (37). The results of the MR analysis were reported as odds ratios (ORs) with their corresponding 95% confidence intervals (CIs). When the *P*-value of IVW was less than 0.05 and the direction of effect observed in both IVW and MR-Egger was concordant, the results were considered statistically significant. A statistically significant two-sided *P*-value, passing the Bonferroni correction *P*-value, was set at 0.0001 (0.05/471) for gut microbiota and 0.0002 (0.05/233) for circulating metabolites. When the significance level (*P* < 0.05) was higher than the Bonferroni-corrected threshold, it was considered suggestive of an association.

#### 2.5.2 Bidirectional causality analysis

To assess the bidirectional causal relationship between gut microbiota, circulating metabolites, and oral cancer, we defined oral cancer as the “exposure” and gut microbiota or circulating metabolites correlated with oral cancer as the “outcomes” (Step A2 in Figure 1A and Step B2 in Figure 1B). The SNPs demonstrating a significant association with oral cancer (*P* < 5 × 10^−5^) were selected as IVs.

#### 2.5.3 Mediation analysis

Based on the two-sample analysis, gut microbiota and circulating metabolites with significant causal effects on oral cancer were included in the mediation analysis. We explored the potential causal effect of the gut microbiota on circulating metabolites (Path a in Figure 1C), and if confirmed, we further used MR analysis to investigate whether circulating metabolites serve as mediators in the pathway linking the gut microbiota to oral cancer.

#### 2.5.4 Sensitivity analysis

Potential horizontal pleiotropy was assessed using MR-PRESSO and MR-Egger regressions. MR-PRESSO detects horizontal pleiotropy outliers through a global test with a *p*-value of less than 0.05, while MR-Egger regression confirms the validity of horizontal pleiotropy at a *p*-value of less than 0.05 (44). In determining heterogeneity, the Cochran Q test is employed, where a *p*-value above 0.05 indicates the absence of heterogeneity (45). And scatter plots were generated to exhibit the connections between SNPs and exposures, as well as between SNPs and outcomes, in order to visualize the results of MR. To evaluate the potential effects of each SNP on the results, a leave-one-out analysis was carried out (46).

In this study, the MR analyses were conducted using the “TwoSampleMR” and “gsmr2” packages in R (v4.2.2) software. For metagenomic data, the Wilcoxon rank-sum test was used to compare the alpha diversity index between groups, while beta diversity was evaluated through principal coordinate analysis (PCoA) and PERMANOVA based on Bray-Curtis distance. Linear discriminant analysis effect size (LEfSe) was used to identify differentially abundant taxa. For targeted metabolomics data, after a normality test, the Student *t*-test or Mann-Whitney U test was used to compare metabolite concentrations, and false discovery rate (FDR) correction was applied for multiple comparisons. Multivariate analysis, including principal component analysis (PCA), was performed to visualize group separation.

## 3 Results

### 3.1 Instrumental variable selection

We identified 9,238 SNPs associated with 471 gut microbial taxa at a level of *P* < 5 × 10^−5^ (Supplementary Table S2). Furthermore, we selected 30,661 SNPs associated with 233 circulating metabolites at a level of *P* < 5 × 10^−5^ (Supplementary Table S3). All of the instrumental variables exhibited F-statistics exceeding 10, with no evidence of weak instrumental variable bias.

### 3.2 Causal effects of gut microbiota and circulating metabolites on oral cancer

**Figure 2:**
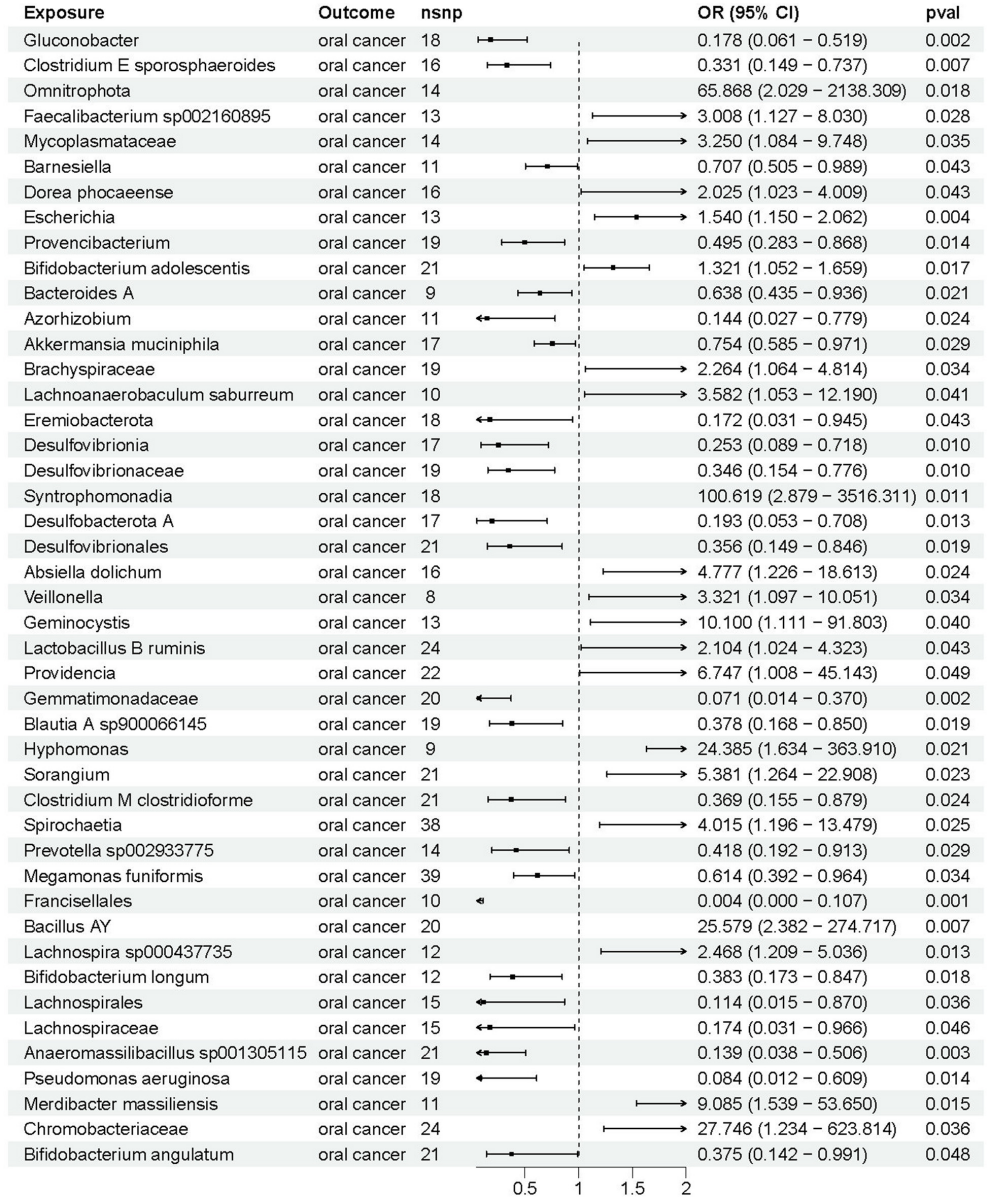
The causal effects between gut microbiota and oral cancer by Mendelian randomization (MR).

The IVW-MR analysis revealed a causal association between 45 gut microbial taxa and the risk of oral cancer (Figure 2). An increased risk of oral cancer was observed in significant relation to the genetic prediction of 21 gut microbial taxa (OR > 1, *P* < 0.05). Moreover, a significant association was found between a reduced risk of oral cancer and the genetic prediction of 24 gut microbial taxa (OR < 1, *P* < 0.05). The results from the five methods of MR analysis can be found in Supplementary Table S4. And detailed information on 775 SNPs for 45 gut microbial taxa is shown in Supplementary Table S5.

**Figure 3:**
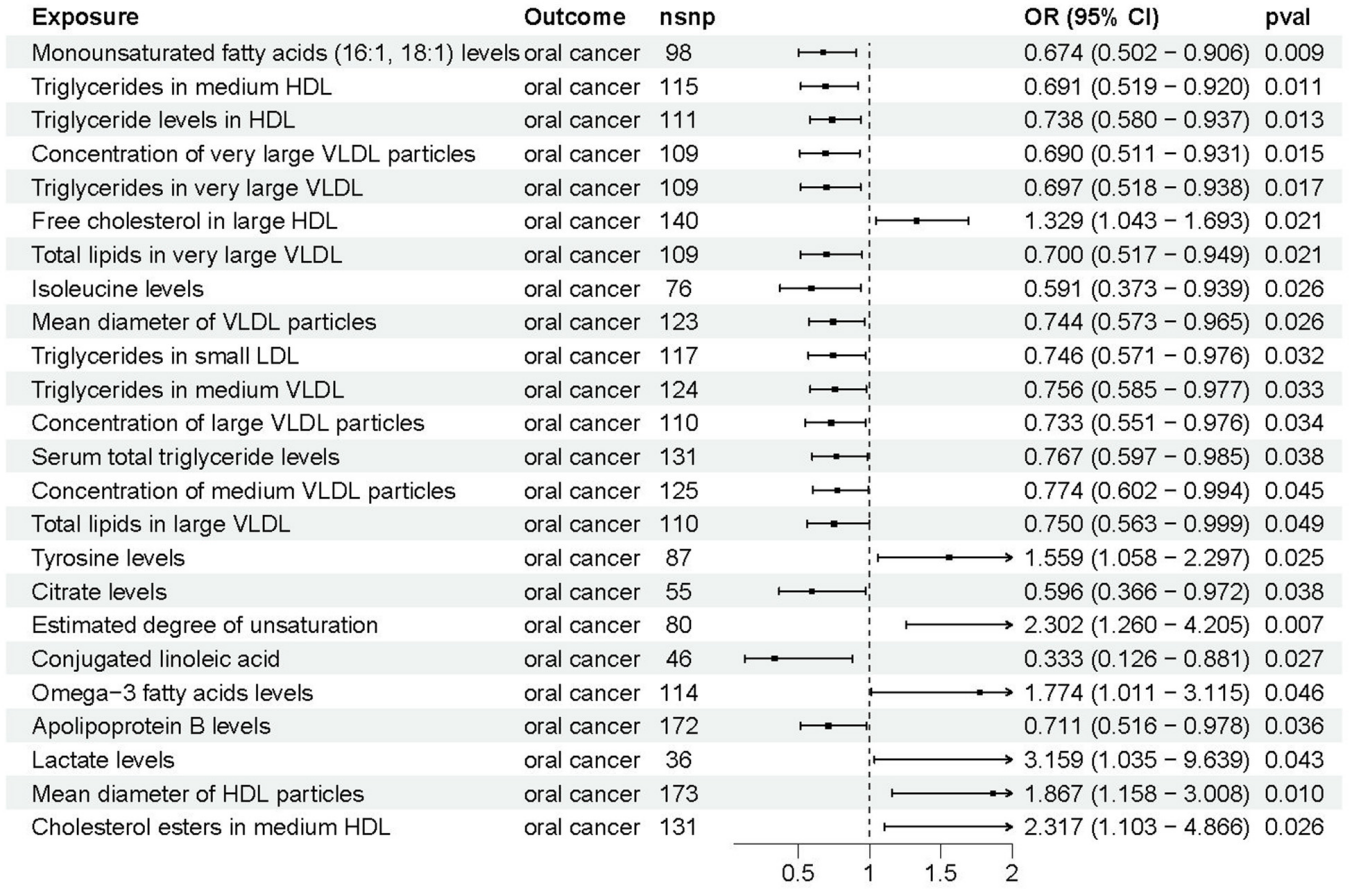
The causal effects between circulating metabolites and oral cancer by Mendelian randomization (MR).

As shown in Figure 3, 24 circulating metabolites were associated with oral cancer, with 7 showing a significant increase in risk and 17 demonstrating a significant decrease in risk for oral cancer. The detailed results of the four additional analysis methods, apart from IVW-MR, are displayed in Supplementary Table S6.

**Figure 4:**
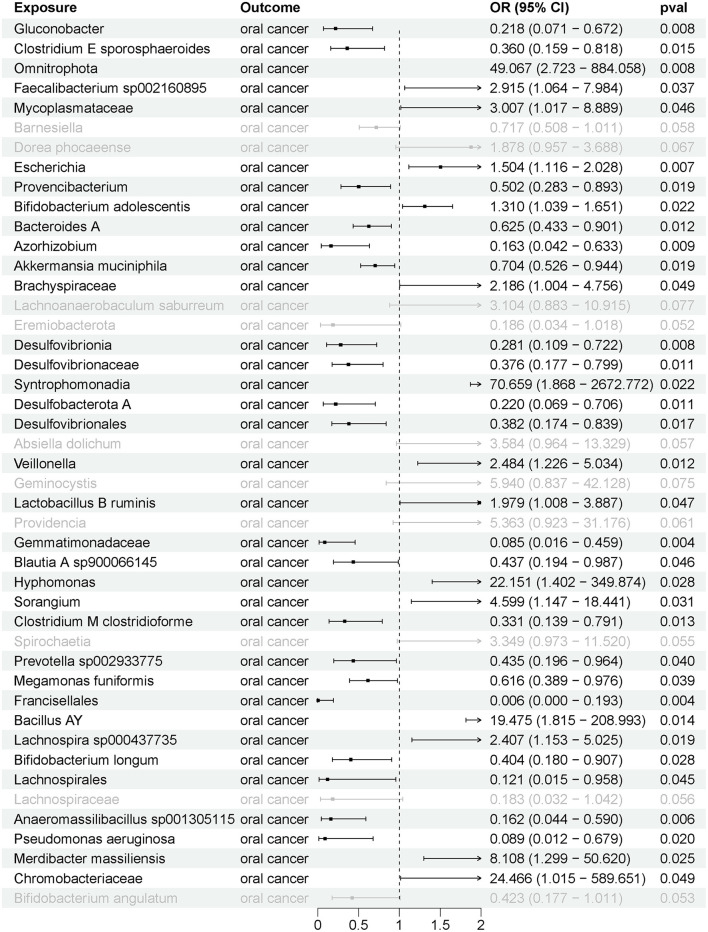
The causal effects between gut microbiota and oral cancer by generalized summary data-based MR (GSMR).

**Figure 5:**
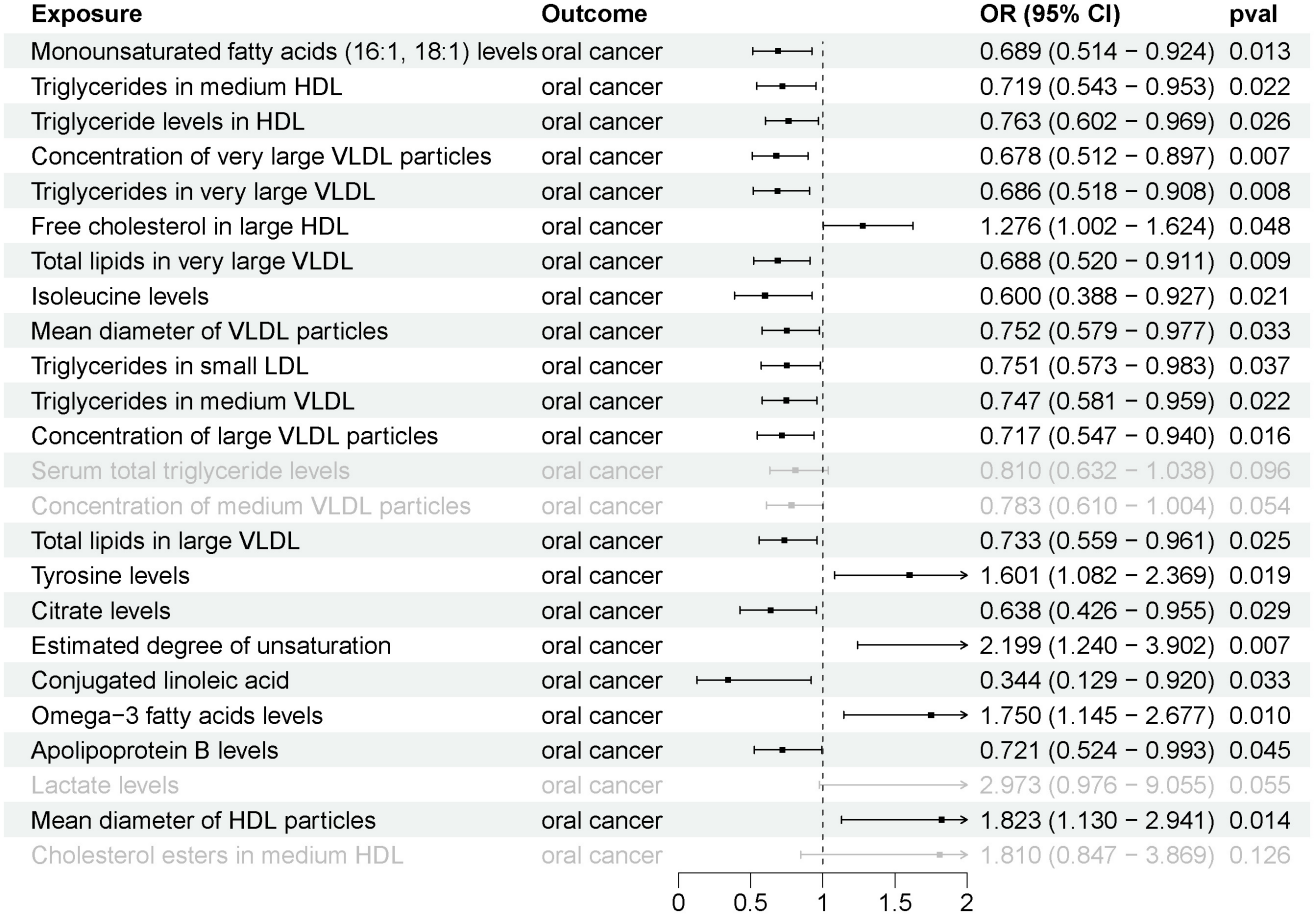
The causal effects between circulating metabolites and oral cancer by generalized summary data-based MR (GSMR).

Further GSMR analysis revealed a correlation between 35 gut microbial taxa and oral cancer (Figure 4), with 15 gut microbial taxa positively associated and 20 negatively associated with oral cancer (Supplementary Table S7). Additionally, 20 circulating metabolites were associated with oral cancer (Figure 5), with 5 showing a positive correlation and 15 showing a negative correlation (Supplementary Table S8).

### 3.3 Sensitivity analyses

The MR-Egger intercept indicated no evidence that genetic pleiotropy biased the results. Furthermore, MR-PRESSO analysis confirmed the absence of horizontal pleiotropy in the MR study (*P* > 0.05, Supplementary Table S9). No significant heterogeneity was observed according to the results of the Cochran's Q test (*P* > 0.05, Supplementary Table S9).

The “leave-one-out” analysis results demonstrated the reliability of MR analysis (Supplementary Figure S1), and the scatter plot indicated the overarching impact of the gut microbiota on oral cancer (Supplementary Figure S2). Additionally, the forest plots revealed the causal relationship between gut microbiota and oral cancer (Supplementary Figure S3).

### 3.4 Reverse causal effects of oral cancer on gut microbiota and circulating metabolites

We performed reverse MR analysis using IVW-MR and GSMR as the primary methods, with oral cancer as the exposure and gut microbiota and circulating metabolites as the outcomes. The results showed no evidence of reverse causal sup1 between gut microbiota, circulating metabolites, and oral cancer (All *P* > 0.05, Supplementary Tables S10–S13).

### 3.5 Mediation analysis

Our study found that both gut microbiota and circulating metabolites had causal effects on oral cancer (IVW-MR and GSMR, *P* < 0.05, Supplementary Tables S4, S6–S8). Furthermore, causal associations were also observed between the gut microbiota associated with oral cancer and the circulating metabolites associated with oral cancer (OR > 1, *P* < 0.05, Supplementary Table S14). The mediation analysis findings suggested that *Akkermansia muciniphila* mitigates the risk of oral cancer through citrate mediation, while *Veillonella* escalates the risk through omega-3 fatty acid mediation.

### 3.6 Clinical validation

#### 3.6.1 The gut microbiota differed between oral cancer patients and healthy controls

**Figure 6:**
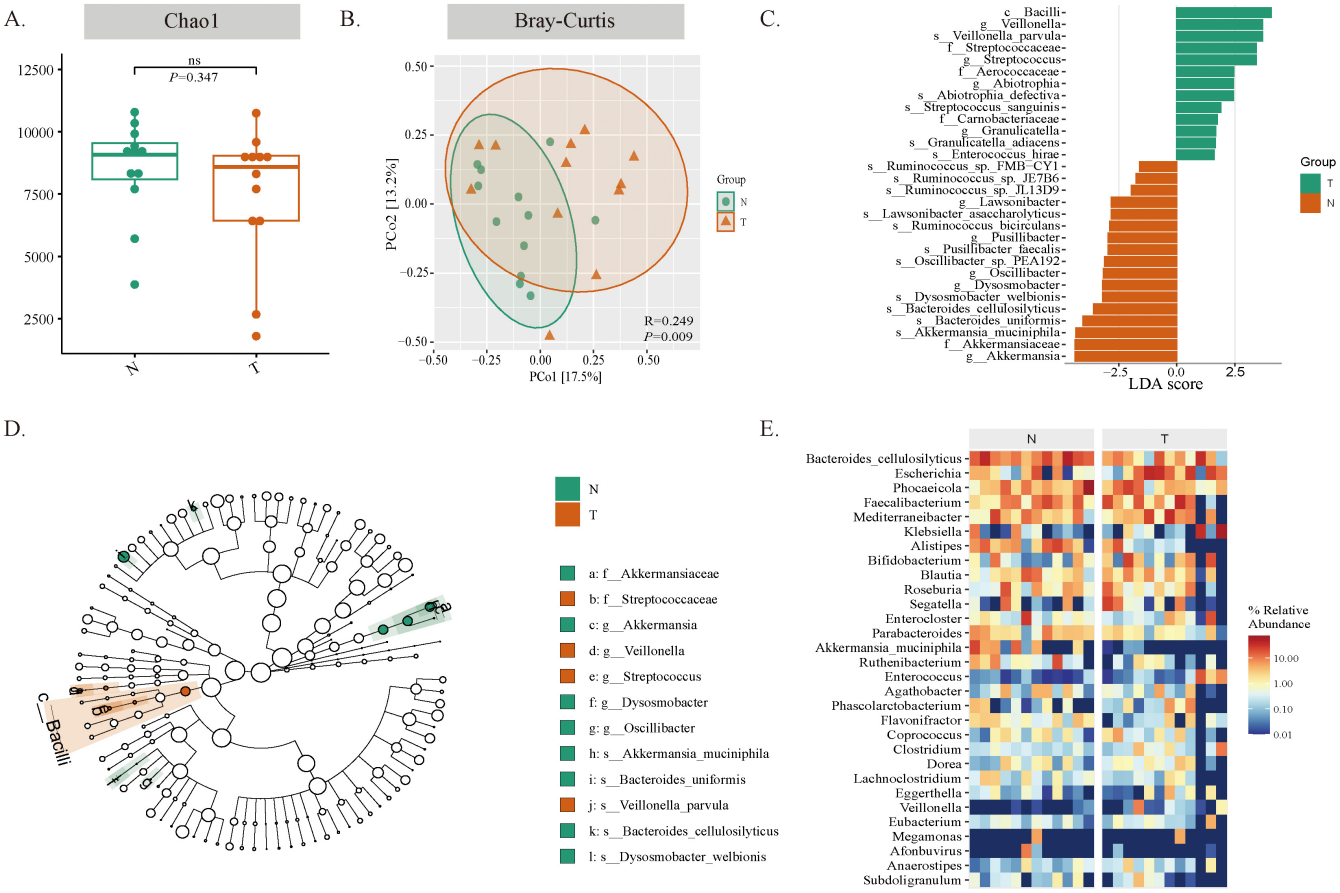
Gut microbiota profiling in oral cancer patients (T) and healthy controls (N). **(A–E)** Metagenomic sequencing analysis was performed on the fecal bacterial DNA from groups T and N. *n* = 12 individuals/group. **(A)** Alpha diversity indices Chao1 show no significant differences between groups (*p* > 0.05). **(B)** PCoA plots based on Bray-Curtis distances reveal distinct clustering between groups (*p* < 0.05). **(C)** LEfSe analysis identifies *Veillonella* as enriched in oral cancer patients and *Akkermansia muciniphila* in healthy controls (LDA score > 3.5). **(D)** Cladogram displays phylogenetic relationships of differentially abundant taxa. **(E)** Heatmap confirms increased *Veillonella* and decreased *Akkermansia muciniphila* abundance in the tumor group.

We performed metagenomic sequencing to characterize the gut microbiota of patients with oral cancer (T) and healthy controls (N). We used alpha diversity metrics, including Chao1, Shannon, ACE, and Fisher alpha, to reflect the richness and evenness of microbial communities within samples. No significant differences were observed between the two groups (Figure 6A and Supplementary Figure S4A, all *P* > 0.05). Subsequently, beta diversity analysis was conducted to measure the heterogeneity of community composition between samples. Principal coordinates analysis (PCoA) based on Bray-Curtis and Jaccard distance metrics showed a clear separation of the gut microbiota between oral cancer patients and healthy controls (Figure 6B and Supplementary Figure S4B, all *P* < 0.05), indicating distinct community structures between the two groups.

Subsequently, we quantified the differences in bacterial communities between groups. The results showed significant differences in microbial community composition between oral cancer patients and healthy controls. To determine the specific bacterial species that contribute to this structural difference, we used Linear Discriminant Analysis Effect Size (LEfSe) for high-dimensional classification comparison. In the oral cancer group, there was a significant increase in the abundance of *Veillonella*. In contrast, the healthy control group was characterized by a higher abundance of *Akkermansia muciniphila* (Figure 6C). The phylogenetic distribution of these differentiating bacteria is shown in the phylogenetic tree (Figure 6D). Furthermore, the relative abundance heat map of these differentiating features further confirmed the increased abundance of *Veillonella* and decreased abundance of *Akkermansia muciniphila* in the tumor group (Figure 6E). In conclusion, these data suggest that there are significant differences in the gut microbial community structure between oral cancer patients and healthy controls.

#### 3.6.2 The serum metabolome differed between oral cancer patients and healthy controls

**Figure 7:**
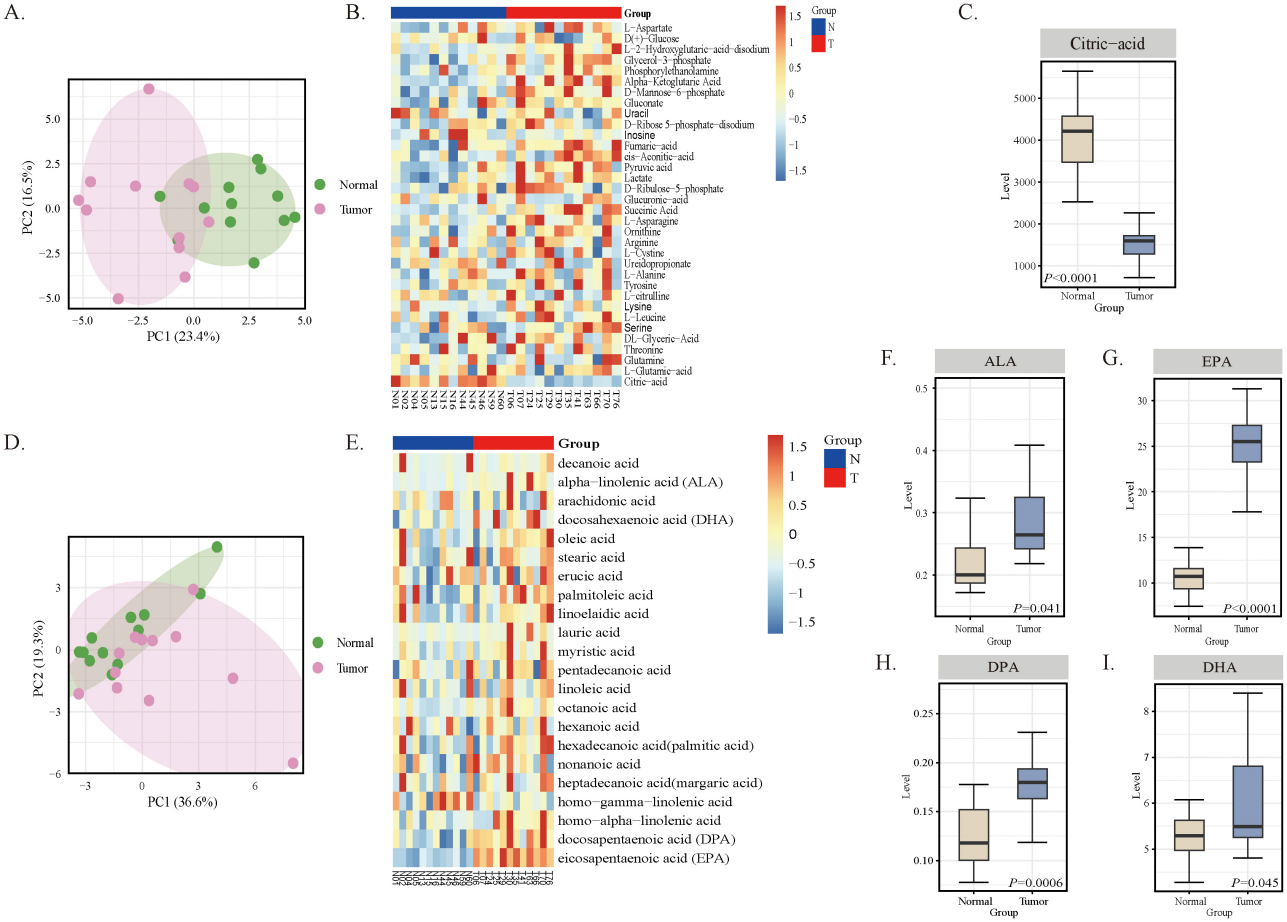
Serum metabolomic profiling in oral cancer patients (T) and healthy controls (N). **(A)** PCA of energy metabolites showing separation between groups. **(B)** Heatmap of differentially abundant energy metabolites, with citrate reduced in the tumor group. **(C)** Quantitative analysis of citrate levels (*p* < 0.05). **(D)** PCA of free fatty acids demonstrating group separation. **(E)** Heatmap of free fatty acid profiles showing elevated omega-3 fatty acids in the tumor group. **(F–I)** Quantitative analysis of ALA, EPA, DPA, and DHA levels (all *p* < 0.05).

Subsequently, we conducted targeted metabolomics analysis based on the results of Mendelian randomization mediation analysis. The targeted metabolomics analysis of energy metabolites revealed significant systemic metabolic alterations in oral cancer patients. Principal component analysis (PCA) clearly differentiated oral cancer patients from healthy controls based on energy metabolism features (Figure 7A). Among the differentially abundant metabolites, citric acid was significantly decreased in the tumor group (Figure 7B). Quantitative analysis confirmed that citric acid levels were significantly lower in oral cancer patients than in healthy controls (Figure 7C). Next, we performed targeted metabolomics analysis of serum free fatty acids. Principal component analysis (PCA) found significant clustering between different patient groups (Figure 7D), indicating systemic changes in lipid metabolism. The metabolic heatmap revealed increased levels of key omega-3 fatty acids, including alpha-linolenic acid (ALA), eicosapentaenoic acid (EPA), docosapentaenoic acid (DPA), and docosahexaenoic acid (DHA) in the tumor group (Figure 7E). Quantitative analysis confirmed that the levels of ALA (Figure 7F), EPA (Figure 7G), DPA (Figure 7H), and DHA (Figure 7I) were significantly higher in oral cancer patients than in healthy controls. These results provide experimental validation for the mediation pathway predicted by MR.

## 4 Discussion

The relationship between microbes and cancer is multifaceted and complex. The host microbiota can increase, decrease, or have no effect on susceptibility to cancer. The assignment of causal roles to particular microbes and microbiotas in cancer, the elucidation of host-microbiota interactions in carcinogenesis, and applying this knowledge for cancer diagnosis and therapy are currently areas of considerable interest (47). In this study, we used summary-level data from 1,974,910 participants in the GWAS Catalog to conduct a bidirectional two-sample MR analysis, aiming to infer potential causal relationships between gut microbiota and oral cancer. Our study provides suggestive evidence that certain gut microbiota are associated with an increased risk of oral cancer, while others may serve as protective factors.

*Akkermansia muciniphila (A. muciniphila)*, a representative member of the gut microbiota, exerts important biological effects on host health. Some studies have indicated that *A. muciniphila* exerts a wide-ranging influence on tumorigenesis, including nasopharyngeal carcinoma (48), prostate cancer (49–51), and lung cancer (52). Additionally, Haberman et al. found significant predictive value of *A. muciniphila* in lung cancer (53). Cascone et al. reported *A. muciniphila* as a potential therapeutic target for lung cancer (54). Our novel findings from MR analysis suggest that the risk of oral cancer may be decreased by *A. muciniphila*, offering crucial insights for future applications of *A. muciniphila* in the prevention and treatment of oral cancer.

*Veillonella* is widely distributed in the natural cavities of the human body, existing as a common anaerobic bacterium in the oral cavity, throat, respiratory tract, and gastrointestinal tract (55). Oosterlinck et al. discovered that *Veillonella* was more abundant in gastric tumor samples characterized by high MUC13 expression (56). Recent studies have revealed that the presence of *Veillonella* in the lower airway may serve as an indicator of lung cancer progression (57). Our MR analysis provides suggestive evidence that *Veillonella* is associated with an increased risk of oral cancer, thus presenting valuable clues for early diagnosis of oral cancer.

Previous studies have reported associations between gut microbiota and various cancers. However, substantial inconsistencies remain across studies in microbiota composition, direction of effects, and statistical significance (58, 59). Although one study reported an enrichment of *Veillonella* in gastric cancer (56), its positive association with oral cancer has not yet been validated in independent large-scale cohorts. In addition, most existing studies are cross-sectional and involve limited sample sizes, making it difficult to exclude reverse causation and confounding bias (60, 61). Although the present study partially addresses these limitations through an MR design, further validation in large-scale prospective cohort studies is still needed.

This study characterized the effects of gut microbiota on oral cancer as either beneficial or harmful based on differences in relative abundance. However, the specific mechanisms underlying the association between gut microbiota and oral cancer remain unclear. We hypothesized that circulating metabolites act as mediators in the relationship between gut microbiota and oral cancer. According to the MR analysis, citrate was significantly associated with a reduced risk of oral cancer (OR = 0.596, 95% CI = 0.366–0.972, *P* = 0.038). Regarding risk factors for oral cancer, our findings are consistent with a recent study (62), which reported that omega-3 fatty acids were associated with an increased risk of oral cancer (OR = 1.774, 95% CI = 1.011–3.115, *P* = 0.046). Cortes et al. revealed that dysbiosis in the gut microbiota can affect the metabolic pathways of key metabolites, such as tryptophan, citrate, and omega-3 fatty acids (63). Our mediation analysis also provided genetic evidence supporting a mediating role of circulating metabolites in the association between gut microbiota and oral cancer.

In this study, bidirectional two-sample MR analyses were conducted using GWAS summary data characterized by large sample sizes and robust statistical power. The application of GSMR enabled the identification of instrumental outliers and the removal of pleiotropic SNPs. Additionally, the findings of this study were based on an exploration of causal associations at the genetic level. Multiple MR analyses were employed for causal inference and result validation, ensuring the robustness of the findings and imperviousness to the effects of confounding and horizontal pleiotropy. It is noteworthy that although the GWAS summary data used in this study were derived from large-scale, well-standardized cohorts (41, 42), heterogeneity may still exist across different original studies in fecal sample collection, DNA extraction, 16S rRNA vs. metagenomic sequencing, and taxonomic annotation algorithms. These methodological differences could introduce bias in estimating the relative abundance of specific bacterial taxa, thereby affecting instrumental variable selection in MR analyses. Future studies should promote standardization in gut microbiota research and prioritize metagenomic sequencing to minimize amplification bias. Therefore, caution is warranted when interpreting the results of MR analyses, and future efforts should focus on harmonizing methodologies across microbiome and metabolome GWAS studies to improve comparability and reproducibility.

Another important issue to consider when interpreting microbiome findings in oral cancer is the distinction between surface-associated oral microbiota and tissue-associated microbiota. Microbial communities detected in saliva, oral rinses, or plaque samples may primarily reflect the overall oral ecological environment and host-related factors, such as oral hygiene, smoking, alcohol consumption, and periodontal status. In contrast, tissue-associated microbiota obtained from biopsy specimens may provide more direct insight into local host-microbe interactions within the tumor microenvironment, including microbial colonization of the lesion itself and its potential contribution to carcinogenesis (64). Therefore, microbial findings derived from surface samples and tissue samples should not be regarded as interchangeable. This distinction may also partly explain the inconsistencies reported across studies of oral potentially malignant disorders and oral squamous cell carcinoma. In the present study, our primary focus was on gut microbiota and circulating metabolites, and the clinical validation cohort was based on fecal metagenomic sequencing and serum metabolomics rather than oral tissue microbiome profiling. Future studies integrating gut microbiota, oral surface microbiota, and tissue-associated microbiota may provide a more comprehensive understanding of the spatially distinct microbial contributions to oral cancer.

Within this context, our clinical multi-omics data provide complementary biological support for the observed associations. In patients with oral cancer, we observed a gut microbial signature characterized by an enrichment of *Veillonella* and a depletion of *Akkermansia muciniphila*, along with a distinct serum metabolomic profile marked by reduced citrate levels and elevated omega-3 fatty acid levels. Although clinical findings do not imply causation, the convergence of genetic predictions from MR and simultaneous changes in patient fecal samples and blood significantly bolsters the biological plausibility of a gut microbiota-metabolite-oral cancer axis. From a translational perspective, our findings highlight several potential clinical directions worth exploring. First, the circulating metabolites identified as mediators, citrate and omega-3 fatty acids, may serve as candidate biomarkers for oral cancer risk stratification if validated in prospective cohorts. Second, the association between gut microbiota and oral cancer raises the possibility of microbiome-targeted interventions. One study found that *Akkermansia muciniphila* mitigated carboxymethylcellulose-induced exacerbation of acute pancreatitis (65), suggesting a potentially feasible direction for future investigation. Conversely, reducing the abundance of *Veillonella* through dietary modulation or prebiotics might lower the risk, although this remains speculative. Third, the mediation analysis suggests that modifying the levels of specific circulating metabolites, either directly or through gut microbiota modulation, could represent a potential therapeutic strategy.

While our study offers novel insights, several limitations should be considered when interpreting the results. Firstly, we used a *p*-value threshold of *P* < 1 × 10^−5^ to screen IVs and acknowledged that while these IVs may not have a strong correlation, they nevertheless provide a more thorough evaluation of the relationship between gut microbiota and oral cancer. Secondly, although we performed multiple sensitivity analyses to assess the assumptions of the MR study, residual confounding and horizontal pleiotropy could not be completely excluded. Thirdly, our clinical validation component was limited by a small sample size from a single hospital, which may have reduced the statistical power and generalizability of the multi-omics findings. In addition, it should be recognized that MR analysis is a hypothesis-driven method, and further experimental and mechanistic studies are needed to clarify the underlying biological mechanisms.

Moreover, our findings provide genetic evidence supporting a causal role of specific gut microbiota and circulating metabolites in oral cancer. However, they may not imply the degree of clinical applicability, such as aiding in diagnosis, risk stratification, or prognosis. Prospective cohort studies and randomized controlled trials are necessary to assess the efficacy and safety of any microbiome-modulating interventions before clinical applicability.

## 5 Conclusion

This study provides a comprehensive exploration of causal relationships among gut microbiota, circulating metabolites, and oral cancer using IVW-MR and GSMR. No reverse causal effects of oral cancer on gut microbiota or circulating metabolites were observed. Mediation analysis further identified two gut microbial taxa that may influence the risk of oral cancer through specific circulating metabolites. These findings provide genetic evidence supporting causal inference, but remain largely exploratory. They suggest several directions for future investigation, including prospective cohort studies and randomized trials of microbiome-targeted interventions. Such efforts may help inform strategies for oral cancer prevention and management, and potentially contribute to earlier diagnosis and therapeutic development.

## Data Availability

The Mendelian randomization analysis data used in this study were sourced from the GWAS Catalog (https://www.ebi.ac.uk/gwas/). The metagenomic sequencing data presented in this study are deposited in the NCBI Sequence Read Archive (SRA) under BioProject accession number PRJNA1461404.

## Supplementary material

Supplementary Figure S1 MR leave-one-out sensitivity analysis for Gut microbiota on oral cancer. The ‘'exposure‘' markers in the figures, from (1) to (45), are labeled as *Gluconobacter, Clostridium E sporosphaeroides, Omnitrophota, Faecalibacterium sp002160895, Mycoplasmataceae, Barnesiella, Dorea phocaeense, Escherichia, Provencibacterium, Bifidobacterium adolescentis, Bacteroides A, Azorhizobium, Akkermansia muciniphila B, Brachyspiraceae, Lachnoanaerobaculum saburreum, Eremiobacterota, Desulfovibrionia, Desulfovibrionaceae, Syntrophomonadia, Desulfobacterota A, Desulfovibrionales, Absiella dolichum, Veillonella, Geminocystis, Lactobacillus B ruminis, Providencia, Gemmatimonadaceae, Blautia A sp900066145, Hyphomonas, Sorangium, Clostridium M clostridioforme, Spirochaetia, Prevotella sp002933775, Megamonas funiformis, Francisellales, Bacillus AY, Lachnospira sp000437735, Bifidobacterium longum, Lachnospirales, Lachnospiraceae, Anaeromassilibacillus sp001305115, Pseudomonas aeruginosa, Merdibacter massiliensis, Chromobacteriaceae*, and *Bifidobacterium angulatum*, while the ‘'outcome‘' markers are consistently represented as oral cancer.

Supplementary Figure S2 Scatter plots for the effect of Gut microbiota on oral cancer. The ‘'exposure‘' markers in the figures, from (1) to (45), are labeled as *Gluconobacter, Clostridium E sporosphaeroides, Omnitrophota, Faecalibacterium sp002160895, Mycoplasmataceae, Barnesiella, Dorea phocaeense, Escherichia, Provencibacterium, Bifidobacterium adolescentis, Bacteroides A, Azorhizobium, Akkermansia muciniphila B, Brachyspiraceae, Lachnoanaerobaculum saburreum, Eremiobacterota, Desulfovibrionia, Desulfovibrionaceae, Syntrophomonadia, Desulfobacterota A, Desulfovibrionales, Absiella dolichum, Veillonella, Geminocystis, Lactobacillus B ruminis, Providencia, Gemmatimonadaceae, Blautia A sp900066145, Hyphomonas, Sorangium, Clostridium M clostridioforme, Spirochaetia, Prevotella sp002933775, Megamonas funiformis, Francisellales, Bacillus AY, Lachnospira sp000437735, Bifidobacterium longum, Lachnospirales, Lachnospiraceae, Anaeromassilibacillus sp001305115, Pseudomonas aeruginosa, Merdibacter massiliensis, Chromobacteriaceae*, and *Bifidobacterium angulatum*, while the ‘'outcome‘' markers are consistently represented as oral cancer.

Supplementary Figure S3 Forest plots for the effect of Gut microbiota on oral cancer. The ‘'exposure‘' markers in the figures, from (1) to (45), are labeled as *Gluconobacter, Clostridium E sporosphaeroides, Omnitrophota, Faecalibacterium sp002160895, Mycoplasmataceae, Barnesiella, Dorea phocaeense, Escherichia, Provencibacterium, Bifidobacterium adolescentis, Bacteroides A, Azorhizobium, Akkermansia muciniphila B, Brachyspiraceae, Lachnoanaerobaculum saburreum, Eremiobacterota, Desulfovibrionia, Desulfovibrionaceae, Syntrophomonadia, Desulfobacterota A, Desulfovibrionales, Absiella dolichum, Veillonella, Geminocystis, Lactobacillus B ruminis, Providencia, Gemmatimonadaceae, Blautia A sp900066145, Hyphomonas, Sorangium, Clostridium M clostridioforme, Spirochaetia, Prevotella sp002933775, Megamonas funiformis, Francisellales, Bacillus AY, Lachnospira sp000437735, Bifidobacterium longum, Lachnospirales, Lachnospiraceae, Anaeromassilibacillus sp001305115, Pseudomonas aeruginosa, Merdibacter massiliensis, Chromobacteriaceae*, and *Bifidobacterium angulatum*, while the ‘'outcome‘' markers are consistently represented as oral cancer.

Supplementary Figure S4 Alpha-diversity and beta-diversity in oral cancer patients (T) and healthy controls (N). **(A)** Alpha-diversity (based on Shannon, ACE, and Fisher alpha). **(B)** PCoA was conducted to assess beta diversity based on the Jaccard metric distance.
