# Supplementary figures and images for: Insights into the effects of gut microbiota and circulating metabolites on oral cancer: Mendelian randomization analysis and clinical validation
Source: Front Nutr. 2026 May 21;13:1827331. doi: 10.3389/fnut.2026.1827331 (PMC13233540; doi:10.3389/fnut.2026.1827331)

Figure. S1

(1) (2)


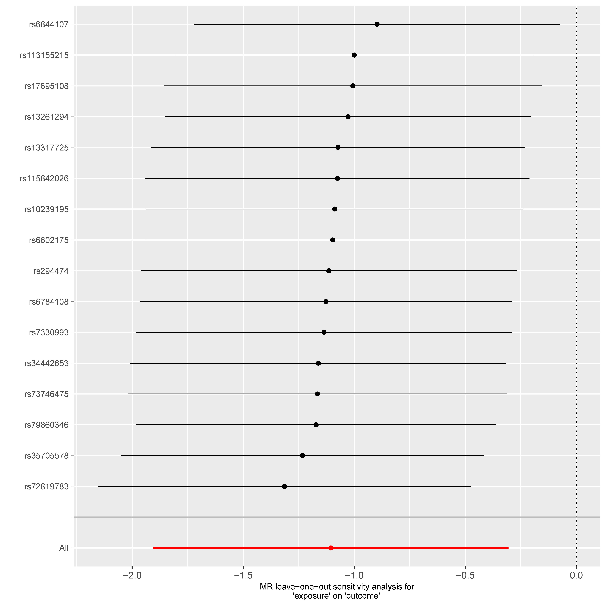

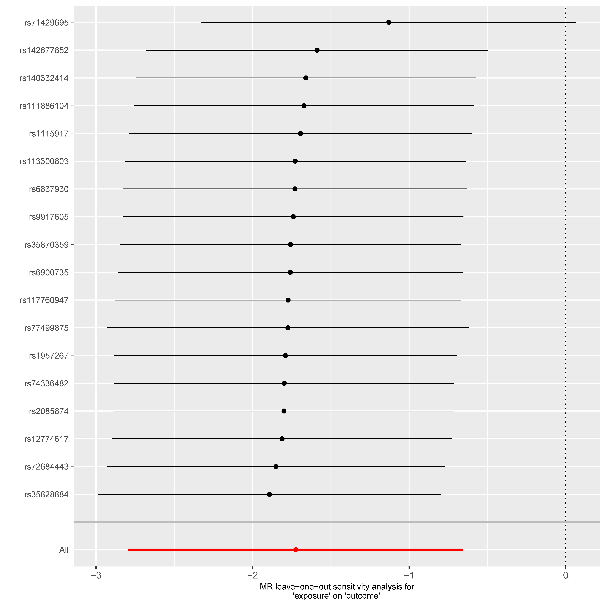


(3) (4)


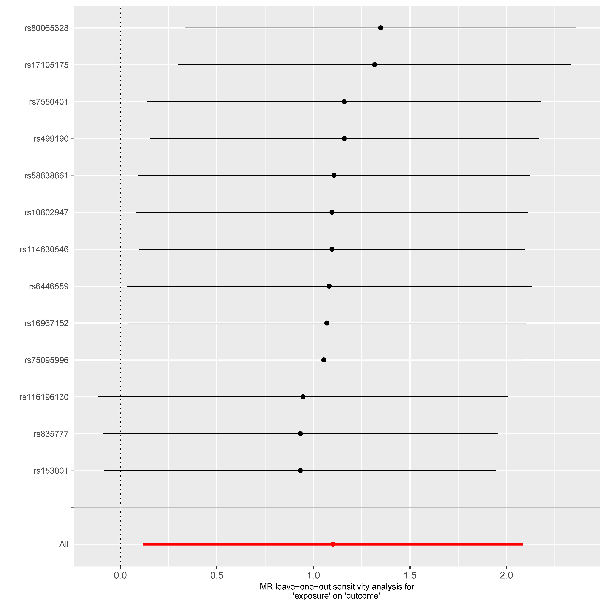

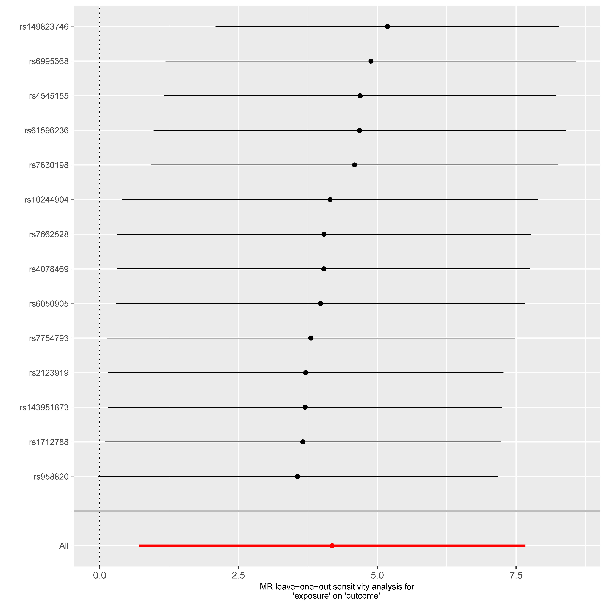


(5) (6)


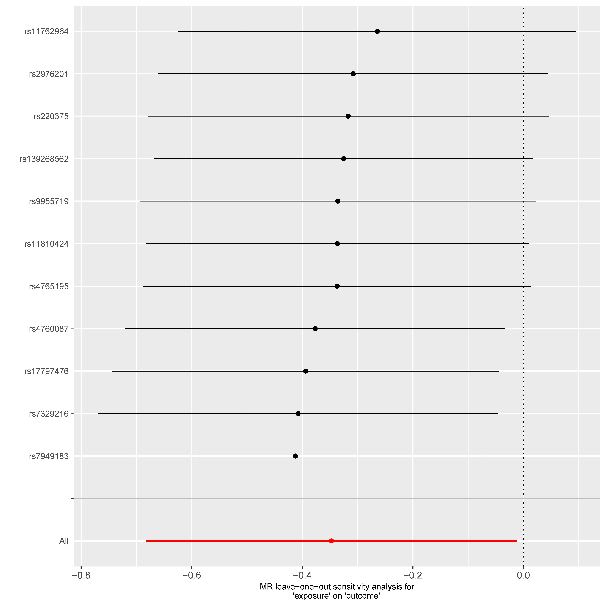

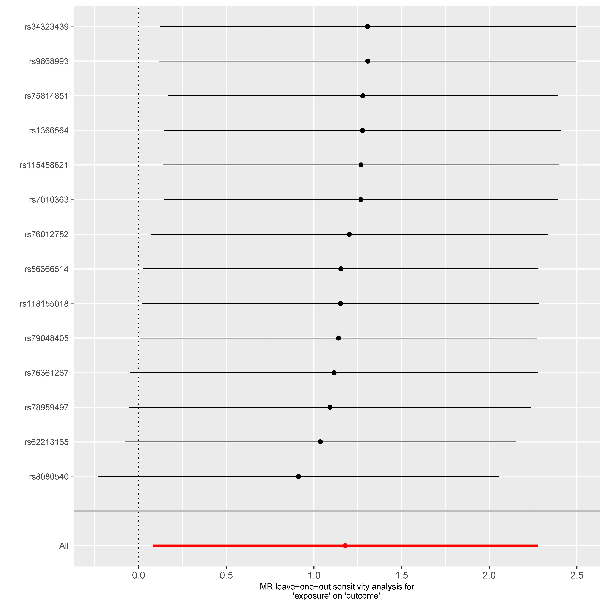


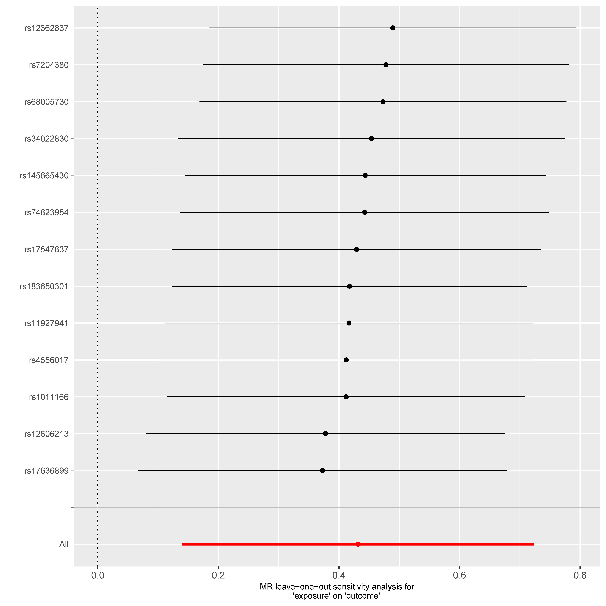

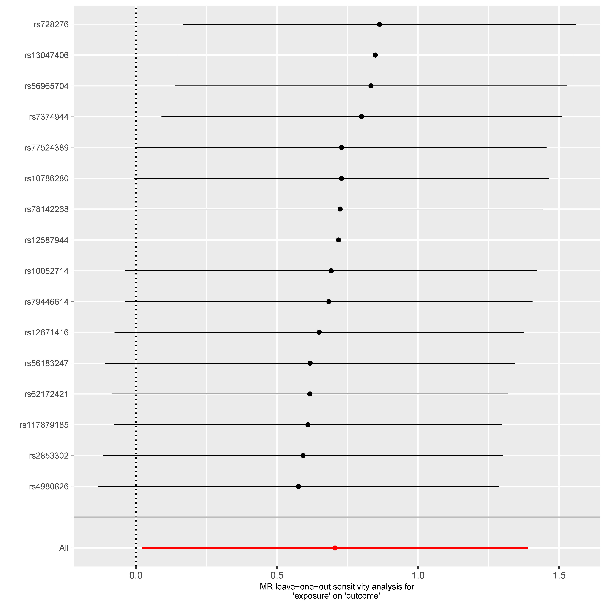
(7) (8)


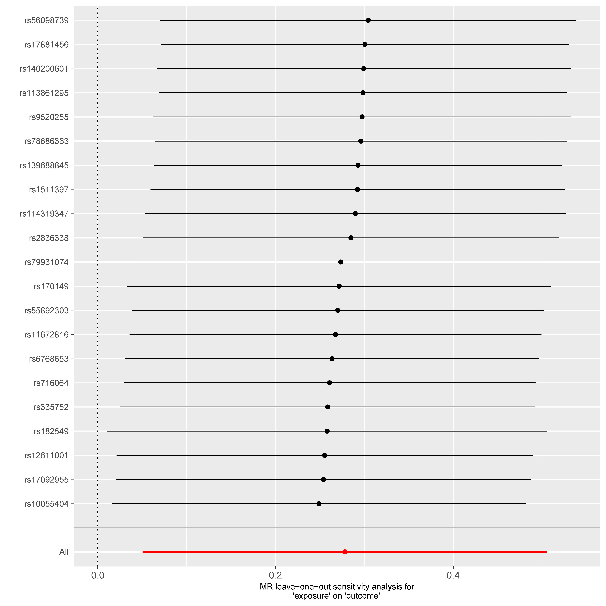
(9) (10)


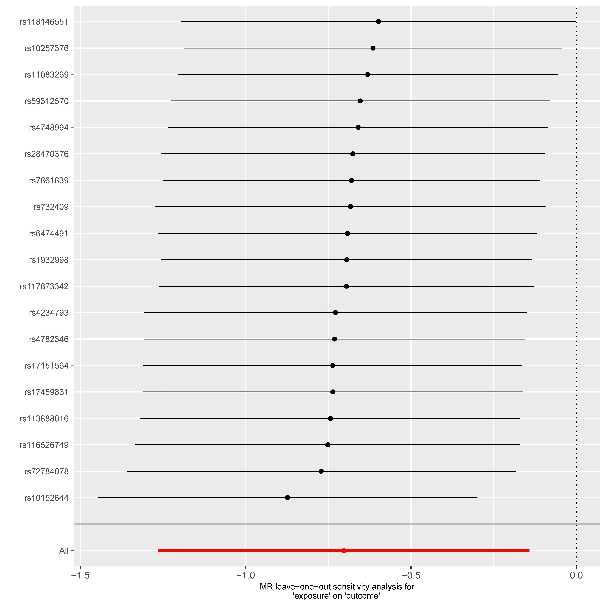


(11) (12)


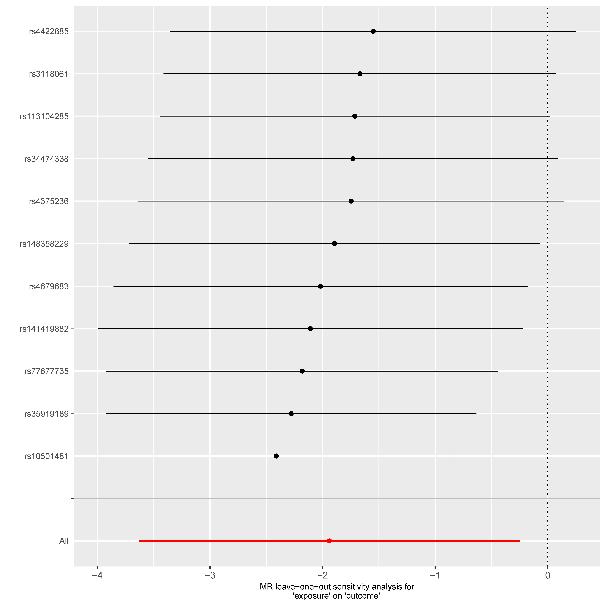

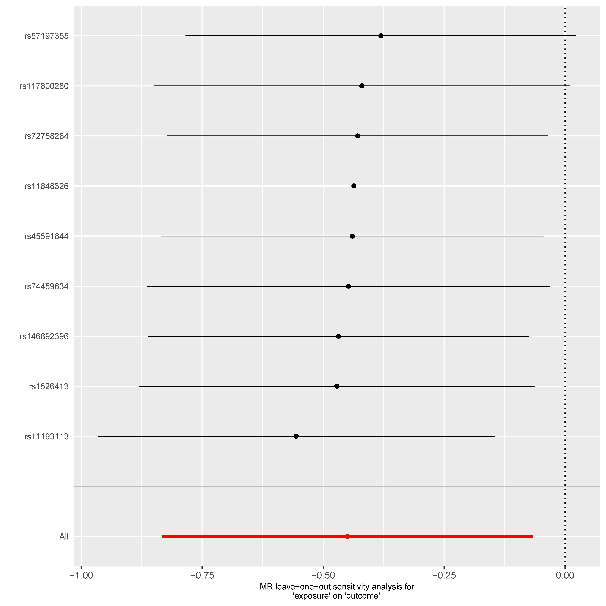


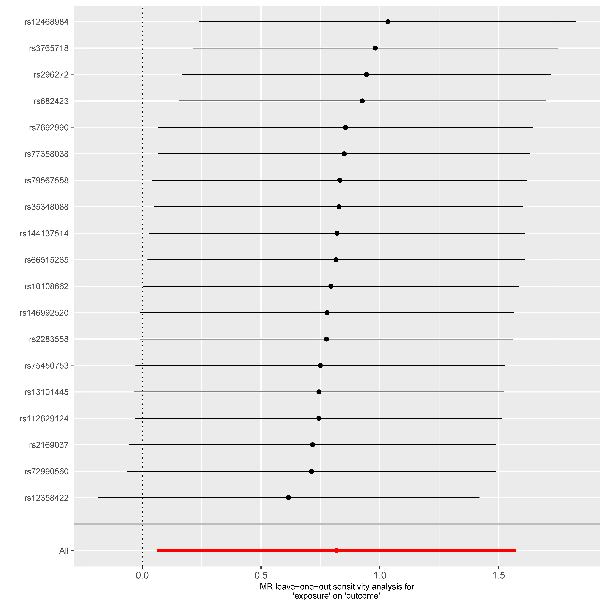
(13) (14
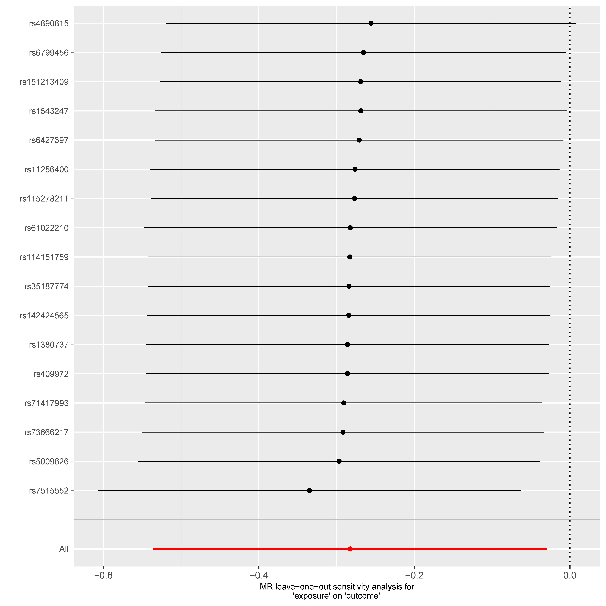
)


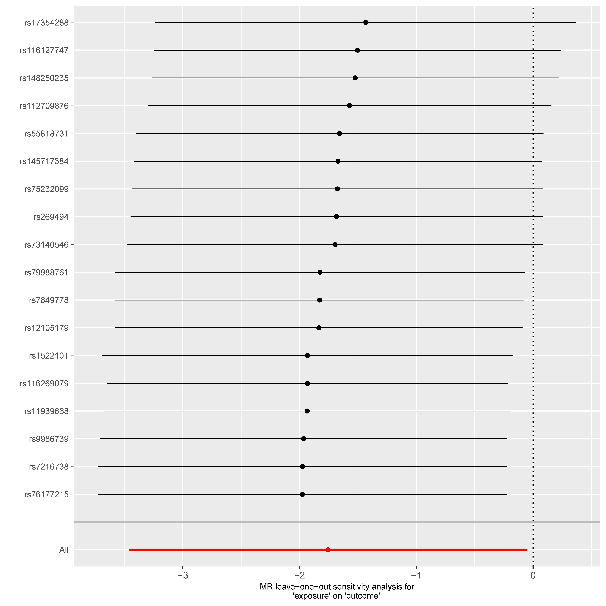
(15) (16
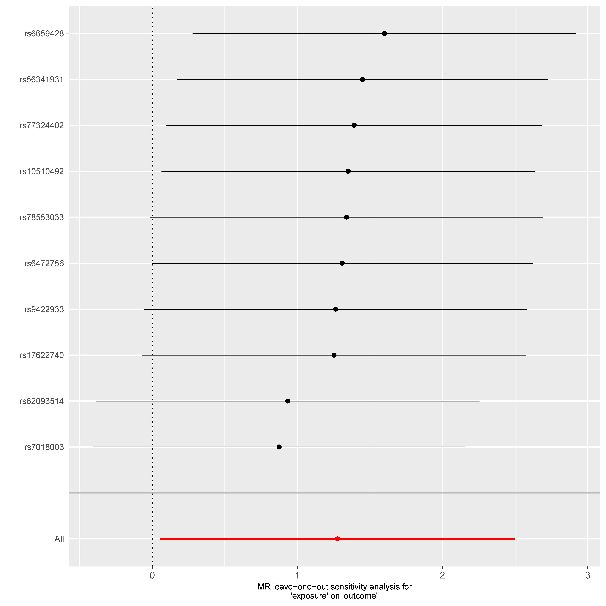
)


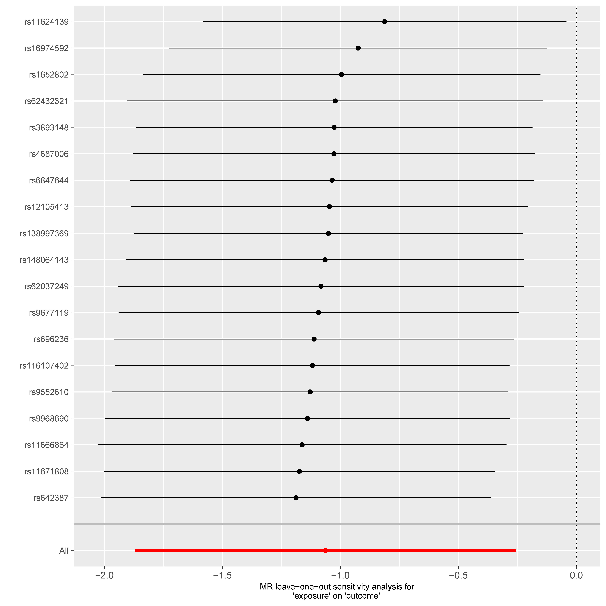
(17) (18)


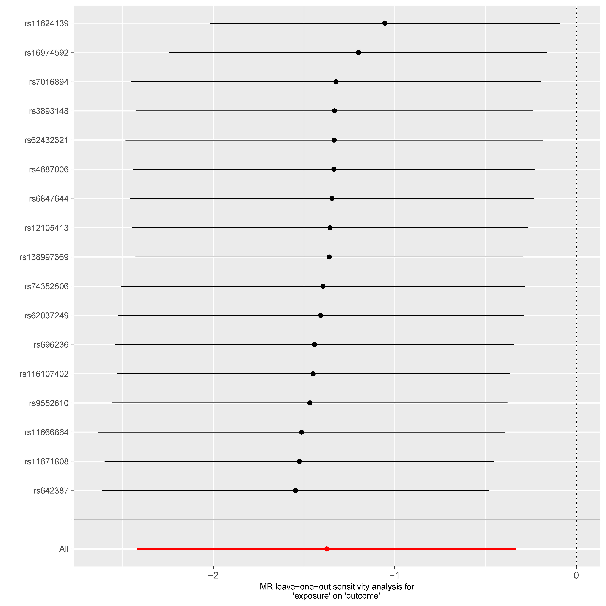


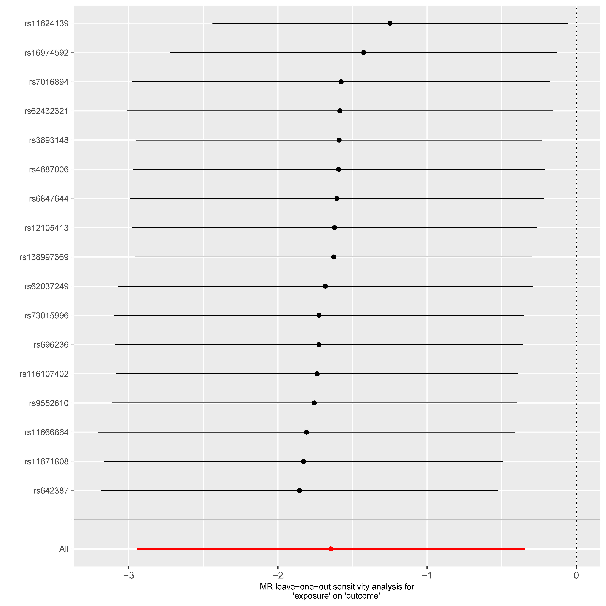
(19) (20
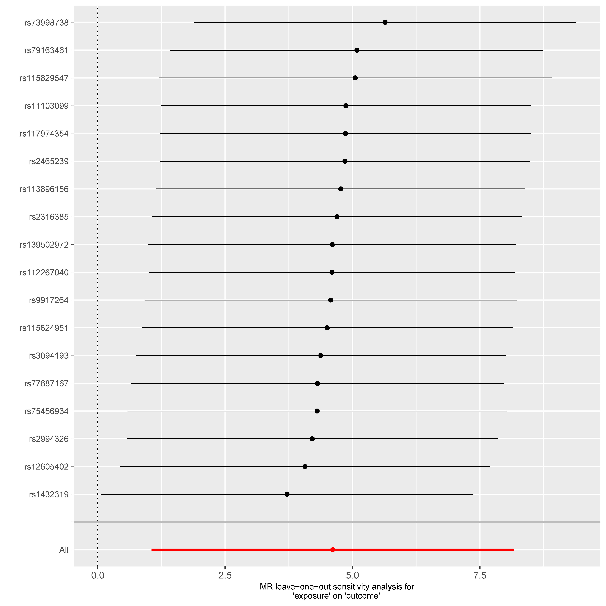
)


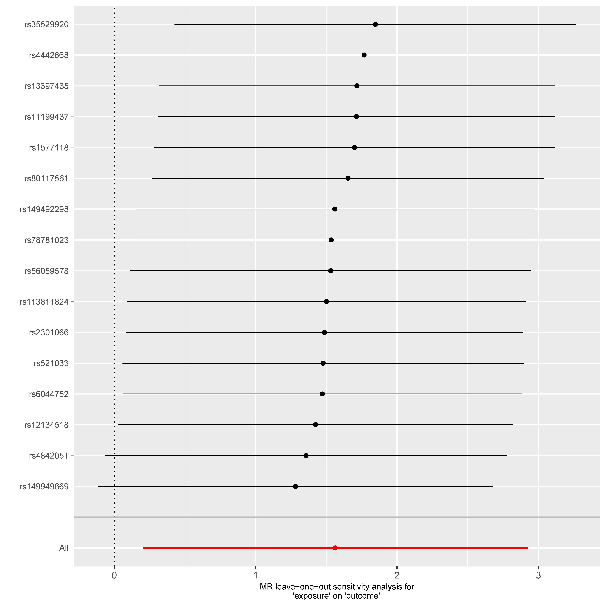
(21) (22)


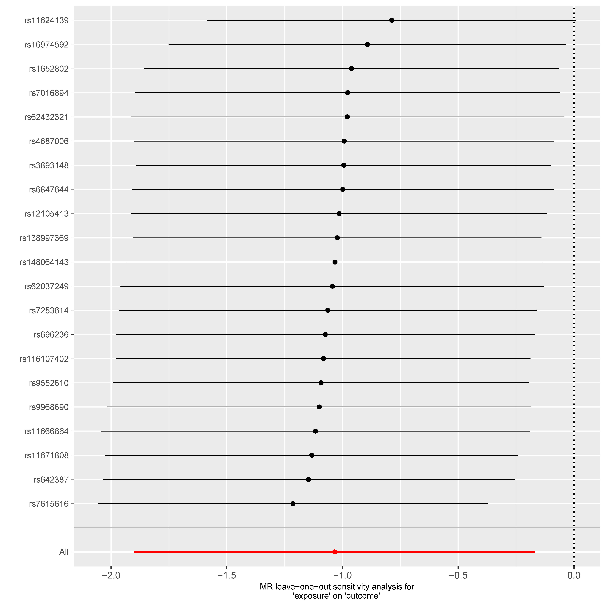


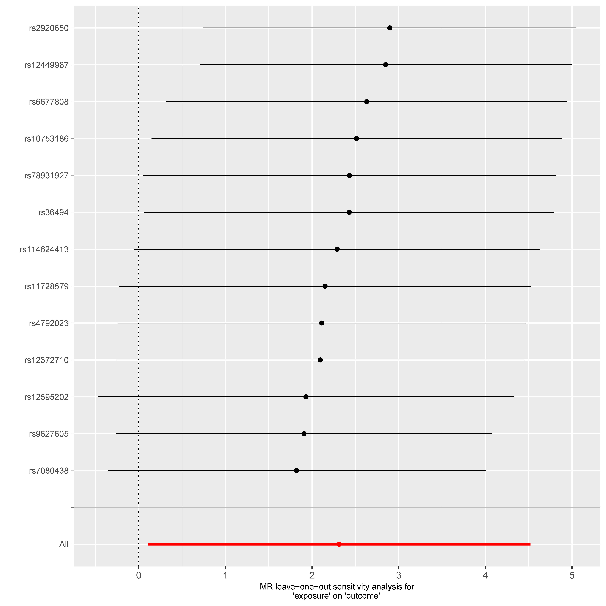
(23) (24)


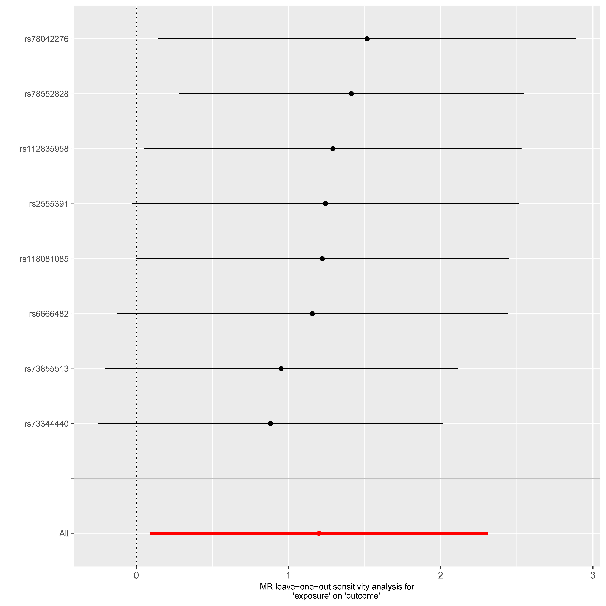


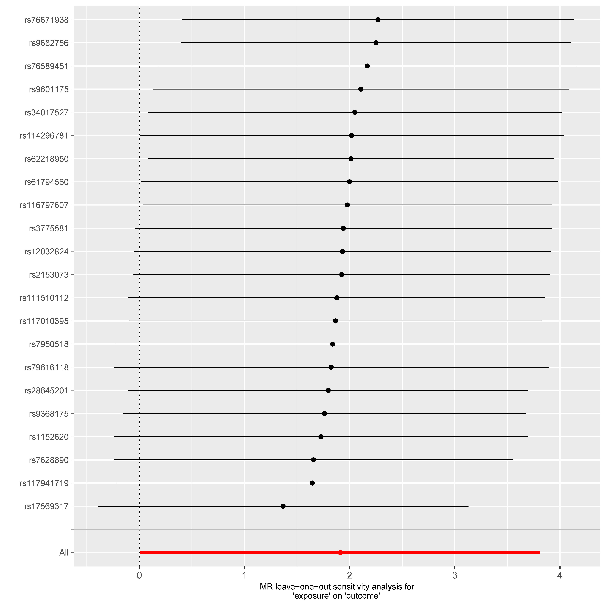
(25) (26
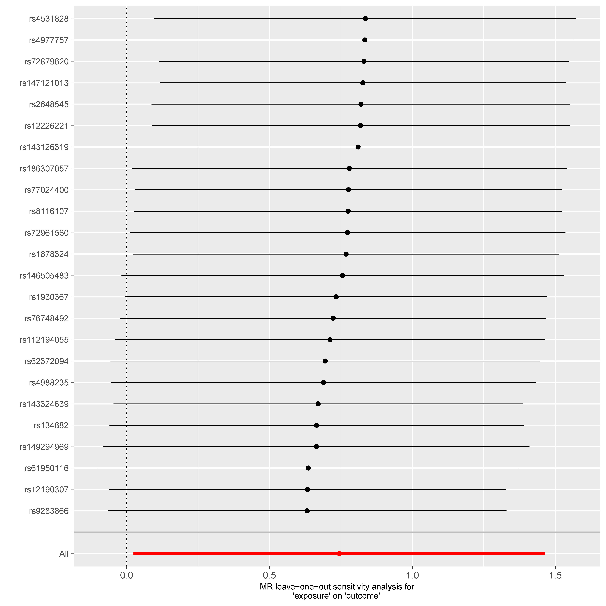
)


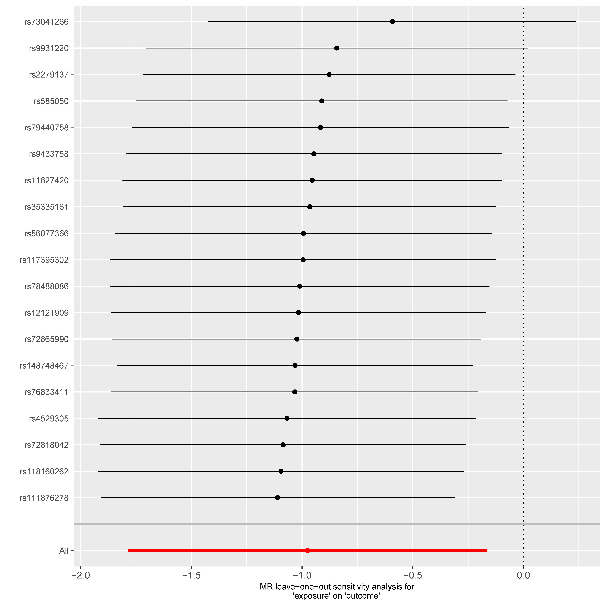
(27) (28)


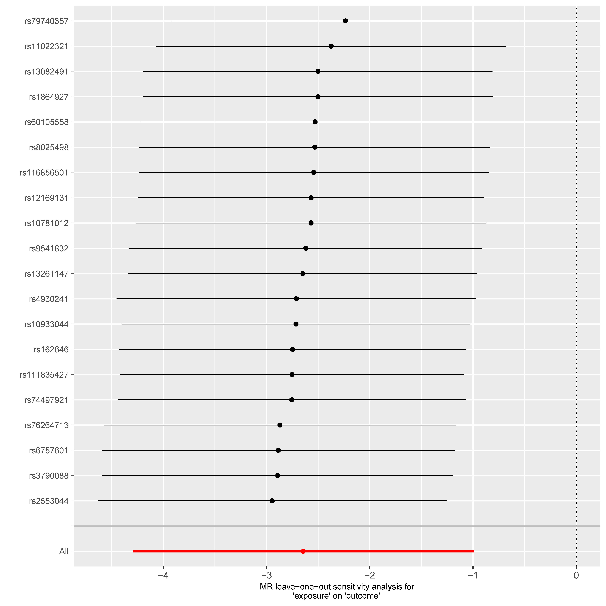


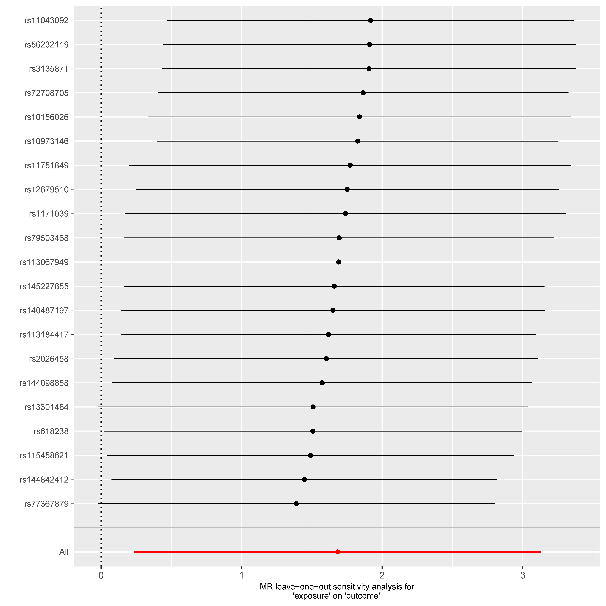
(29) (30)


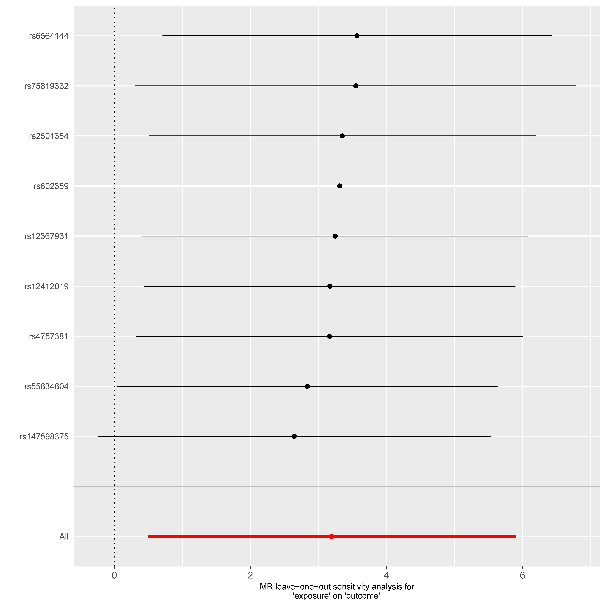


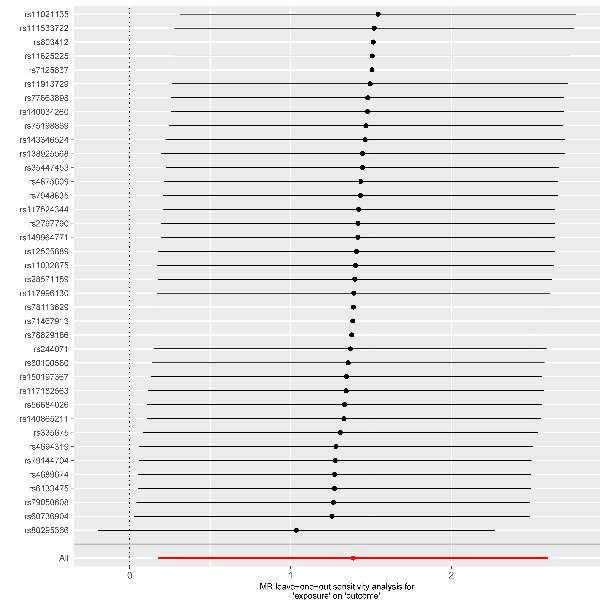
(31) (32
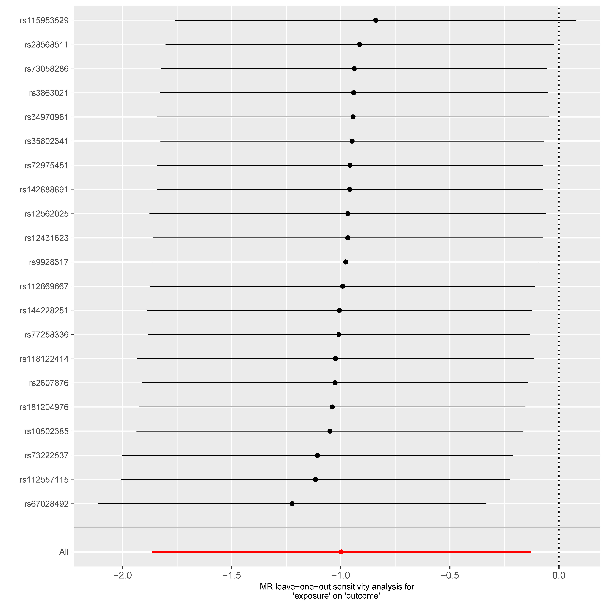
)


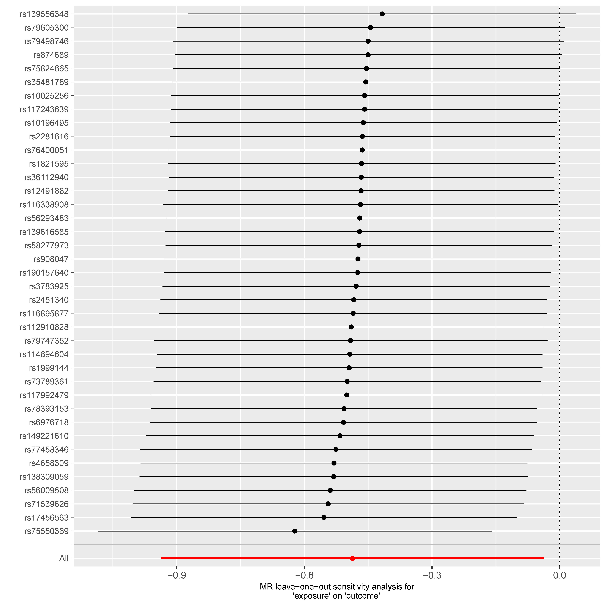
(33) (34)
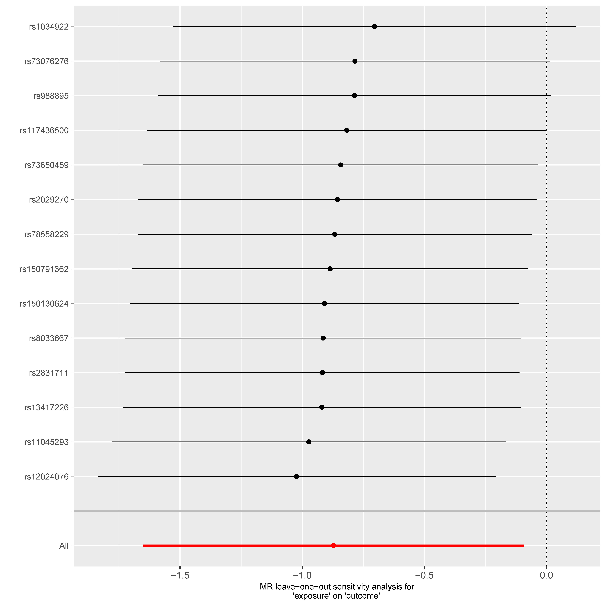


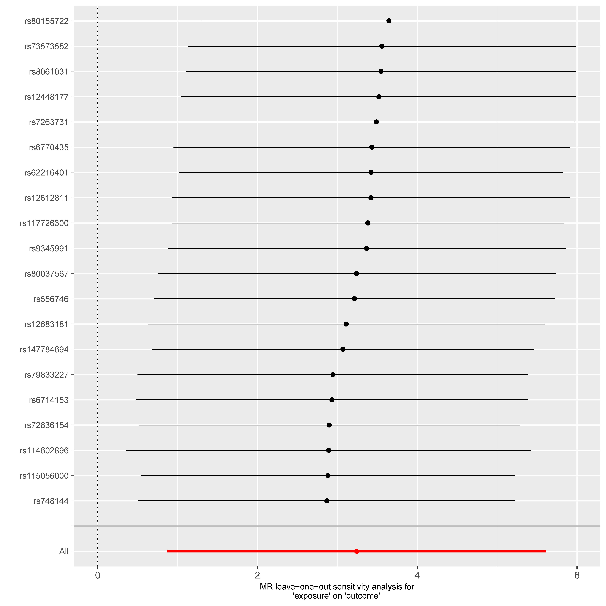
(35) (36)


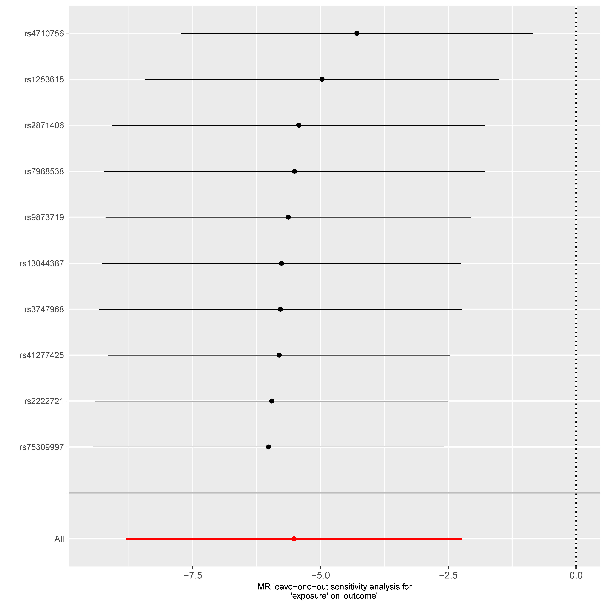


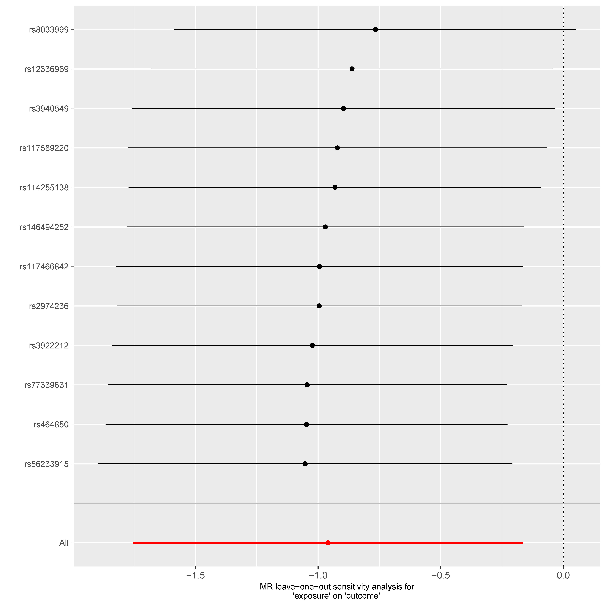
(37) (38)
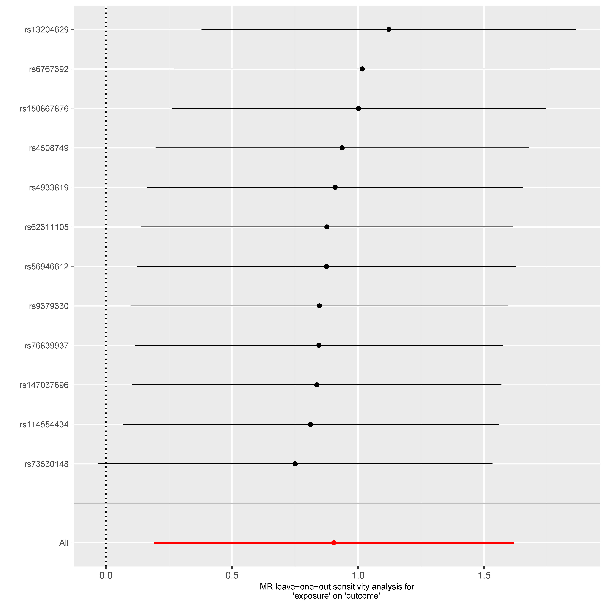


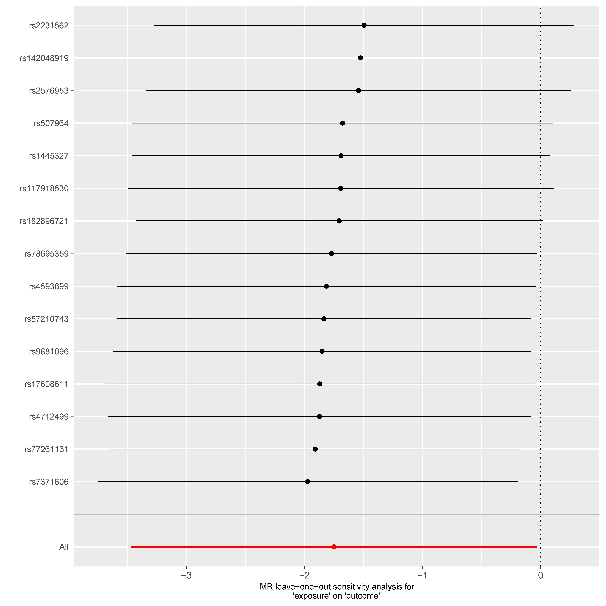
(39) (40
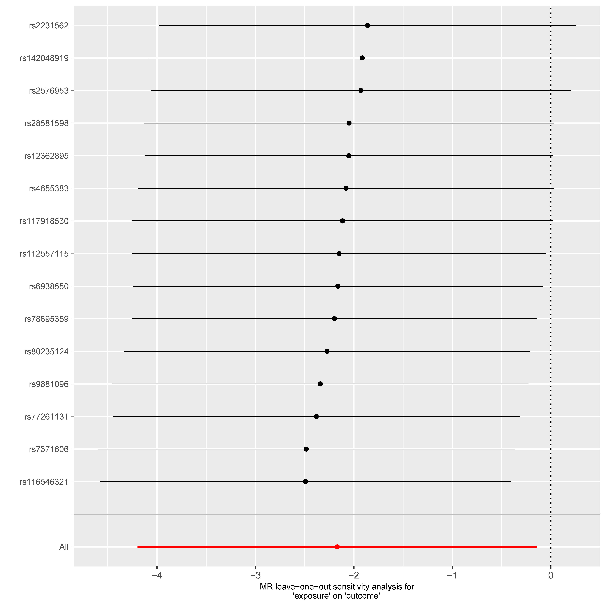
)


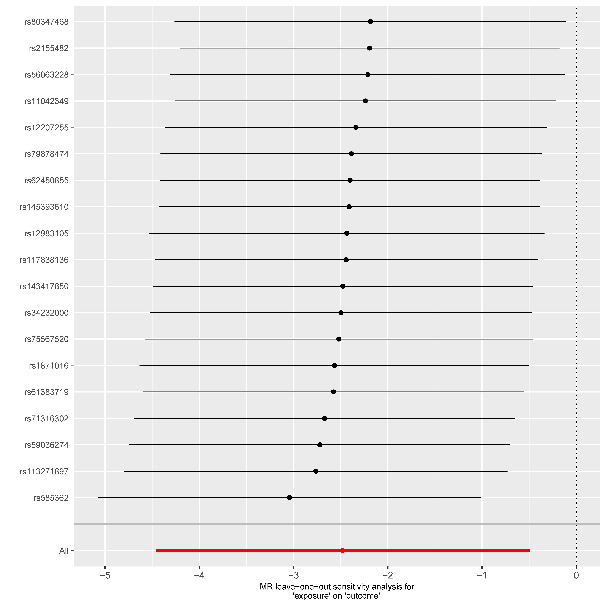
(41) (42)


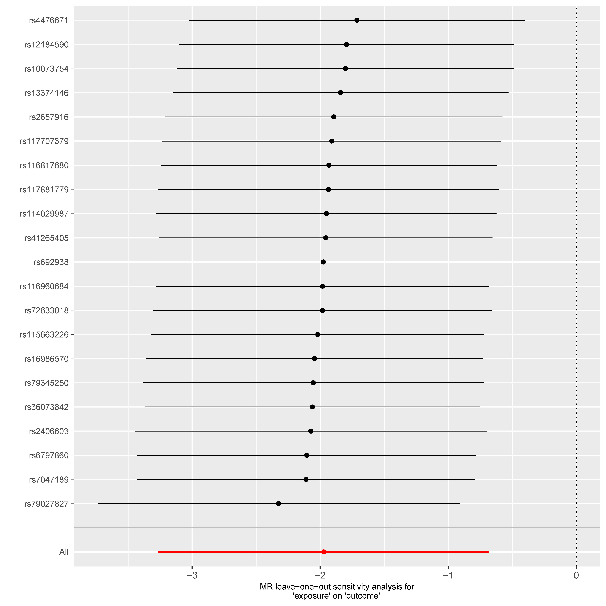


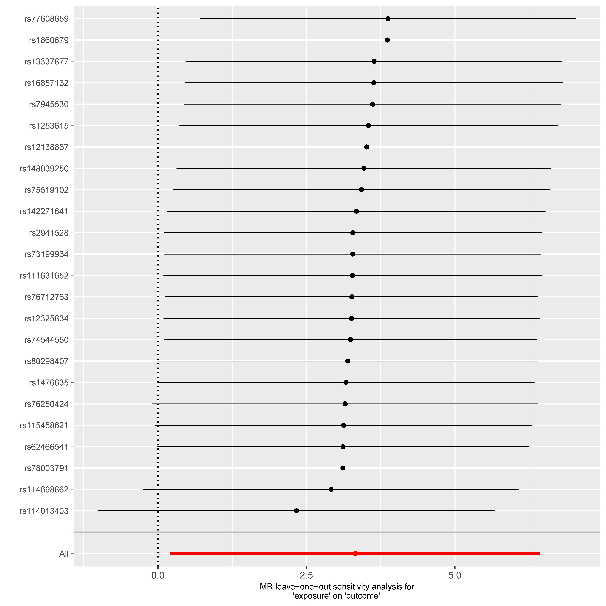
(43) (44
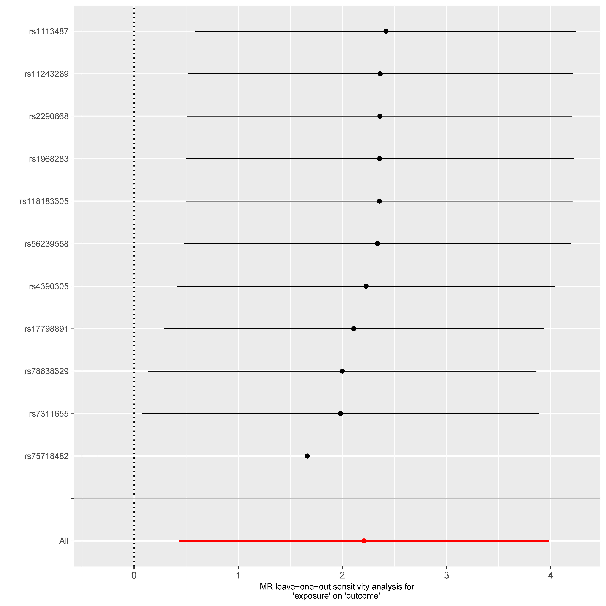
)

(45
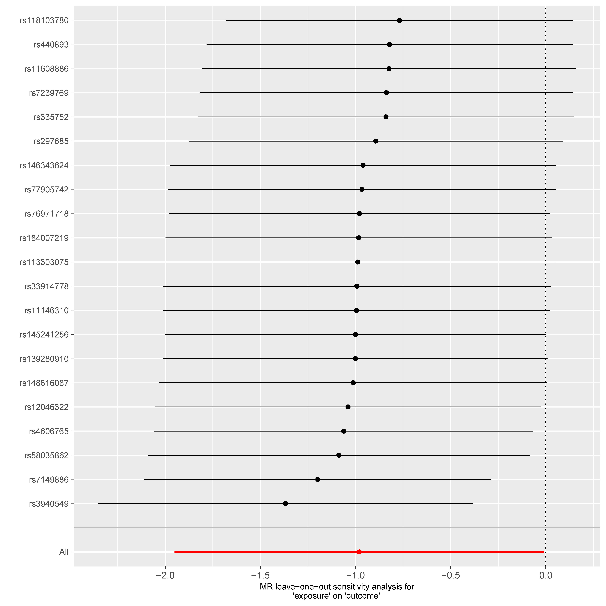
)

Supplement: Supplementary Figure S1 — MR leave-one-out sensitivity analysis for Gut microbiota on oral cancer. The ‘'exposure‘' markers in the figures, from (1) to (45), are labeled as Gluconobacter, Clostridium E sporosphaeroides, Omnitrophota, Faecalibacterium sp002160895, Mycoplasmataceae, Barnesiella, Dorea phocaeense, Escherichia, Provencibacterium, Bifidobacterium adolescentis, Bacteroides A, Azorhizobium, Akkermansia muciniphila B, Brachyspiraceae, Lachnoanaerobaculum saburreum, Eremiobacterota, Desulfovibrionia, Desulfovibrionaceae, Syntrophomonadia, Desulfobacterota A, Desulfovibrionales, Absiella dolichum, Veillonella, Geminocystis, Lactobacillus B ruminis, Providencia, Gemmatimonadaceae, Blautia A sp900066145, Hyphomonas, Sorangium, Clostridium M clostridioforme, Spirochaetia, Prevotella sp002933775, Megamonas funiformis, Francisellales, Bacillus AY, Lachnospira sp000437735, Bifidobacterium longum, Lachnospirales, Lachnospiraceae, Anaeromassilibacillus sp001305115, Pseudomonas aeruginosa, Merdibacter massiliensis, Chromobacteriaceae, and Bifidobacterium angulatum, while the ‘'outcome‘' markers are consistently represented as oral cancer. [file Data_Sheet_1.docx]

Figure. S2

(1) (2)


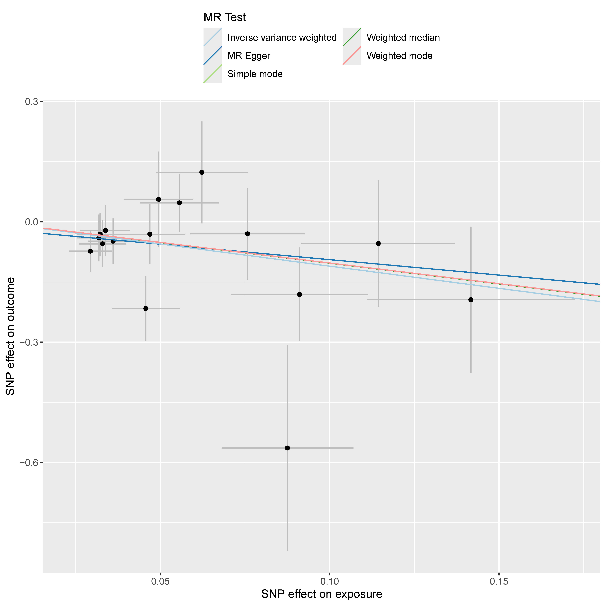

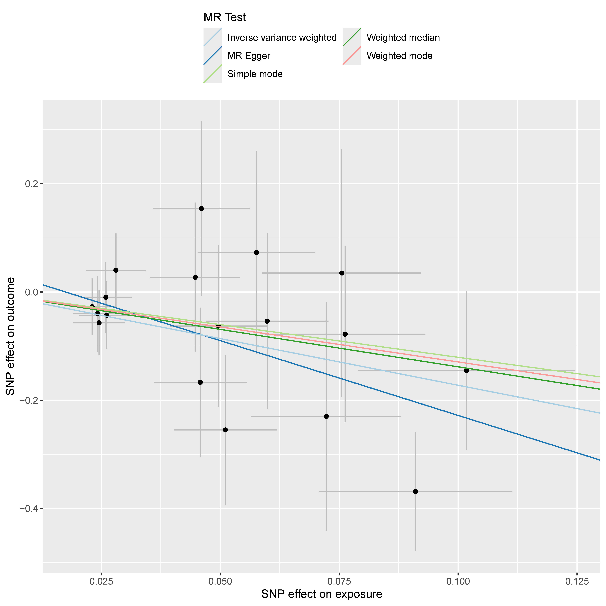


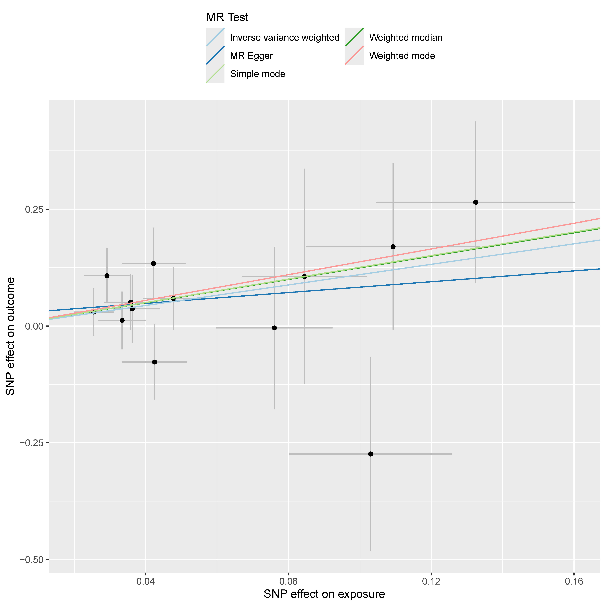
(3) (4
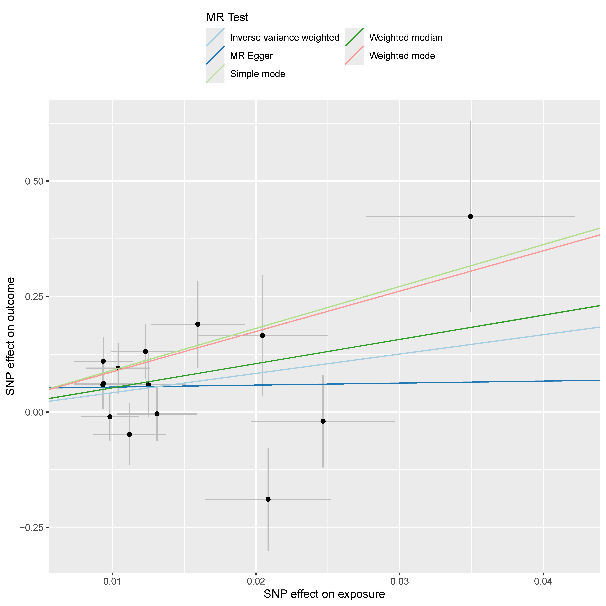
)

(5) (6)


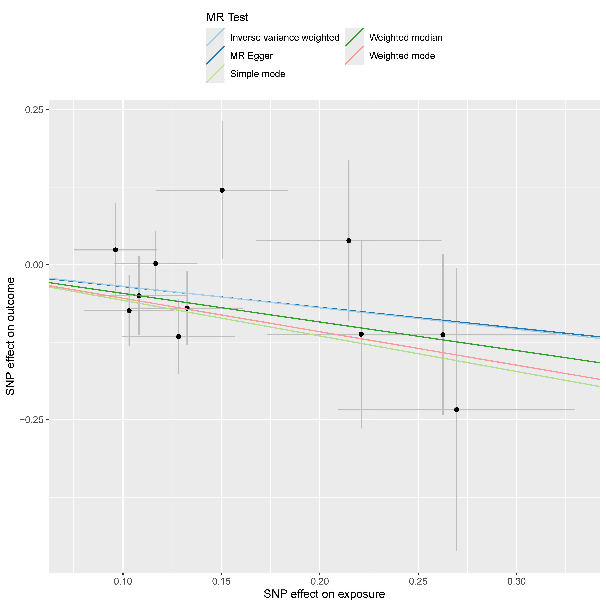

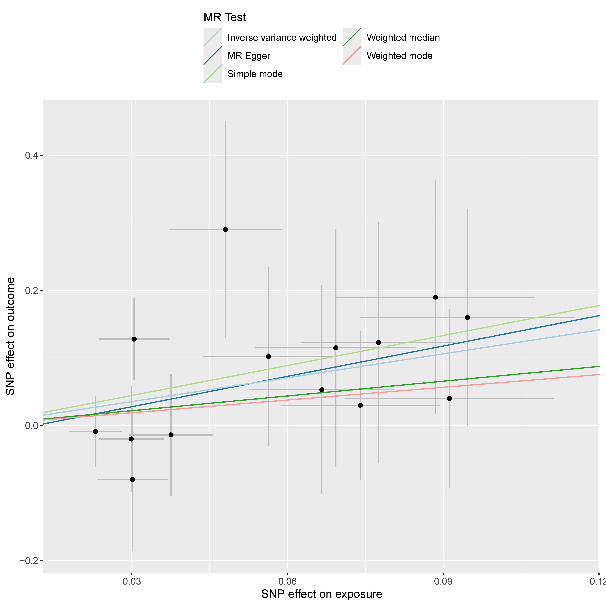


(7) (8)


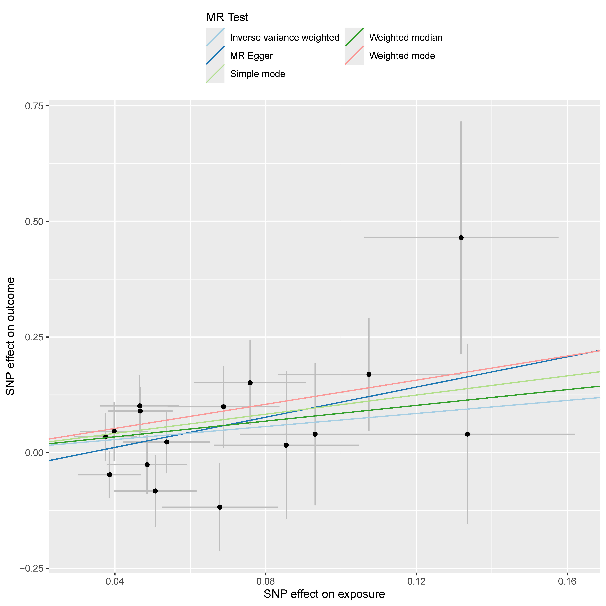

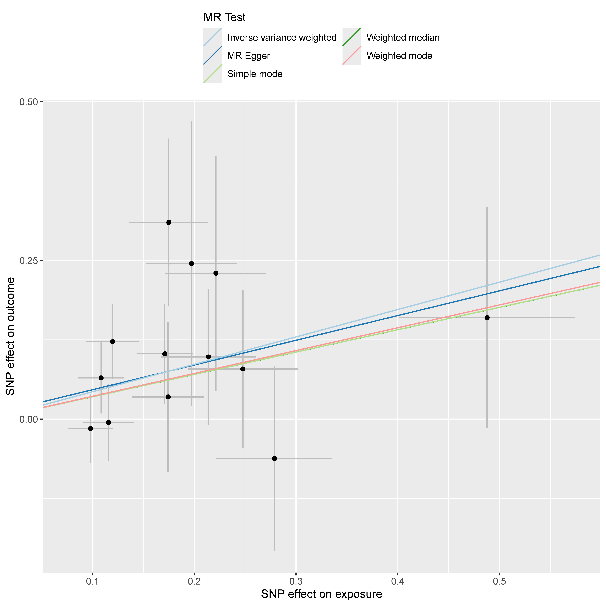


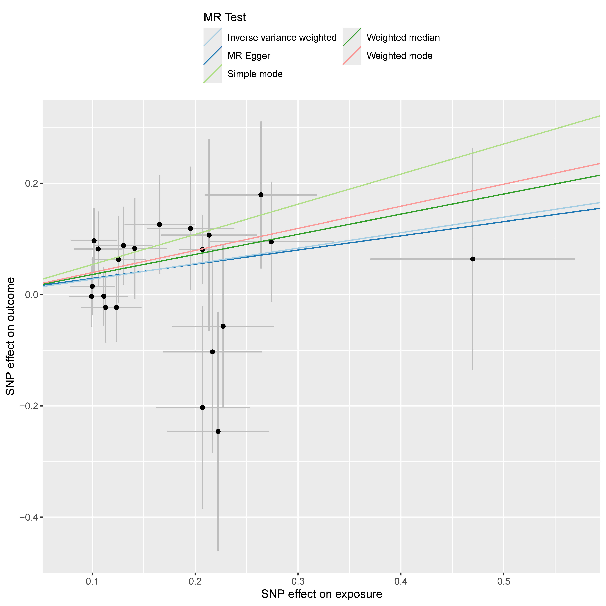
(9) (10)


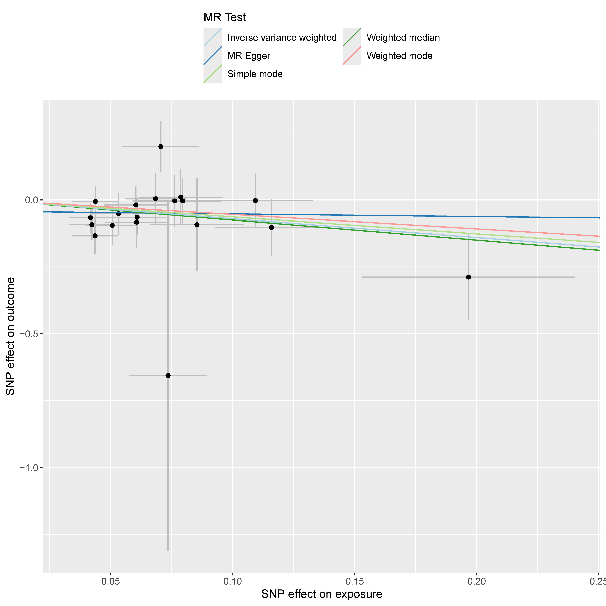


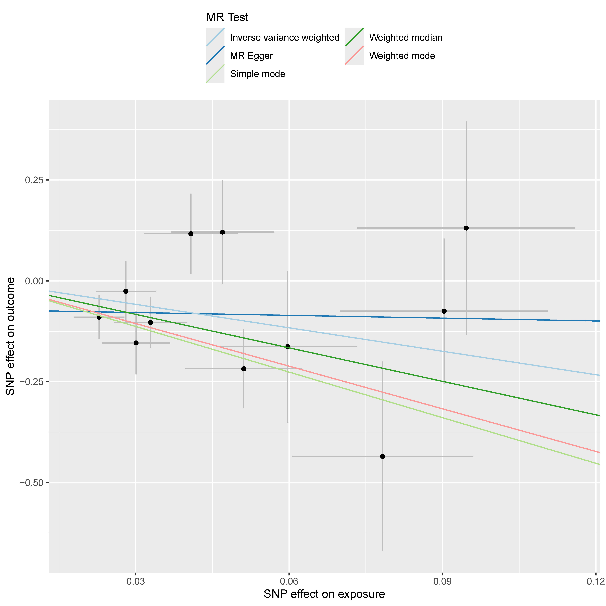
(11) (12)


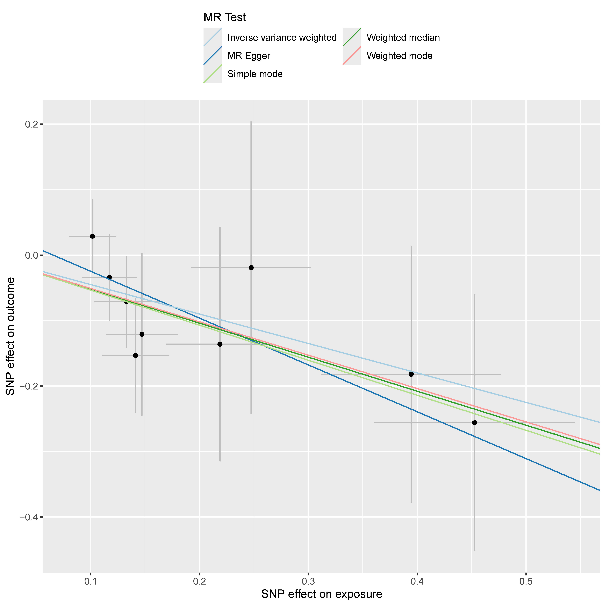


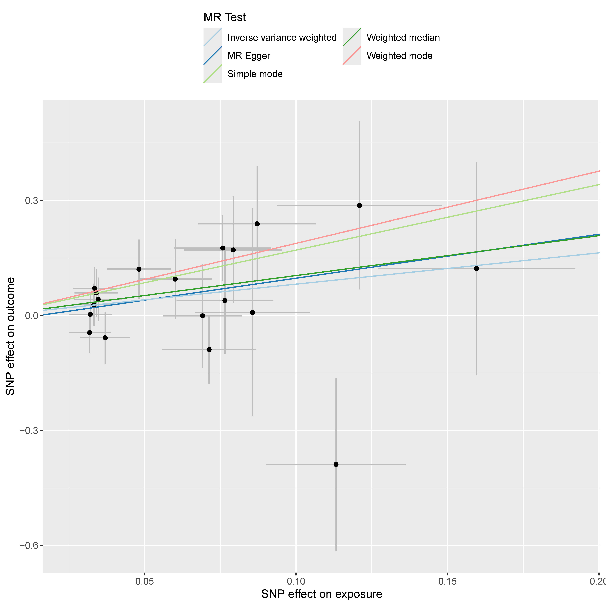
(13) (14
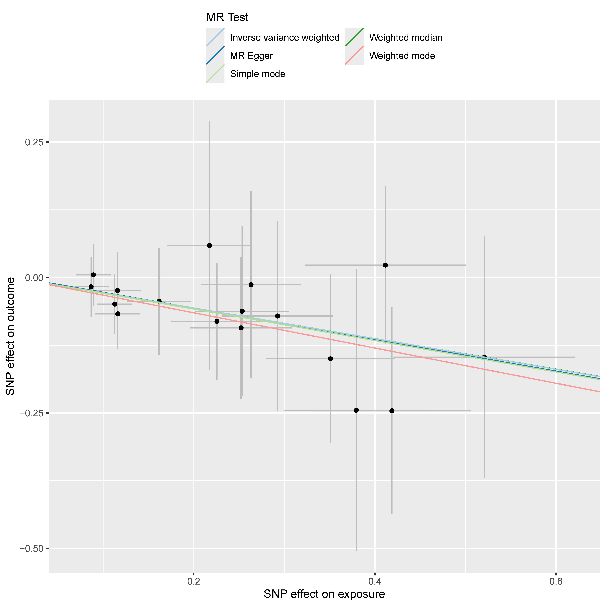
)


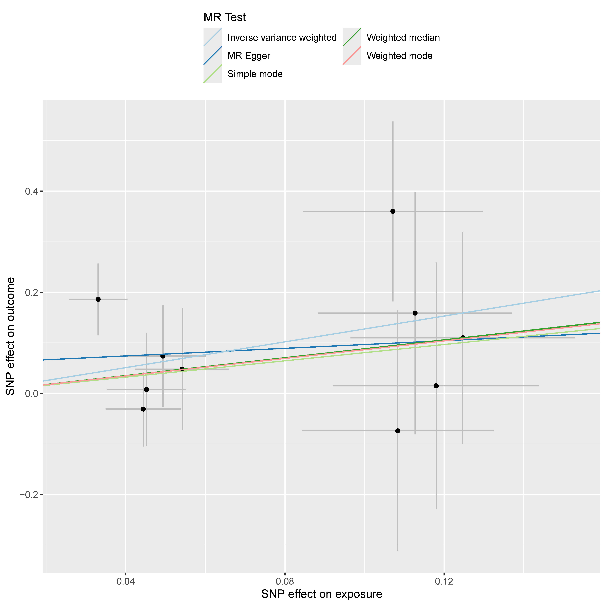

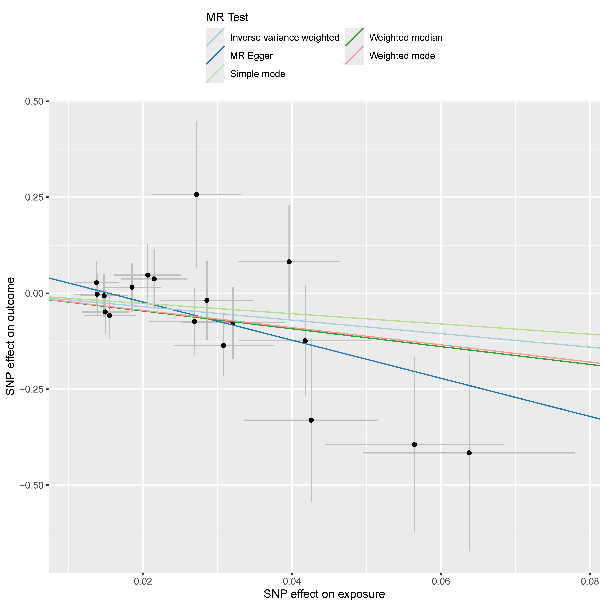
(15) (16)

(17) (18)


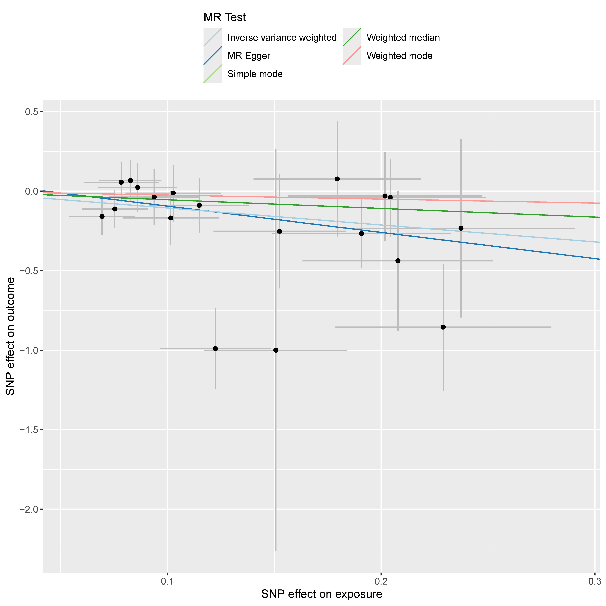

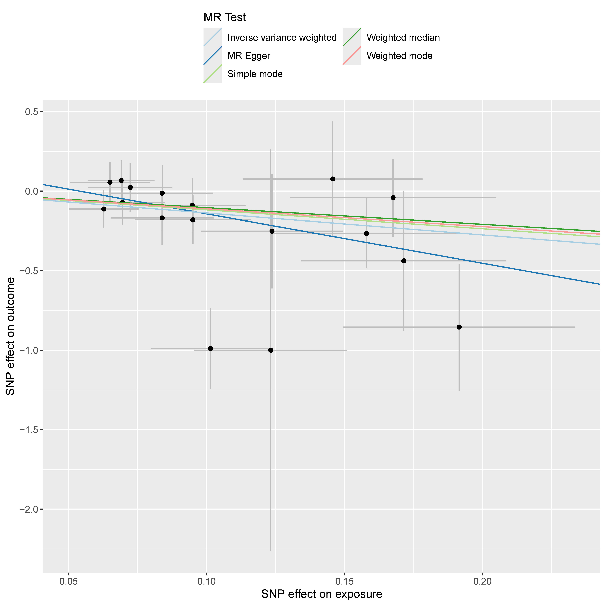


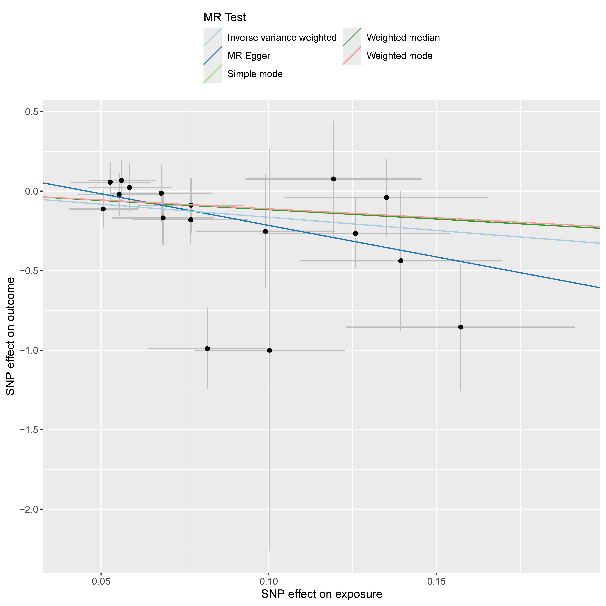
(19) (20)


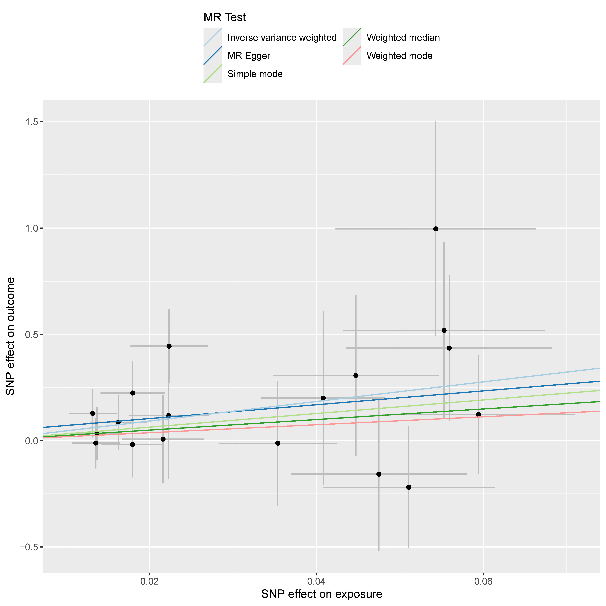


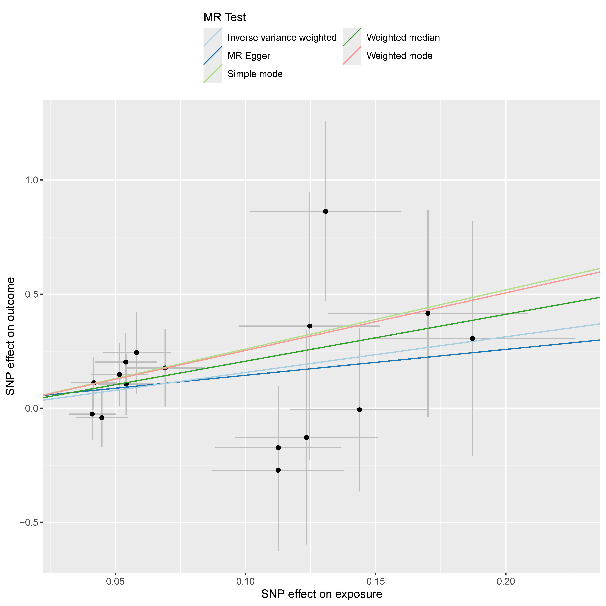
(21) (22
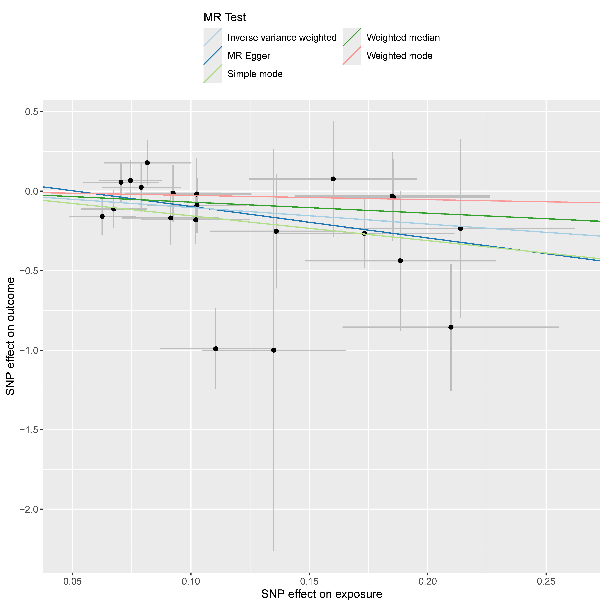
)


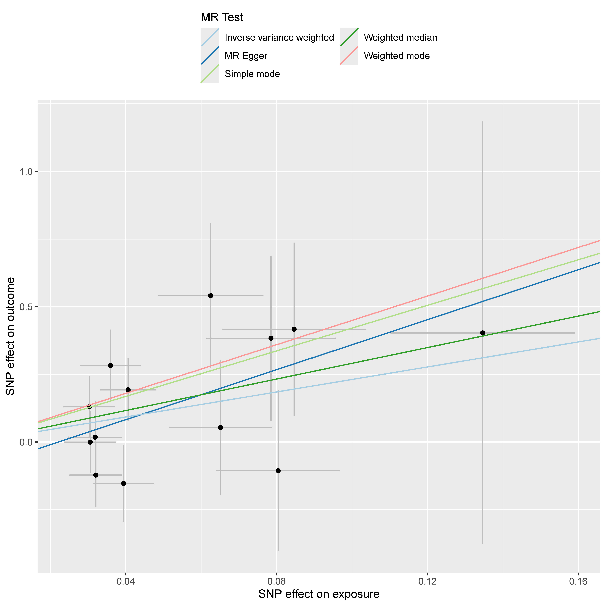
(23) (24)


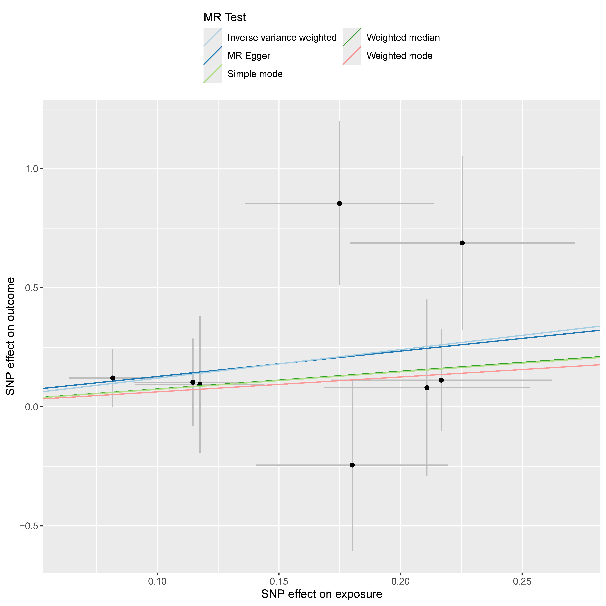


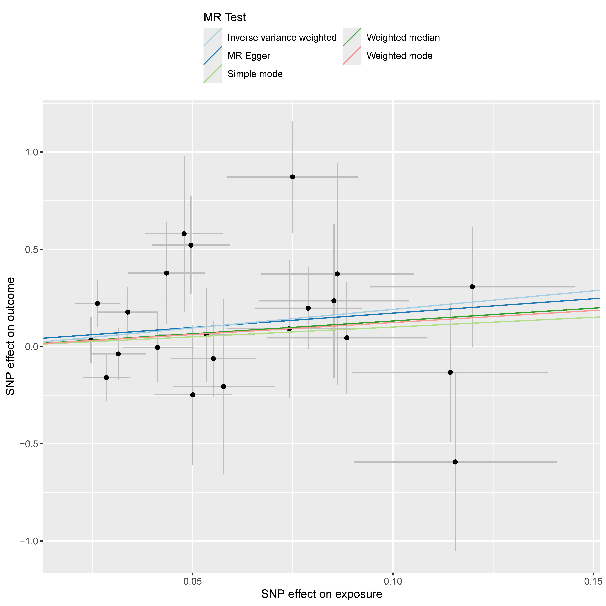
(25) (26
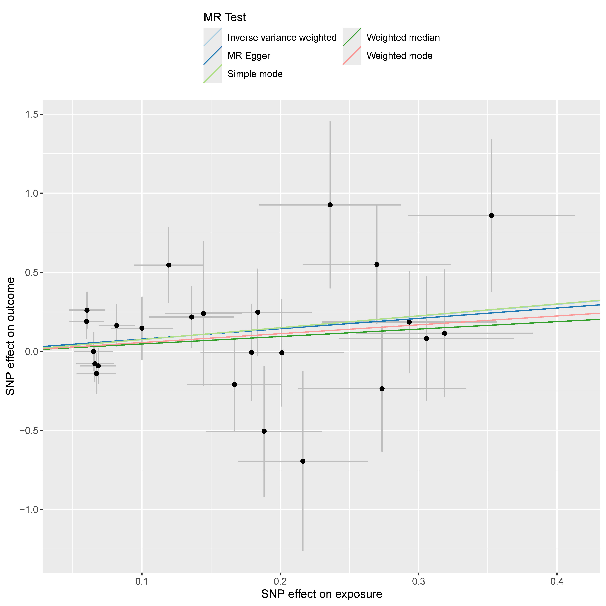
)


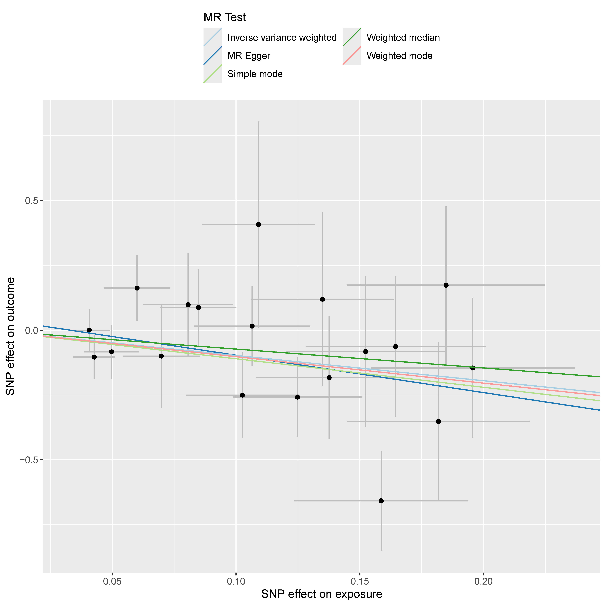
(27) (28)


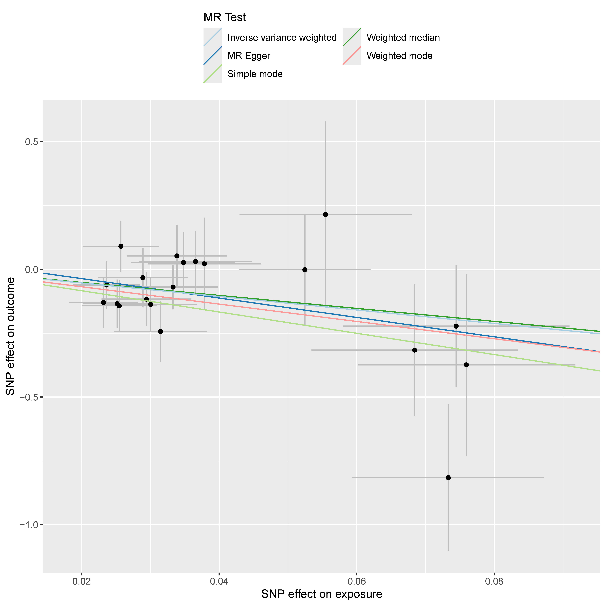


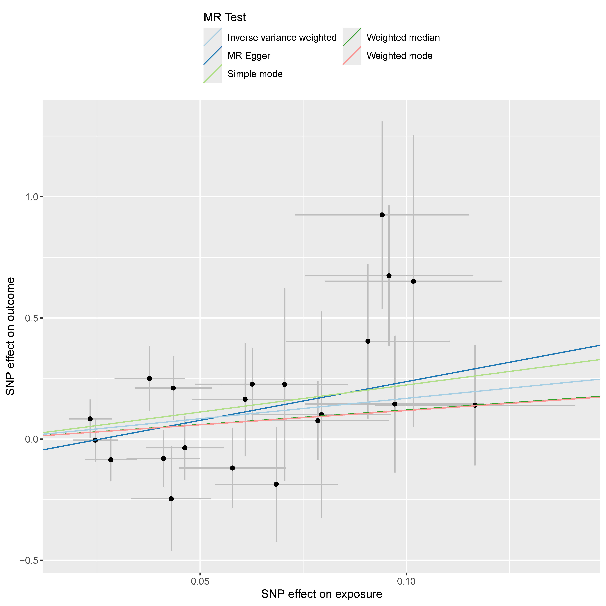
(29) (30)


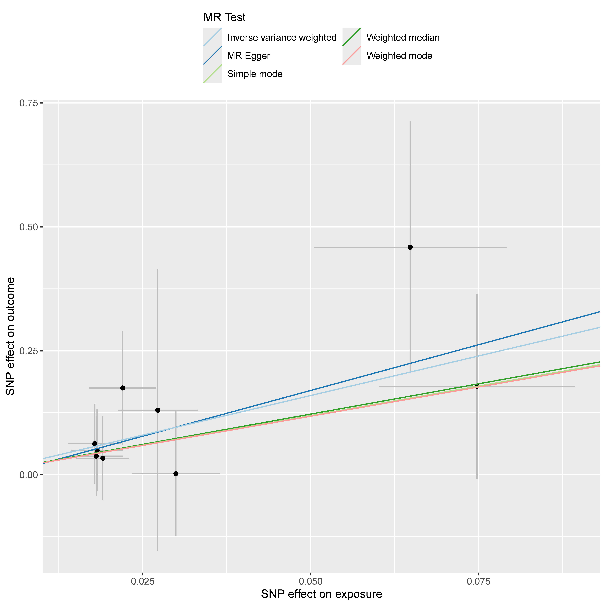


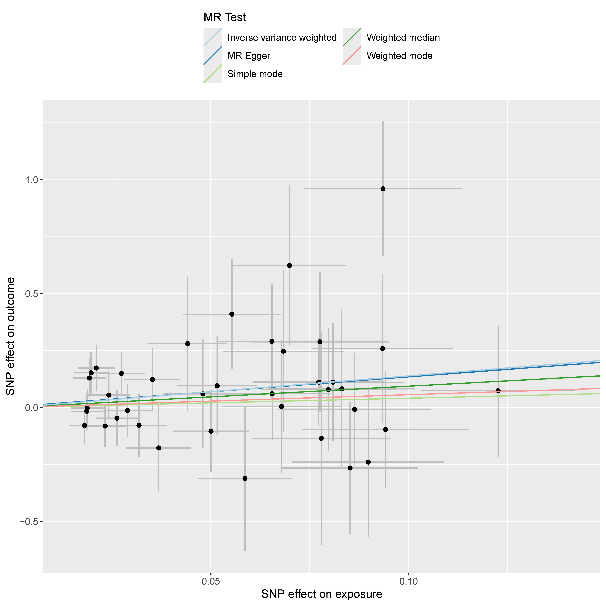
(31) (32)
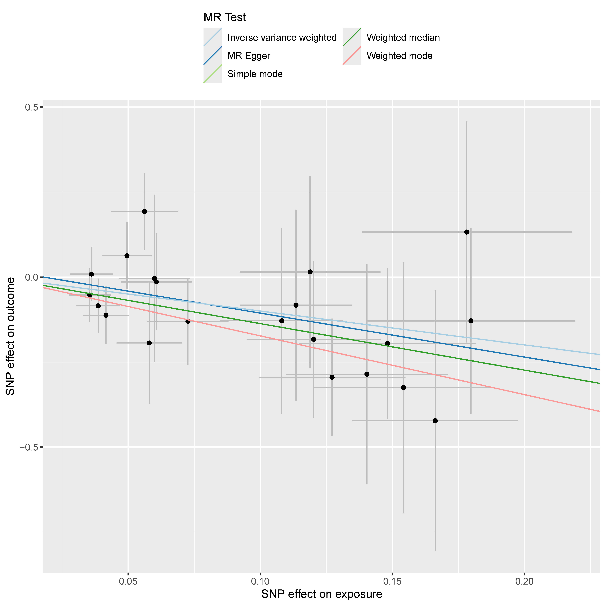


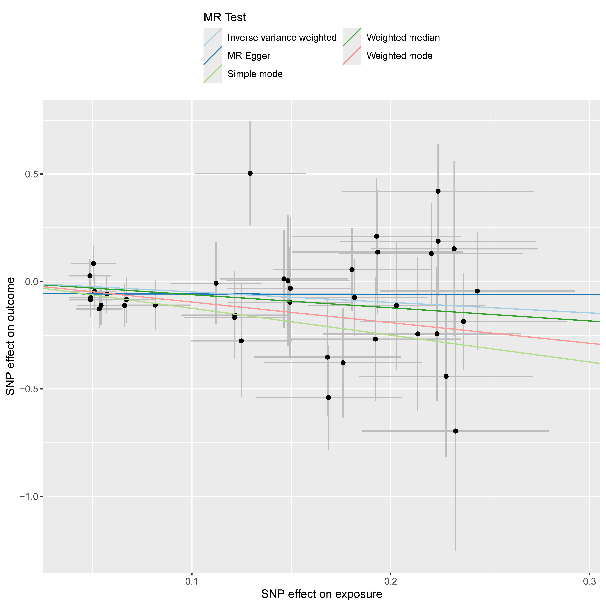
(33) (34
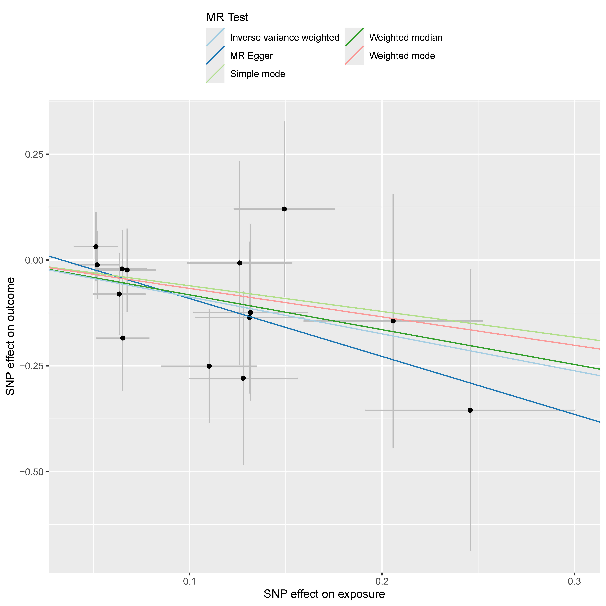
)


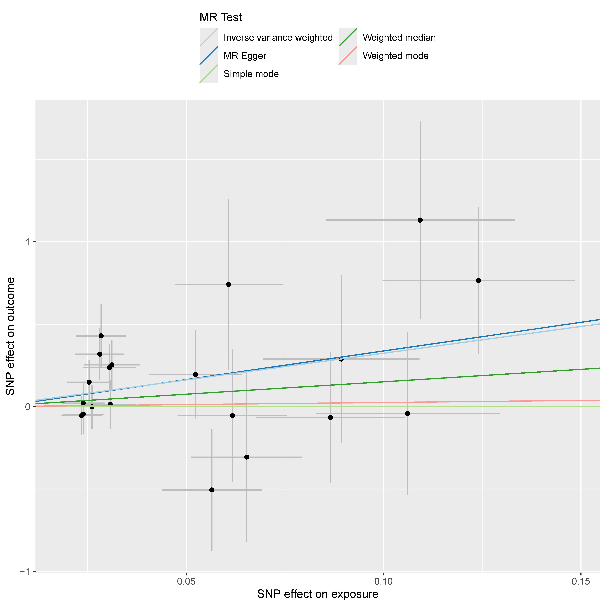
(35) (36)


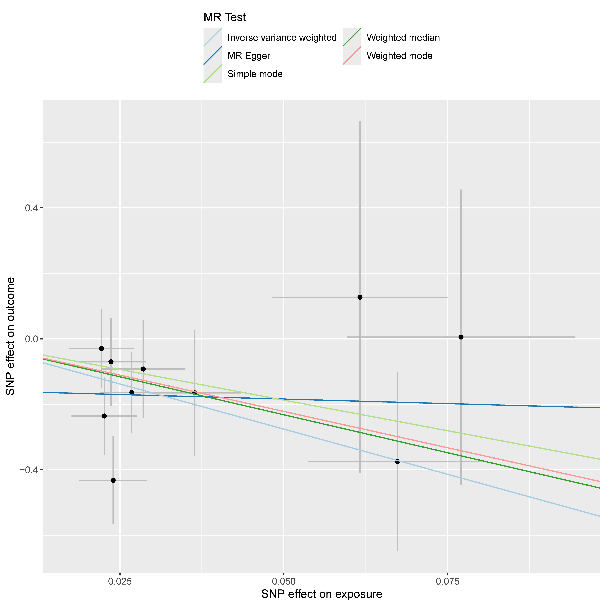


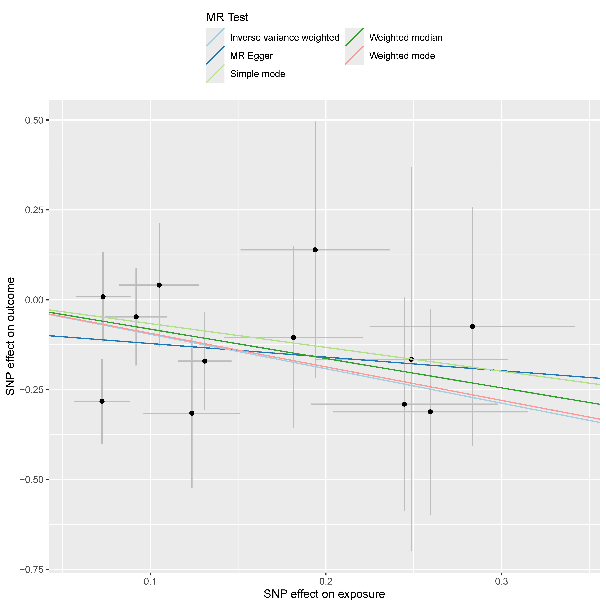
(37) (38
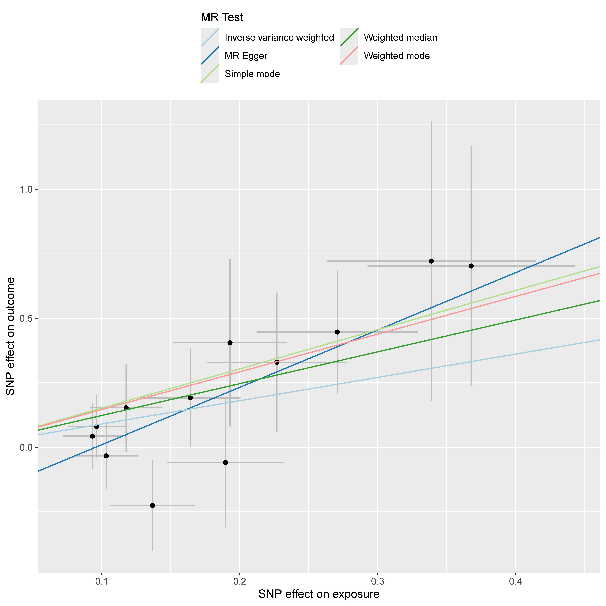
)


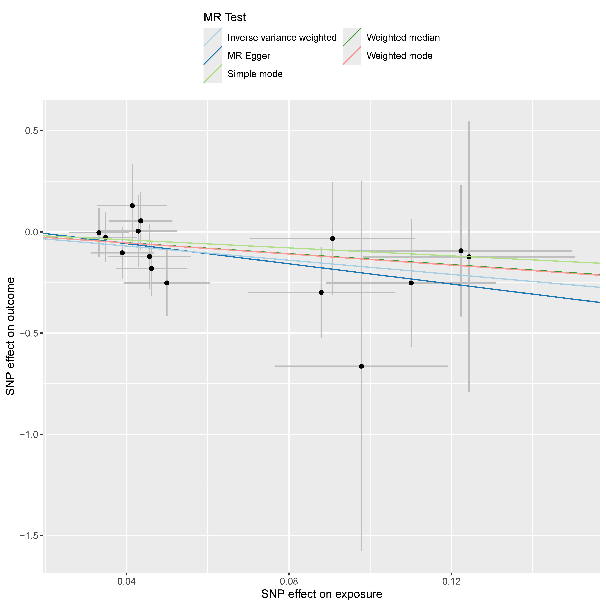
(39) (40)
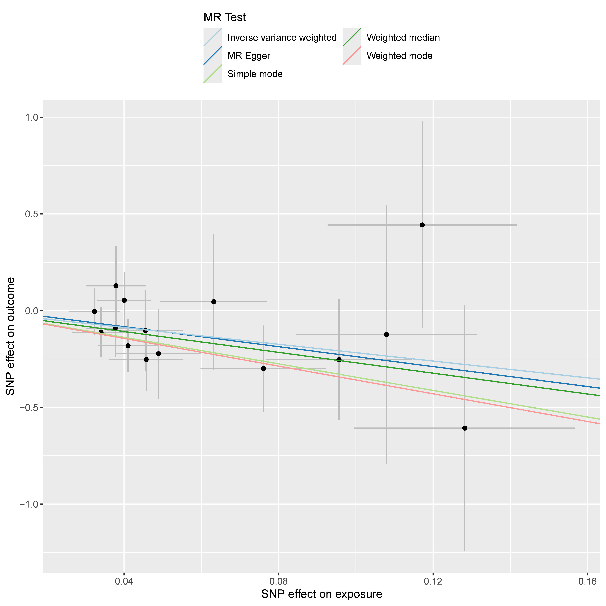


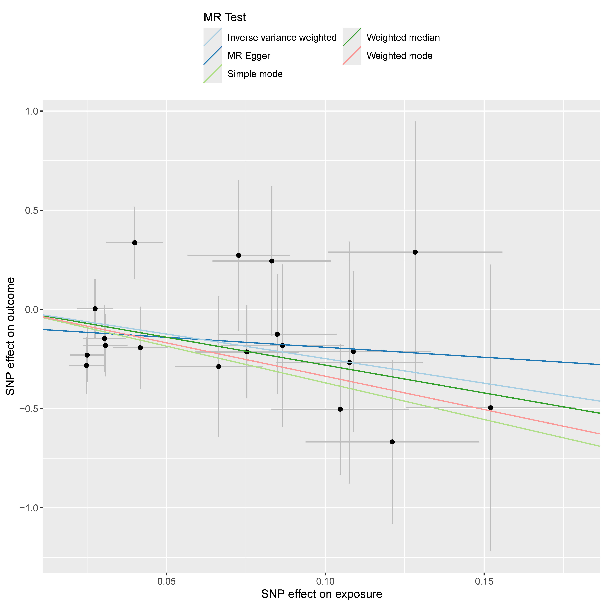
(41) (42)


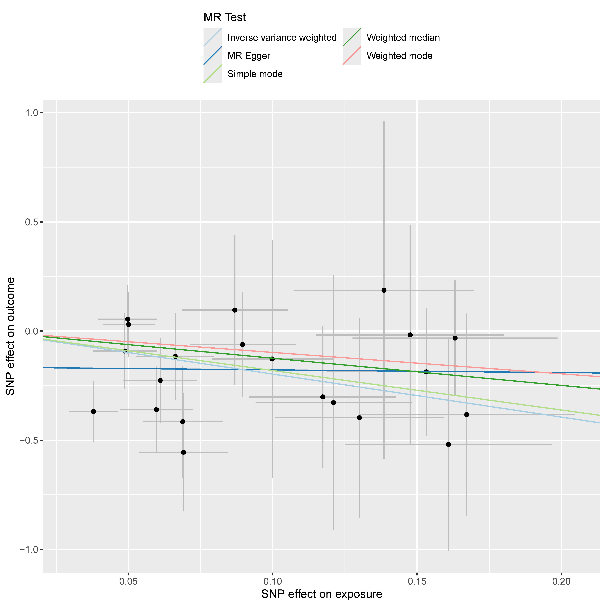


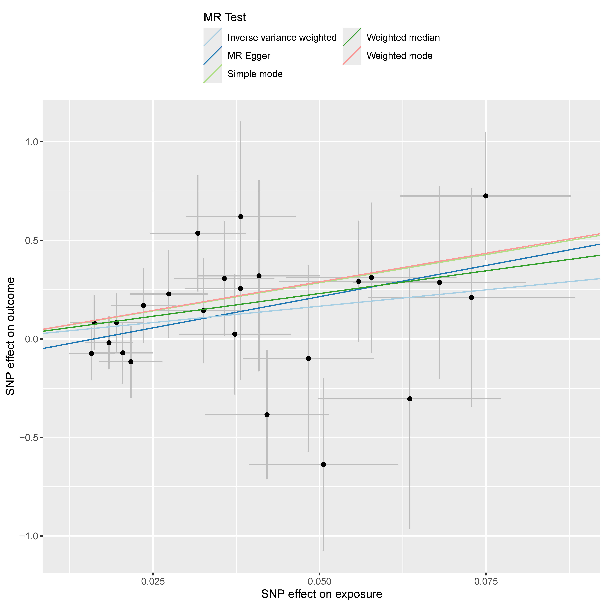
(43) (44
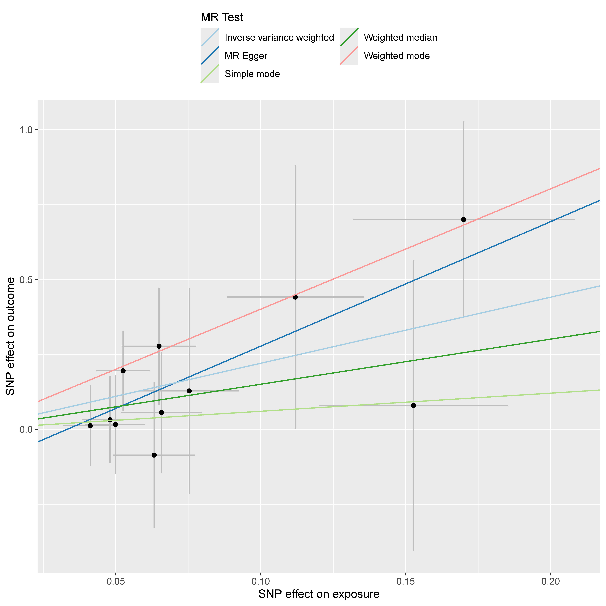
)

(45)


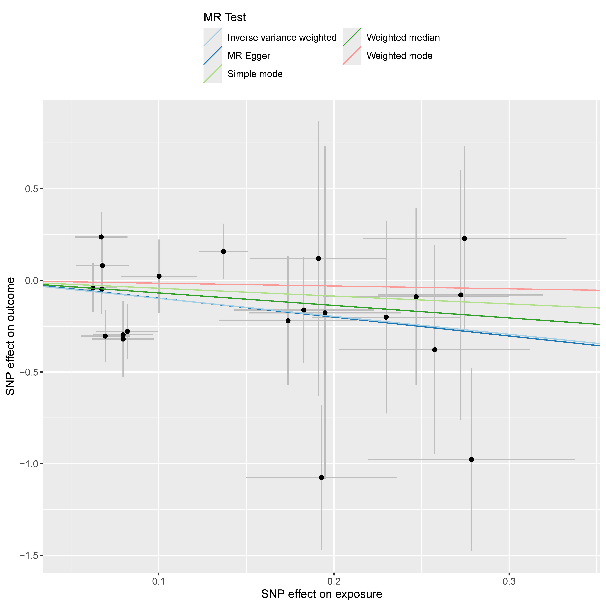

Supplement: Supplementary Figure S2 — Scatter plots for the effect of Gut microbiota on oral cancer. The ‘'exposure‘' markers in the figures, from (1) to (45), are labeled as Gluconobacter, Clostridium E sporosphaeroides, Omnitrophota, Faecalibacterium sp002160895, Mycoplasmataceae, Barnesiella, Dorea phocaeense, Escherichia, Provencibacterium, Bifidobacterium adolescentis, Bacteroides A, Azorhizobium, Akkermansia muciniphila B, Brachyspiraceae, Lachnoanaerobaculum saburreum, Eremiobacterota, Desulfovibrionia, Desulfovibrionaceae, Syntrophomonadia, Desulfobacterota A, Desulfovibrionales, Absiella dolichum, Veillonella, Geminocystis, Lactobacillus B ruminis, Providencia, Gemmatimonadaceae, Blautia A sp900066145, Hyphomonas, Sorangium, Clostridium M clostridioforme, Spirochaetia, Prevotella sp002933775, Megamonas funiformis, Francisellales, Bacillus AY, Lachnospira sp000437735, Bifidobacterium longum, Lachnospirales, Lachnospiraceae, Anaeromassilibacillus sp001305115, Pseudomonas aeruginosa, Merdibacter massiliensis, Chromobacteriaceae, and Bifidobacterium angulatum, while the ‘'outcome‘' markers are consistently represented as oral cancer. [file Data_Sheet_2.docx]

Figure. S3

(1) (2)


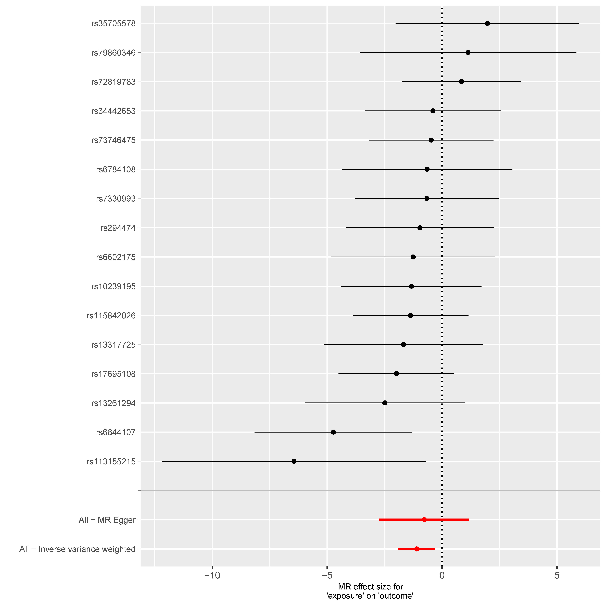

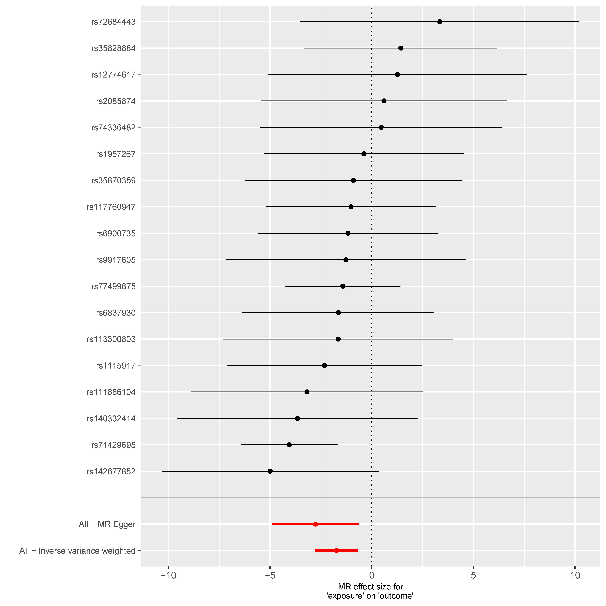


(3) (4
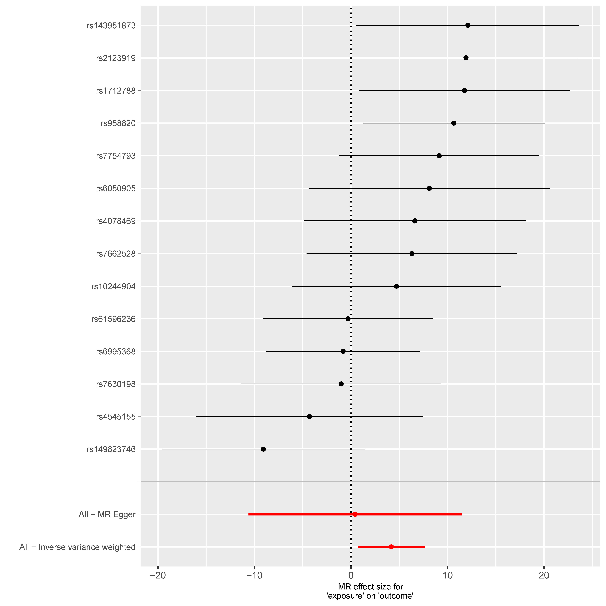
)


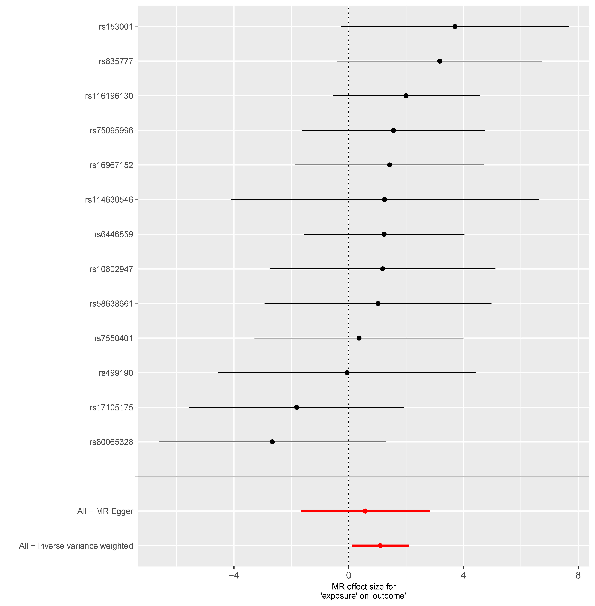


(5) (6)


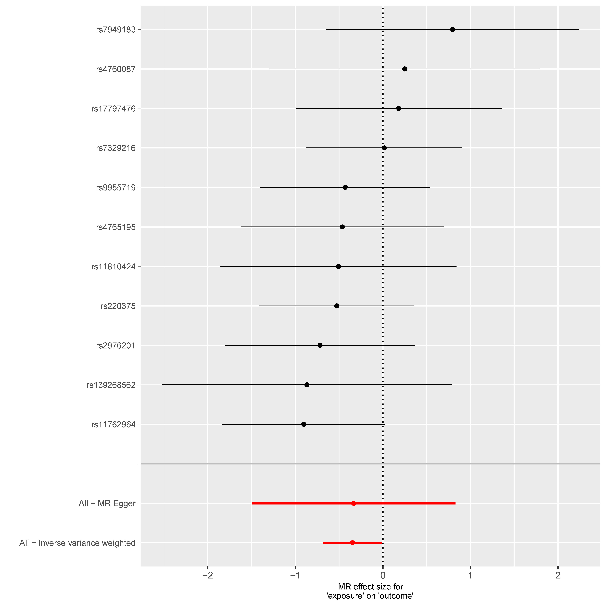

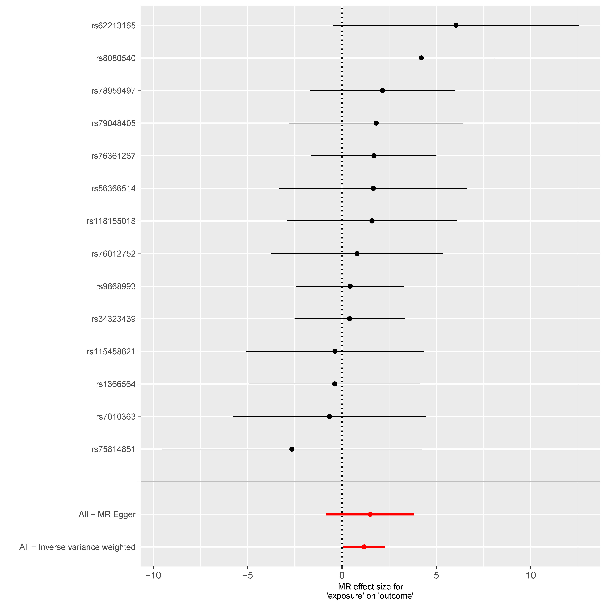


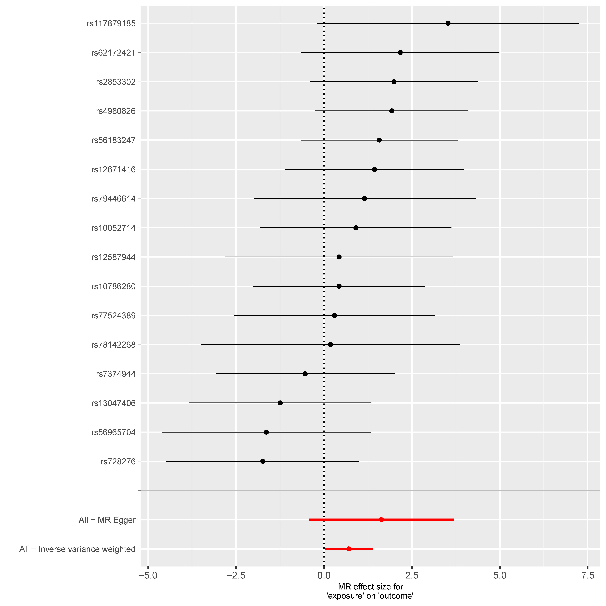

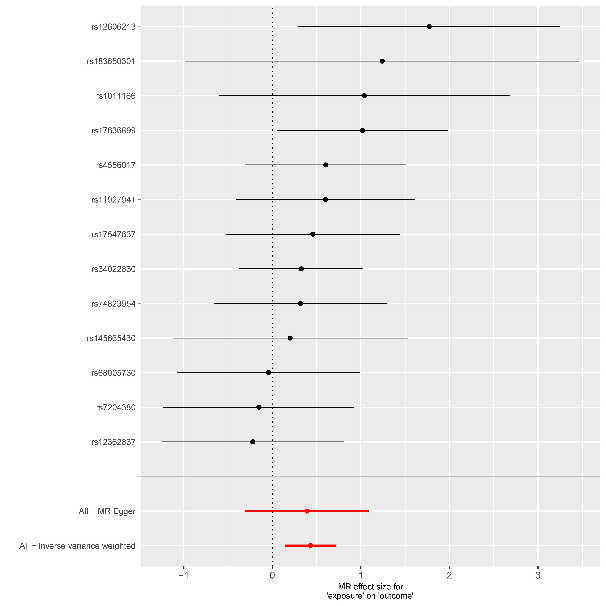
(7) (8)


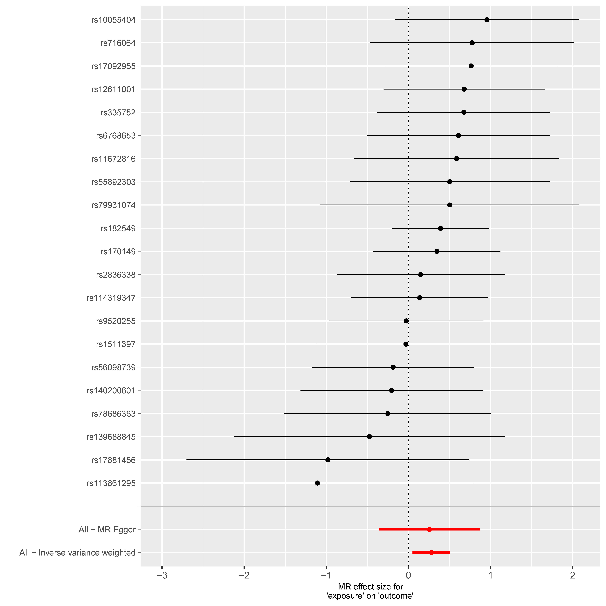
(9) (10)


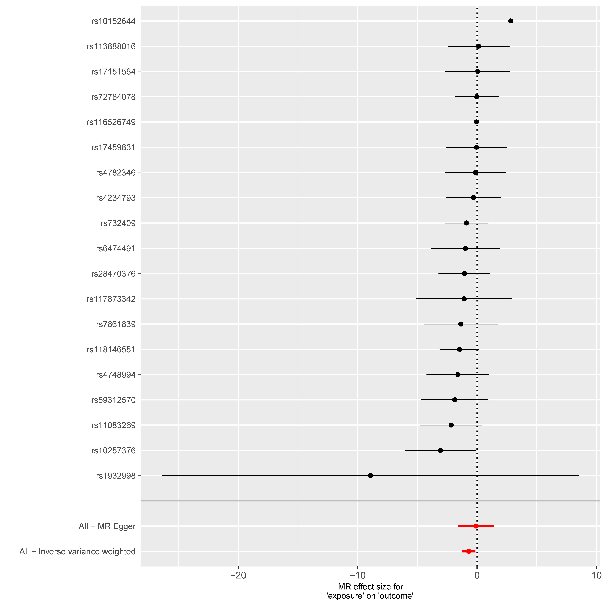


(11) (12)


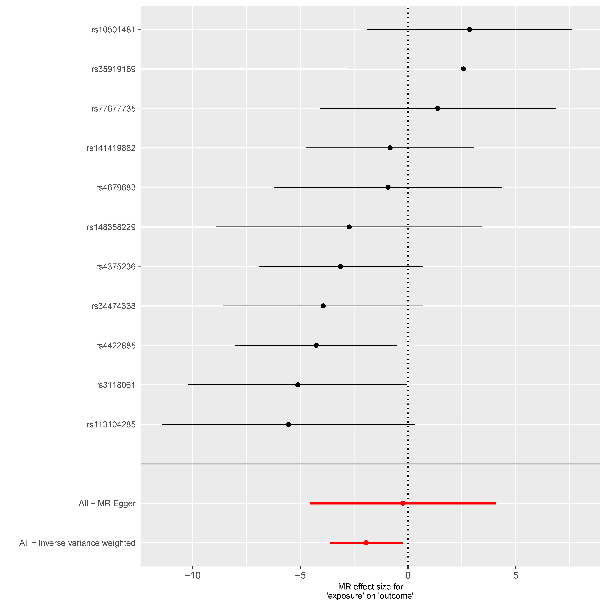

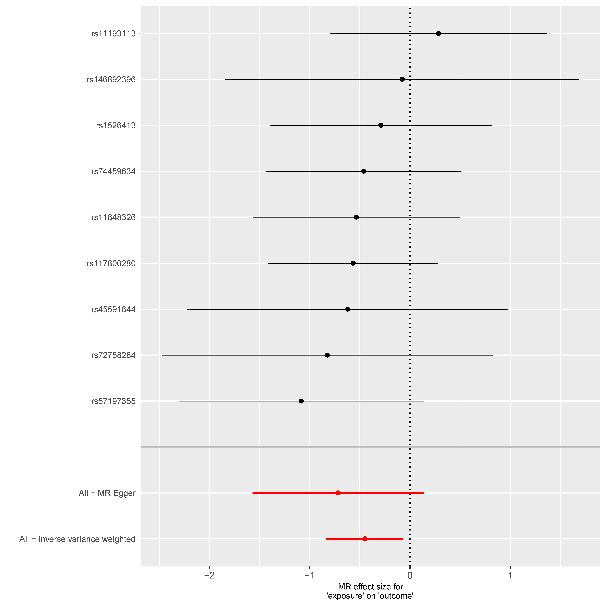


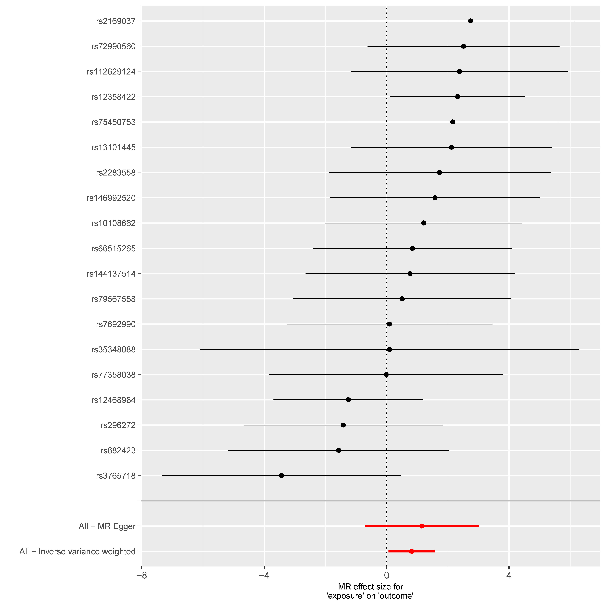
(13) (14)


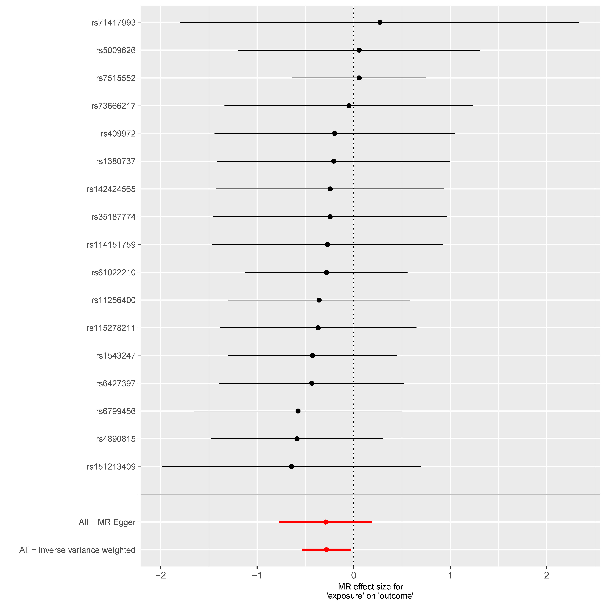


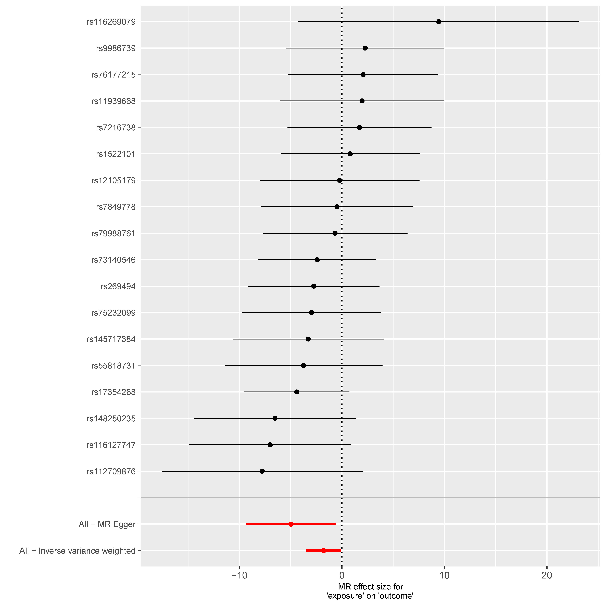
(15) (16)
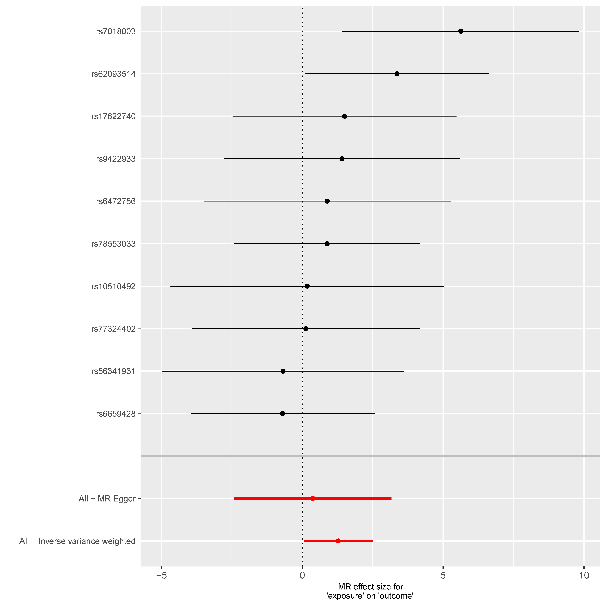


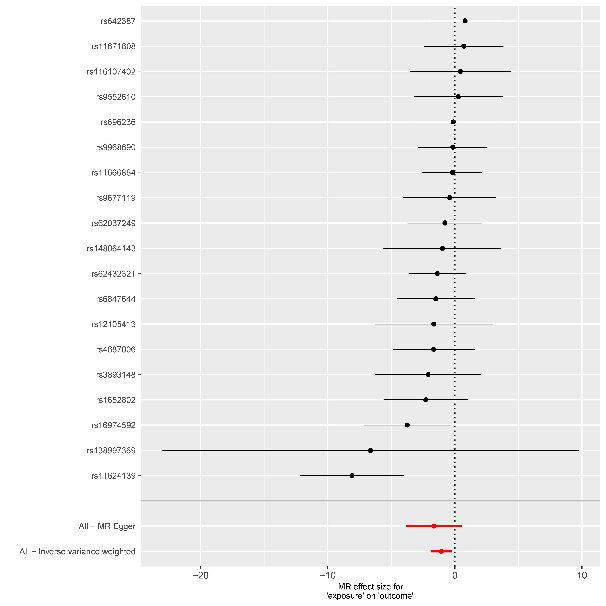
(17) (18)


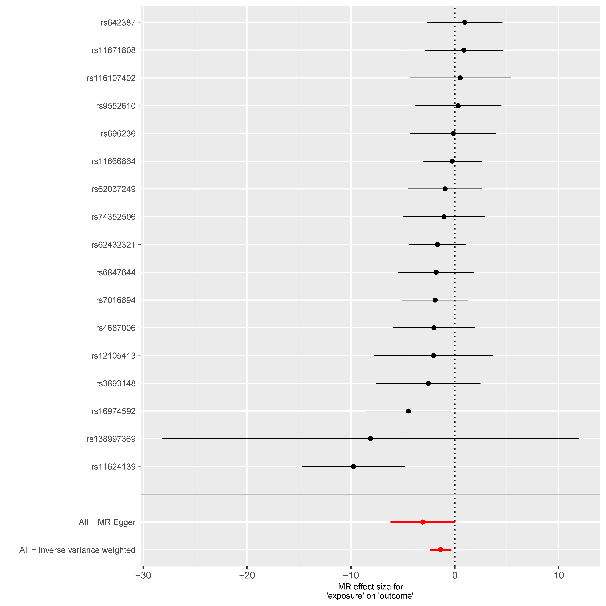


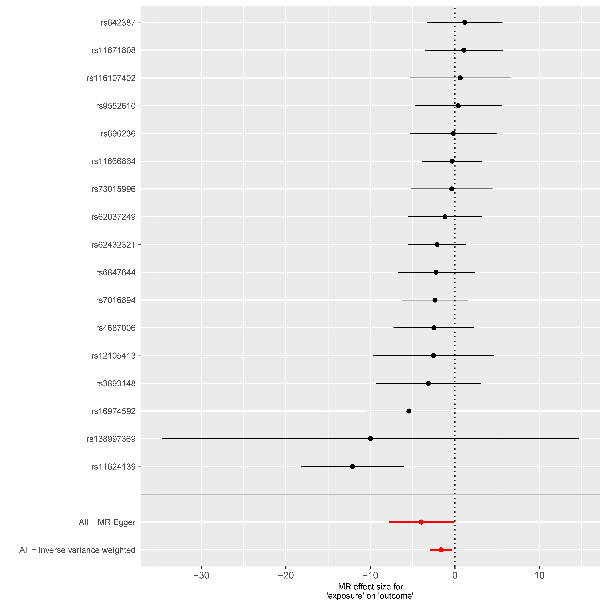
(19) (20)


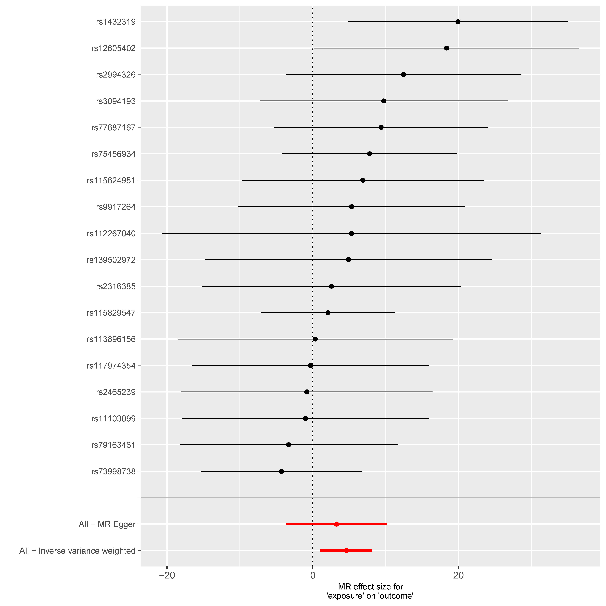


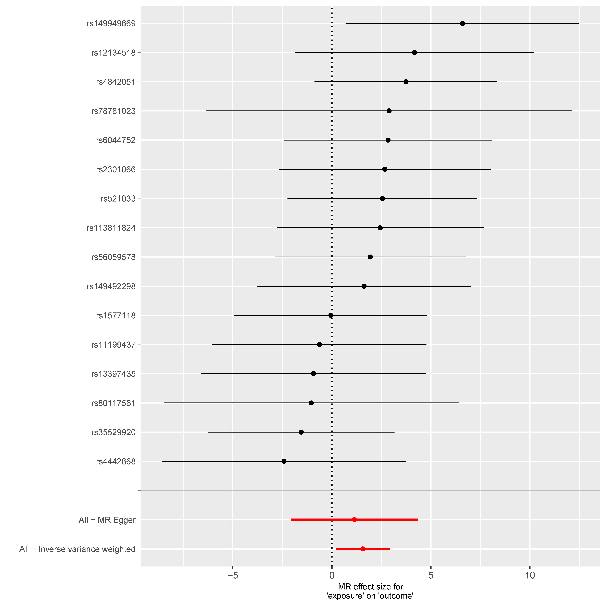
(21) (22
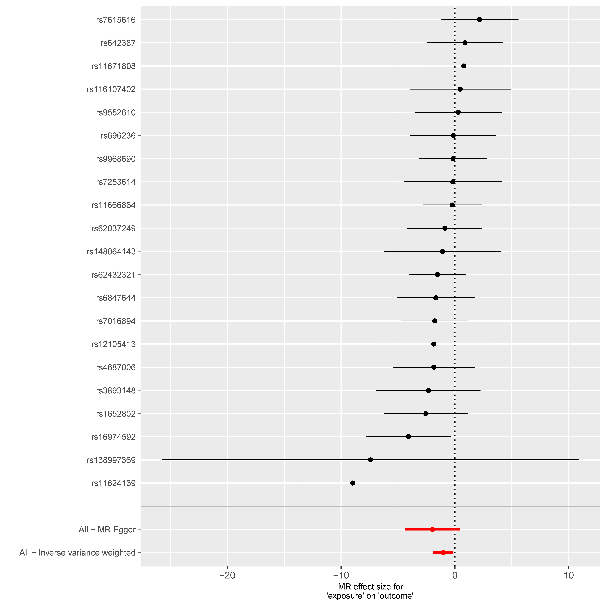
)


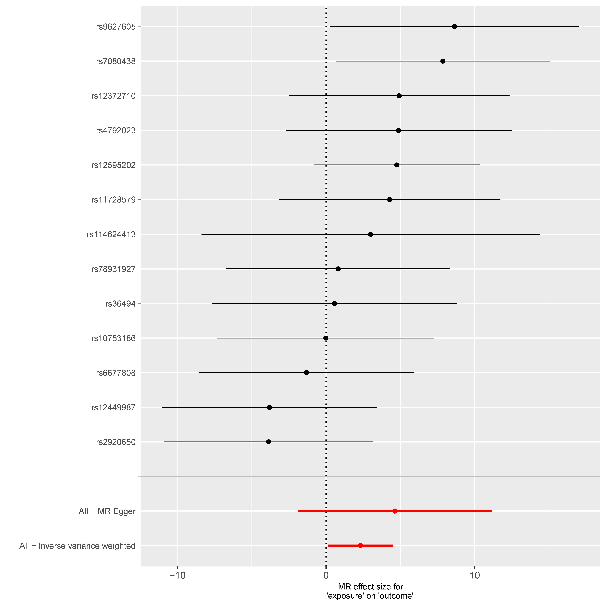
(23) (24)


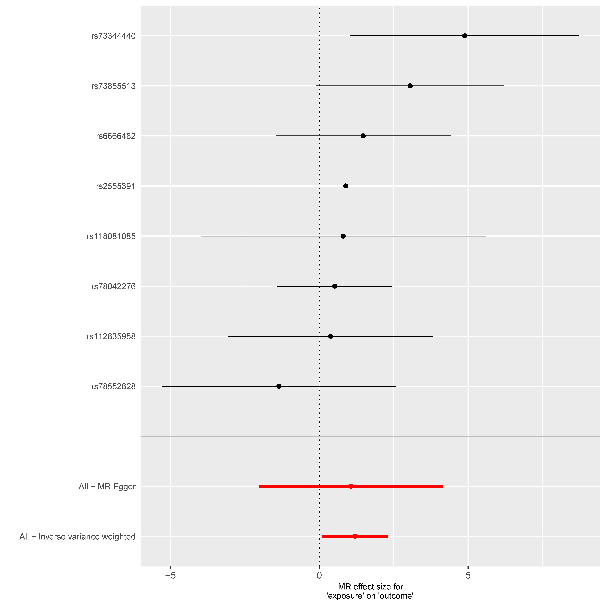


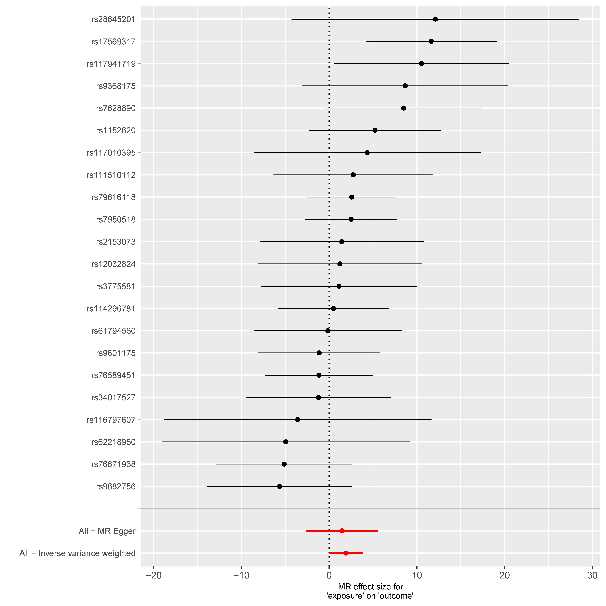
(25) (26)
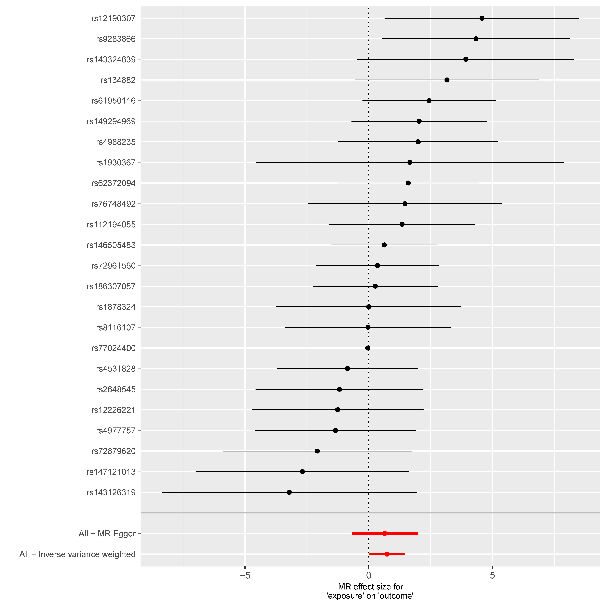


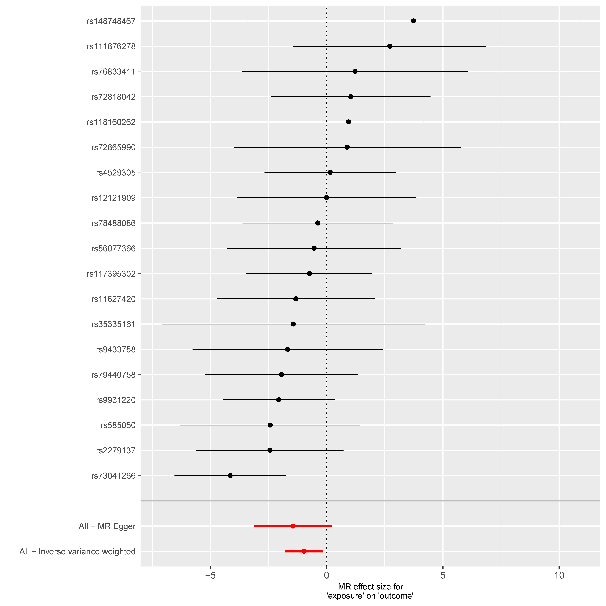
(27) (28)


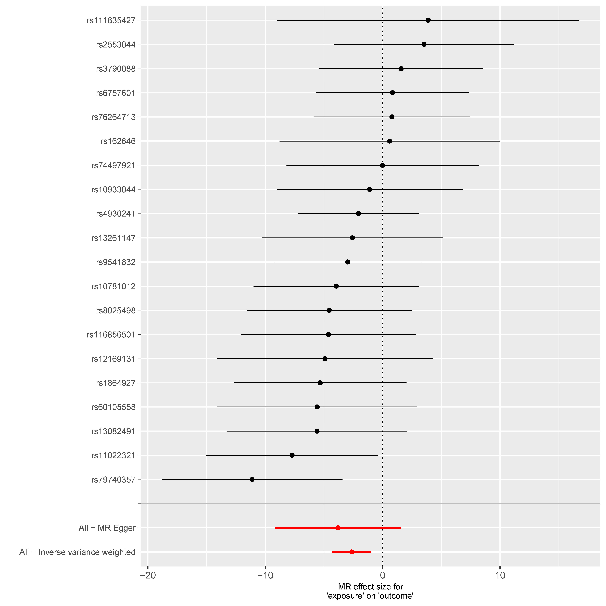


(29) (30)


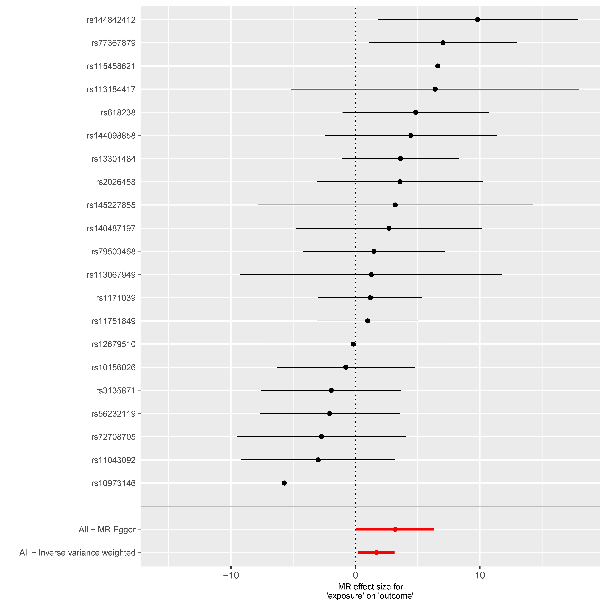

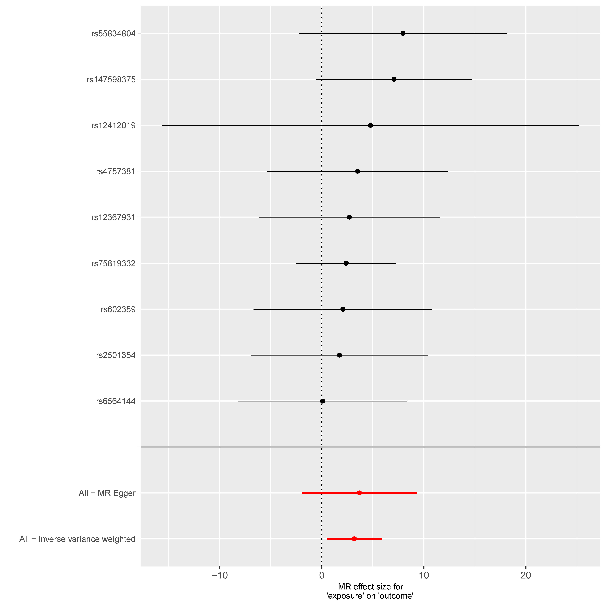


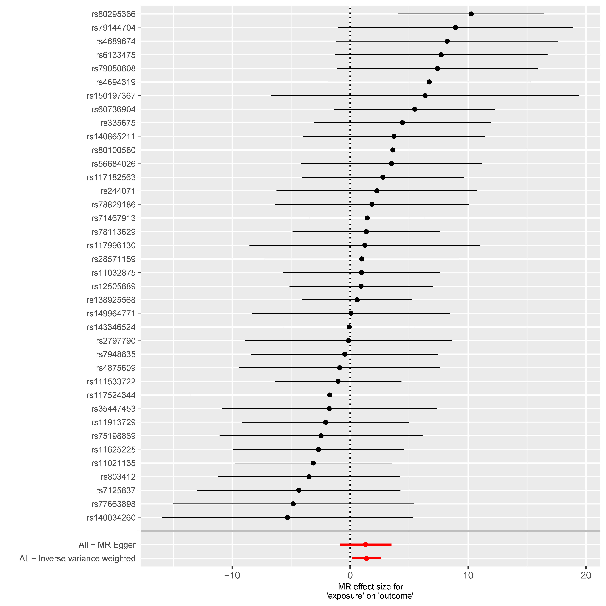
(31) (32)


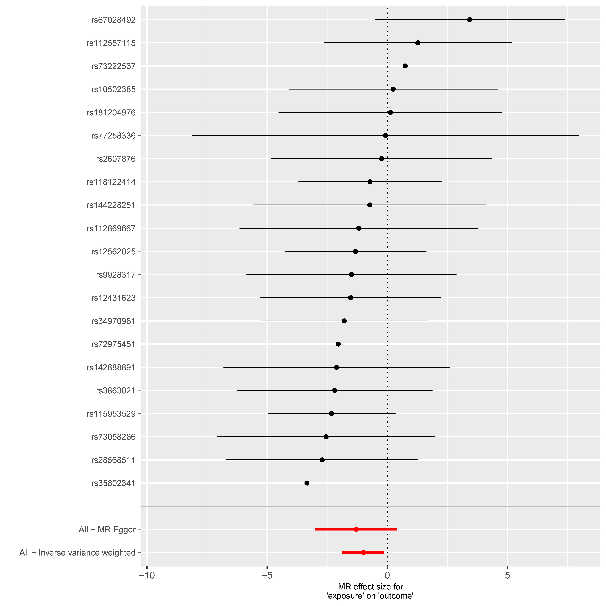


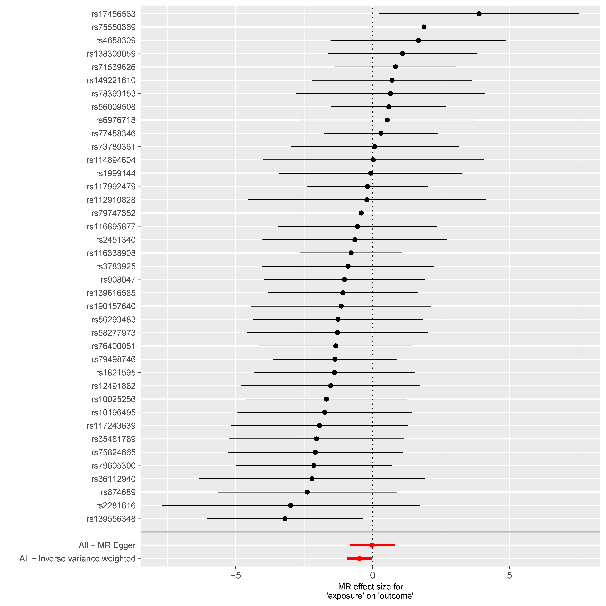
(33) (34)
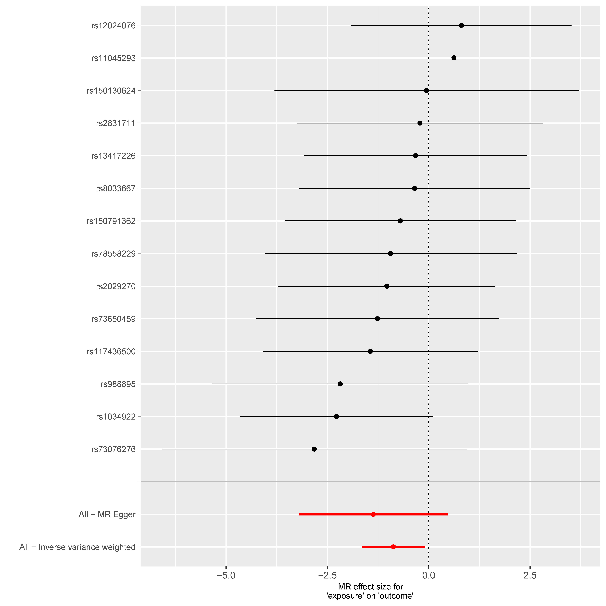


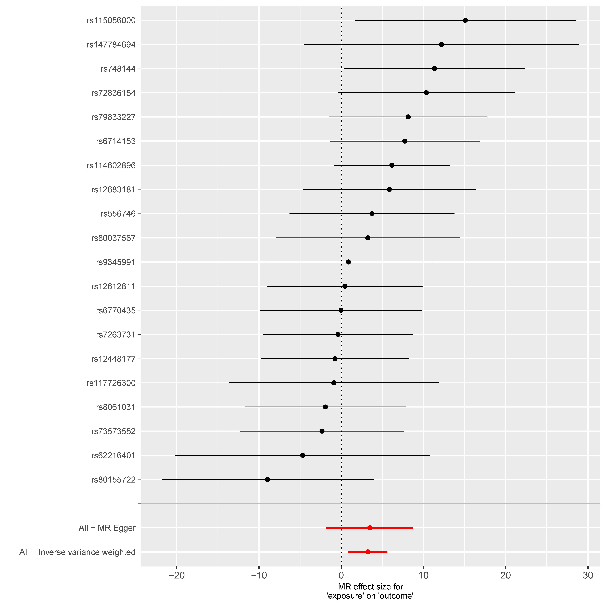
(35) (36)


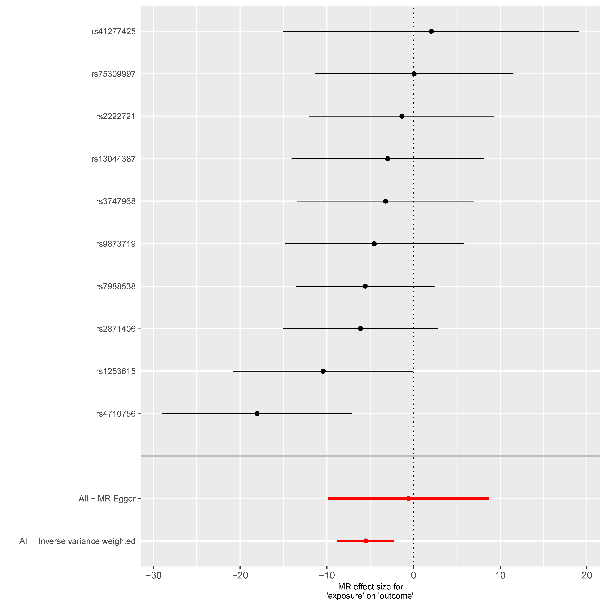


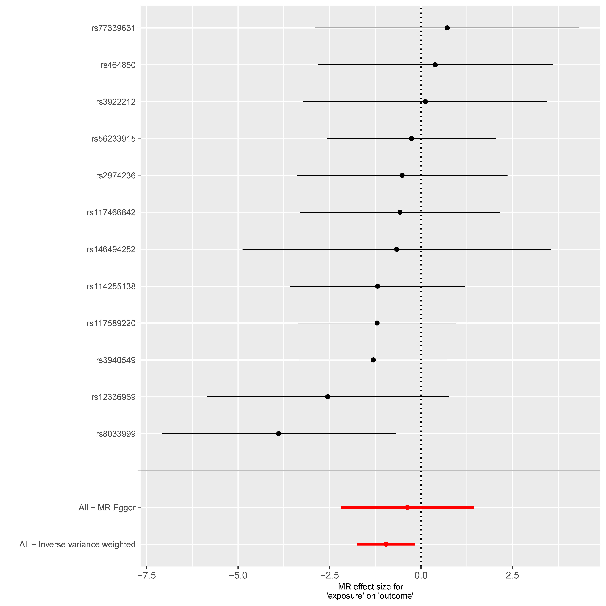
(37) (38)
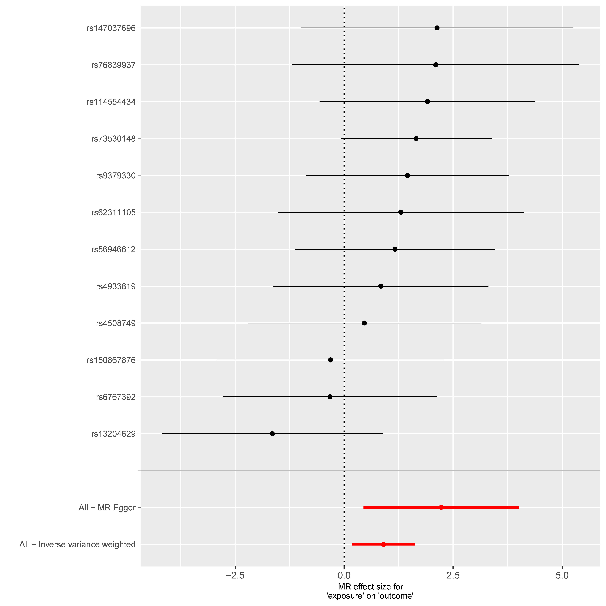


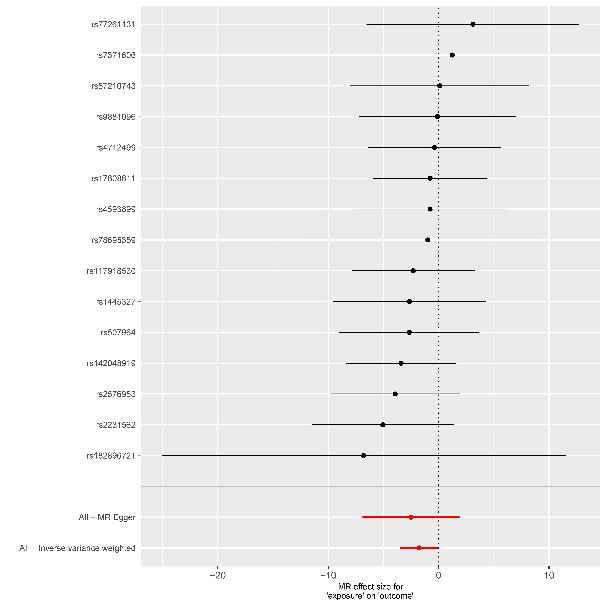
(39) (40)
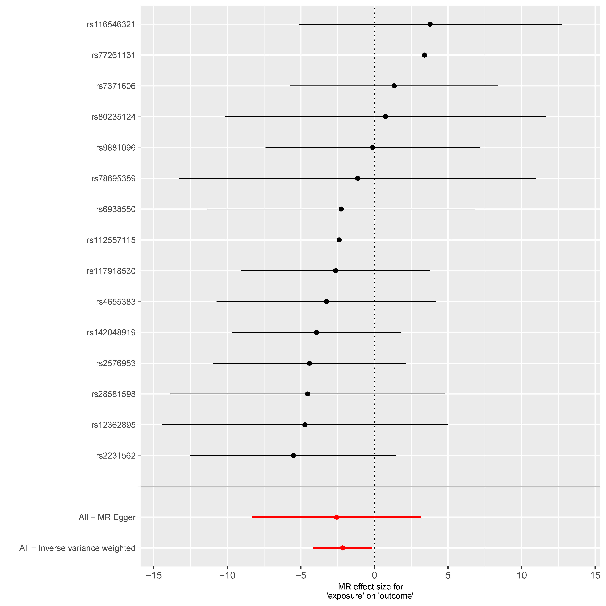


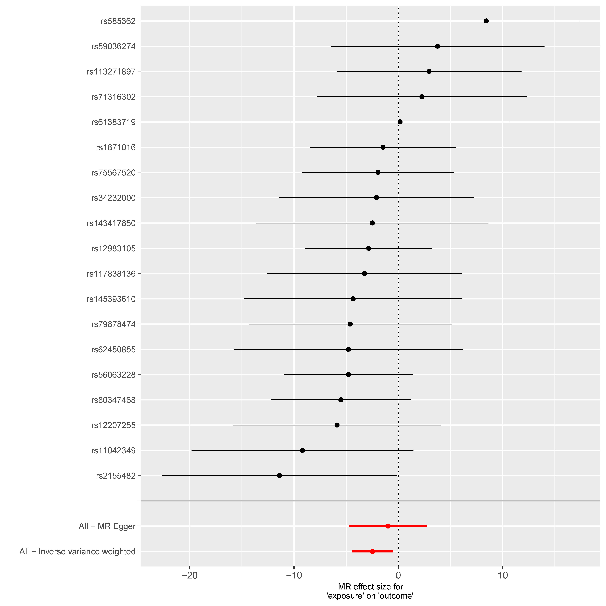
(41) (42)


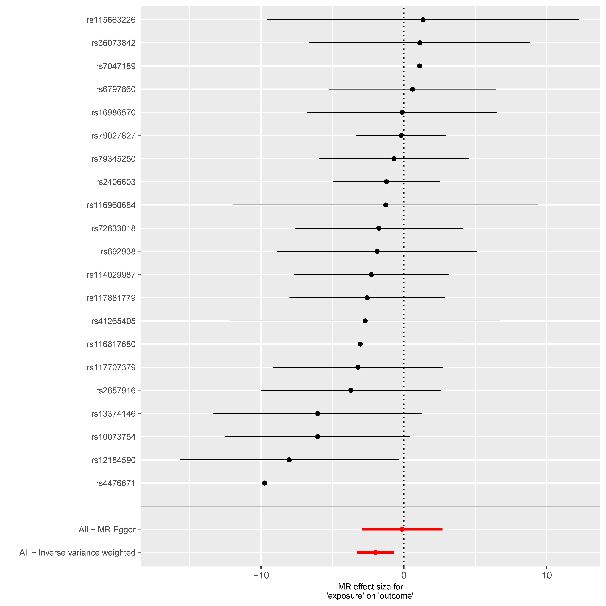


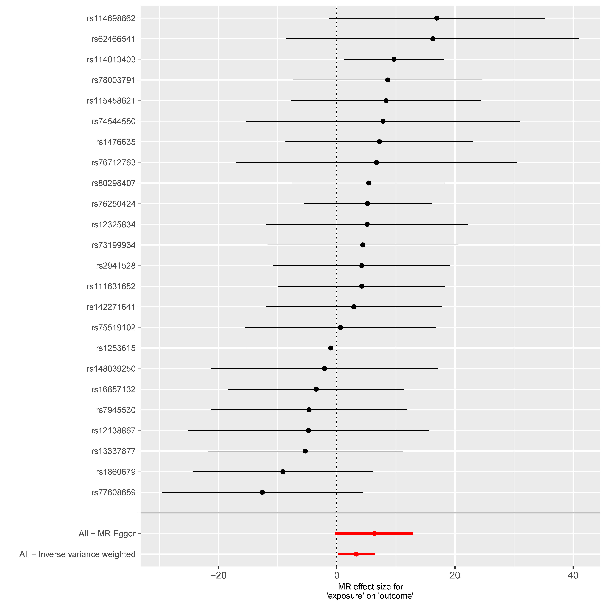
(43) (44
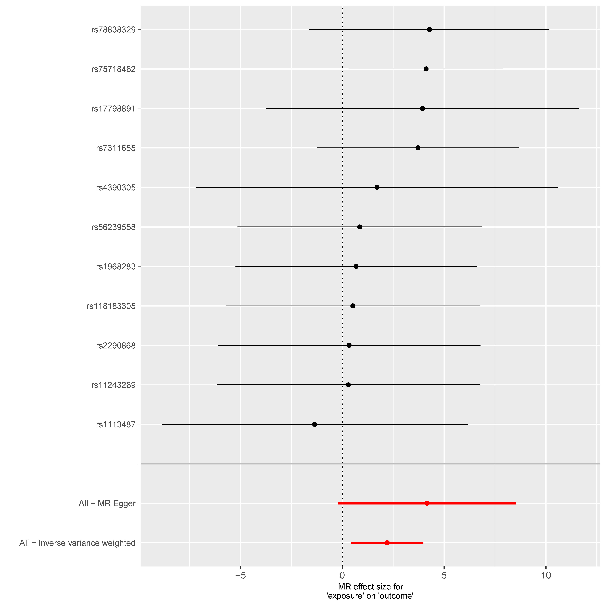
)

(45
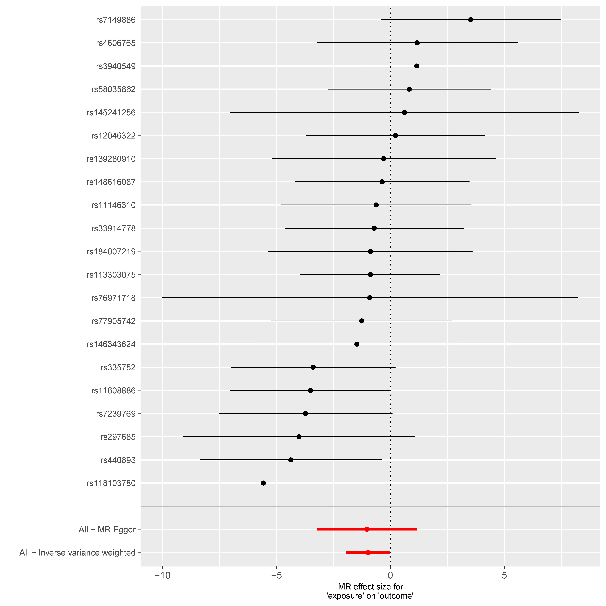
)

Supplement: Supplementary Figure S3 — Forest plots for the effect of Gut microbiota on oral cancer. The ‘'exposure‘' markers in the figures, from (1) to (45), are labeled as Gluconobacter, Clostridium E sporosphaeroides, Omnitrophota, Faecalibacterium sp002160895, Mycoplasmataceae, Barnesiella, Dorea phocaeense, Escherichia, Provencibacterium, Bifidobacterium adolescentis, Bacteroides A, Azorhizobium, Akkermansia muciniphila B, Brachyspiraceae, Lachnoanaerobaculum saburreum, Eremiobacterota, Desulfovibrionia, Desulfovibrionaceae, Syntrophomonadia, Desulfobacterota A, Desulfovibrionales, Absiella dolichum, Veillonella, Geminocystis, Lactobacillus B ruminis, Providencia, Gemmatimonadaceae, Blautia A sp900066145, Hyphomonas, Sorangium, Clostridium M clostridioforme, Spirochaetia, Prevotella sp002933775, Megamonas funiformis, Francisellales, Bacillus AY, Lachnospira sp000437735, Bifidobacterium longum, Lachnospirales, Lachnospiraceae, Anaeromassilibacillus sp001305115, Pseudomonas aeruginosa, Merdibacter massiliensis, Chromobacteriaceae, and Bifidobacterium angulatum, while the ‘'outcome‘' markers are consistently represented as oral cancer. [file Data_Sheet_3.docx]

Figure. S4


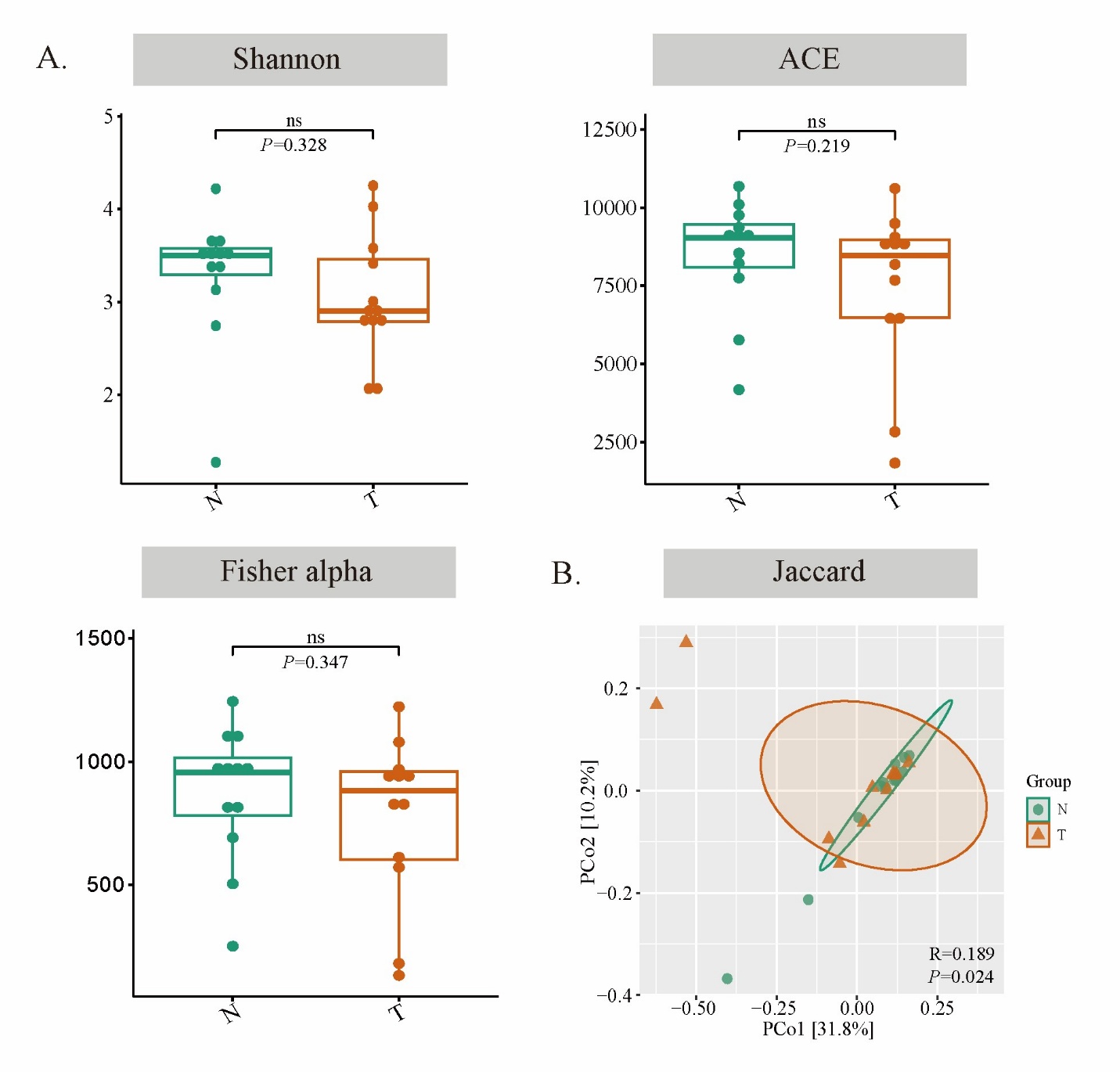

Supplement: Supplementary Figure S4 — Alpha-diversity and beta-diversity in oral cancer patients (T) and healthy controls (N). (A) Alpha-diversity (based on Shannon, ACE, and Fisher alpha). (B) PCoA was conducted to assess beta diversity based on the Jaccard metric distance. [file Data_Sheet_4.docx]
